# Checklist of the Coleoptera of New Brunswick, Canada

**DOI:** 10.3897/zookeys.573.8022

**Published:** 2016-03-24

**Authors:** Reginald P. Webster

**Affiliations:** 1 24 Mill Stream Drive, Charters Settlement, NB, Canada E3C 1X1

**Keywords:** Checklist, Coleoptera, New Brunswick, Canada

## Abstract

All 3,062 species of Coleoptera from 92 families known to occur in New Brunswick, Canada, are recorded, along with their author(s) and year of publication using the most recent classification framework. Adventive and Holarctic species are indicated. There are 366 adventive species in the province, 12.0% of the total fauna.

## Introduction

The first checklist of the beetles of Canada by Bousquet (1991) listed 1,365 species from the province of New Brunswick, Canada. Since that publication, many species have been added to the faunal list of the province, primarily from increased collection efforts and examination of specimens contained in museum collections by Christopher Majka and Reginald Webster (Webster et al. 2016). Many of these new records were published in papers by Webster and co-authors, Majka and co-authors, and Klimaszewski and co-authors in the following four special ZooKeys issues: ZooKeys 2 (2008) Biodiversity, Biosystematics, and Ecology of Canadian Coleoptera, edited by Christopher Majka and Jan Klimaszewski; ZooKeys 22 (2009), Biodiversity, Biosystematics, and Ecology of Canadian Coleoptera II, edited by Christopher Majka and Jan Klimaszewski; ZooKeys 179 (2012) Biodiversity and Ecology of the Coleoptera of New Brunswick, Canada, edited by Robert Anderson and Jan Klimaszewski, and ZooKeys 186 (2012); Biosystematics and Ecology of Canadian Staphylinidae (Coleoptera) II., edited by Jan Klimaszewski and Robert Anderson. Other new records for New Brunswick were included in other papers by Webster and co-authors, Klimaszewki and co-authors, and Majka and co-authors that treated the fauna from the Maritime Provinces and Canada. See Webster et al. (2016) in this special issue for details on the history of collecting in New Brunswick. New records from the above publications and other sources added 1,338 species to the first checklist of beetles of Canada (Bousquet 1991), nearly doubling the number of species and subspecies listed as occurring in New Brunswick (Bousquet et al. 2013). Since the publication of Bousquet et al. (2013) and before this current special issue of Zookeys, an additional 52 species were added to the faunal list of New Brunswick from new species descriptions and new records (Webster et al. 2016).

In this special issue, 303 species and one subspecies are newly recorded for New Brunswick. Among the new records are 32 species new to science, four new North American records, 21 new Canadian records, 270 new provincial records, and 45 adventive species. Three species were removed from the provincial list, and one species was reinstated that was erroneously not included for New Brunswick by Bousquet et al. (2013). This brings the number of species known from New Brunswick to 3,062. This is 13% more than listed for New Brunswick by Bousquet et al. (2013) and a 124% increase since the publication of Bousquet (1991).

## Format

The classification of the family-group taxa used in this checklist follows Bouchard et al. (2011), except for the Hydrophiloidea, which follows Short and Fikáček (2013). The order used in the checklist is phylogenetic for superfamilies, families, and subfamilies, starting with the accepted most basal-grade taxa, and is alphabetic for supertribes, tribes, and subtribes. Genera and species are listed alphabetically. Synonyms proposed after the publication of Bousquet et al. (2013) are included in this checklist (species name indented). The species included in this checklist list are based on New Brunswick records contained in Bousquet et al. (2013) and records published after that checklist.

The authors of all scientific names are listed along with the date of publication of the taxa. To avoid confusion with authors with same last name, initials are included for the following authors: W.J. Brown, H. Clark, J.E. LeConte, K.B. Miller, W.V. Miller, O.F. Müller, P.W.J. Müller, C.R. Sahlberg, J.R. Sahlberg, C. Schaeffer, J.B. Smith, C.G. Thomson, J. Thomson, C.C. Zimmermann, and R.E. White. LeConte and Horn stand for J.L. LeConte and G. Horn, respectively.

An asterisk [*] after a species name indicates that the taxon is Holarctic, a dagger [†] denotes an adventive species in North America, a double dagger [‡] indicates that its status is uncertain, and the species could be Holarctic or adventive in North America. Species in **bold** are newly recorded in this special issue, species in **bold** with a **^§^** are species newly described in this special issue.

**Table 1.** Total number of Coleoptera species by family and number of adventive, Holarctic, and status undetermined (Holarctic/adventive) in New Brunswick, Canada.

	No. of species	Adventive species	Holarctic species	Holarctic or Adventive
**Adephaga**				
Gyrinidae	19			
Carabidae	337	31	30	
Haliplidae	14		1	
Dytiscidae	108		20	
**Polyphaga**				
**Hydrophiloidea**				
Helophoridae	11	1	2	
Hydrochidae	2			
Hydrophilidae	52	14	2	
Histeridae	45	6		
**Staphylinoidea**				
Hydraenidae	3			
Ptiliidae	16	7	2	
Leiodidae	44	1	2	
Silphidae	11		3	
Staphylinidae	767	91	53	1
**Scarabaeoidea**				
Geotrupidae	4	1		
Trogidae	4			
Lucanidae	4			
Scarabaeidae	55	14	1	
**Scirtoidea**				
Eucinetidae	5	1		
Clambidae	2	1		
Scirtidae	15	1	1	
**Buprestoidea**				
Buprestidae	59	1	1	
**Byrrhoidea**				
Byrrhidae	7	1	1	
Elmidae	13			
Dryopidae	3	1		
Limnichidae	1			
Heteroceridae	6			
Psephenidae	3			
Ptilodactylidae	2			
**Elateroidea**				
Artematopodidae	1			
Eucnemidae	19	1		
Throscidae	3			
Elateridae	132	3	3	1
Lycidae	16		1	
Lampyridae	12			
Cantharidae	58	3	1	
**Derodontoidea**				
Derodontidae	2	1		
**Bostrichoidea**				
Dermestidae	15	8		
Endecatomidae	1			
Bostrichidae	6	3	1	
Ptinidae	44	15		
**Lymexyloidea**				
Lymexylidae	1			
**Cleroidea**				
Trogossitidae	7	1		1
Cleridae	19	1		
Melyridae	9	1		
**Cucujoidea**				
Byturidae	1			
Sphinididae	4			
Biphyllidae	1			
Erotylidae	10			
Monotomidae	11	3		
Cryptophagidae	31	12	5	1
Silvanidae	9	5		
Cucujidae	2		1	
Phalacridae	4			
Laemophloeidae	7	2		
Kateretidae	5	2	1	
Nitidulidae	52	7	5	1
Cerylonidae	3			
Endomychidae	8	1		
Coccinellidae	45	4	1	1
Corylophidae	9	1		
Latridiidae	27	13	4	
**Tenebrionoidea**				
Mycetophagidae	8	1		1
Ciidae	17	1	4	
Tetratomidae	12	1		
Melandryidae	28		2	
Mordellidae	32			
Ripiphoridae	2			
Zopheridae	4	1		
Tenebrionidae	44	6	1	2
Synchroidae	1			
Stenotrachelidae	8	1		
Meloidae	7			
Mycteridae	1			
Boridae	2			
Pythidae	5			
Pyrochroidae	8			
Salpingidae	2			
Anthicidae	16	1		
Aderidae	6	1		
Scraptiidae	5			
Ischaliidae	1			
**Chrysomeloidea**				
Cerambycidae	138	4	3	
Megalopodidae	4	1		
Orsodacnidae	1			
Chrysomelidae	205	31	3	
**Curculionoidea**				
Nemonychidae	3			
Anthribidae	14		1	
Attelabidae	4			
Brentidae	14	3		
Dryophthoridae	9	2		
Brachyceridae	8	1	3	
Curculionidae	267	52	8	1
**Total**	**3062**	**366**	**166**	**10**

### Order COLEOPTERA

**Suborder ADEPHAGA**

**Family GYRINIDAE Latreille, 1810**

(Whirligig beetles)

**Subfamily GYRININAE Latreille, 1810**

**Tribe Enhydrusini Régimbart, 1882**

Subtribe Dineutina Desmarest, 1851

*Dineutus assimilis* (Kirby, 1837)

*Dineutus discolor* Aubé, 1838

*Dineutus hornii* Roberts, 1895

*Dineutus nigrior* Roberts, 1895

**Tribe Gyrinini Latreille, 1810**

Subtribe Gyrinina Latreille, 1810

Gyrinus (Gyrinus) aeneolus LeConte, 1868

Gyrinus (Gyrinus) affinis Aubé, 1838

Gyrinus (Gyrinus) aquiris LeConte, 1868

Gyrinus (Gyrinus) bifarius Fall, 1922

Gyrinus (Gyrinus) confinis LeConte, 1868

Gyrinus (Gyrinus) fraternus Couper, 1865

Gyrinus (Gyrinus) gehringi Chamberlain, 1929

Gyrinus (Gyrinus) gibber LeConte, 1868

Gyrinus (Gyrinus) latilimbus Fall, 1922

Gyrinus (Gyrinus) lecontei Fall, 1922

**Gyrinus (Gyrinus) marginellus Fall, 1922**

Gyrinus (Gyrinus) pectoralis LeConte, 1868

Gyrinus (Gyrinus) pugionis Fall, 1922

Gyrinus (Gyrinus) sayi Aubé, 1838

**Gyrinus (Gyrinus) ventralis Kirby, 1837**

**Family CARABIDAE Latreille, 1810**

(Ground beetles)

**Subfamily NEBRIINAE Laporte, 1834**

**Tribe Nebriini Laporte, 1834**

Nebria (Boreonebria) lacustris Casey, 1913

Nebria (Reductonebria) pallipes Say, 1823

**Tribe Notiophilini Motschulsky, 1850**

*Notiophilus aeneus* (Herbst, 1806)

*Notiophilus aquaticus* (Linnaeus, 1758)*

*Notiophilus biguttatus* (Fabricius, 1779)†

*Notiophilus semistriatus* Say, 1823

**Subfamily CICINDELINAE Latreille, 1802**

**Tribe Cicindelini Latreille, 1802**

Subtribe Cicindelina Latreille, 1802

Cicindela (Cicindela) ancocisconensis Harris, 1852

Cicindela (Cicindela) duodecimguttata Dejean, 1825

Cicindela (Cicindela) hirticollis rhodensis Calder, 1916

Cicindela (Cicindela) limbalis Klug, 1834

Cicindela (Cicindela) longilabris longilabris Say, 1824

Cicindela (Cicindela) repanda repanda Dejean, 1825

Cicindela (Cicindela) sexguttata Fabricius, 1775

Cicindela (Cicindela) tranquebarica tranquebarica Herbst, 1806

Cicindela (Cicindelidia) marginipennis Dejean, 1831

Cicindela (Cicindelidia) punctulata Olivier, 1790

**Subfamily CARABINAE Latreille, 1802**

**Tribe Carabini Latreille, 1802**

Calosoma (Calodrepa) scrutator (Fabricius, 1775)

Calosoma (Calosoma) frigidum Kirby, 1837

Calosoma (Chrysostigma) calidum (Fabricius, 1775)

Carabus (Archicarabus) nemoralis nemoralis O. F. Müller, 1764†

Carabus (Carabus) granulatus granulatus Linnaeus, 1758†

Carabus (Hemicarabus) serratus Say, 1823

Carabus (Homoeocarabus) maeander maeander Fischer von Waldheim, 1820

Carabus (Tachypus) auratus auratus Linnaeus, 1761†

**Tribe Cychrini Perty, 1830**

Scaphinotus (Irichroa) viduus (Dejean, 1826)

Scaphinotus (Nomaretus) bilobus (Say, 1823)

*Sphaeroderus canadensis candensis* Chaudoir, 1861

*Sphaeroderus nitidicollis* Guérin-Méneville, 1829

*Sphaeroderus stenostomus lecontei* Dejean, 1826

**Subfamily LORICERINAE Bonelli, 1810**

**Tribe Loricerini Bonelli, 1810**

Loricera (Loricera) pilicornis pilicornis (Fabricius, 1775)*

**Subfamily OMOPHRONINAE Bonelli, 1810**

**Tribe Omophronini Bonelli, 1810**

*Omophron americanum* Dejean, 1831

*Omophron tessellatum* Say, 1823

**Subfamily ELAPHRINAE Latreille, 1802**

**Tribe Elaphrini Latreille, 1802**

*Blethisa hudsonica* Casey, 1924

*Blethisa julii* LeConte, 1863

*Blethisa quadricollis* Haldeman, 1847

Elaphrus (Elaphrus) americanus americanus Dejean, 1831

Elaphrus (Elaphrus) californicus Mannerheim, 1843

Elaphrus (Neoelaphrus) clairvillei Kirby, 1837

Elaphrus (Neoelaphrus) olivaceus LeConte, 1863

**Subfamily SCARITINAE Bonelli, 1810**

**Tribe Clivinini Rafinesque, 1815**

Subtribe Clivinina Rafinesque, 1815

Clivina (Clivina) fossor fossor (Linnaeus, 1758)†

Clivina (Leucocara) americana Dejean, 1831

Schizogenius (Schizogenius) lineolatus (Say, 1823)

Schizogenius (Schizogenius) sulcifrons Putzeys, 1846

**Tribe Dyschiriini Kolbe, 1880**

*Dyschirius dejeanii* Putzeys, 1846

*Dyschirius erythrocerus* LeConte, 1857

*Dyschirius globulosus* (Say, 1823)

*Dyschirius larochellei* Bousquet, 1988

*Dyschirius pilosus* LeConte, 1857

*Dyschirius politus politus* (Dejean, 1825)*

*Dyschirius sellatus* LeConte, 1857

*Dyschirius setosus* LeConte, 1857

*Dyschirius sphaericollis* (Say, 1823)

**Subfamily BROSCINAE Hope, 1838**

**Tribe Broscini Hope, 1838**

Subtribe Broscina Hope, 1838

*Miscodera arctica* (Paykull, 1798)*

**Subfamily TRECHINAE Bonelli, 1810**

**Tribe Bembidiini Stephens, 1827**

Subtribe Bembidiina Stephens, 1827

*Amerizus wingatei* (Bland, 1864)

Bembidion (Asioperyphus) postremum Say, 1830

Bembidion (Bembidion) mutatum Gemminger & Harold, 1868

Bembidion (Bembidion) quadrimaculatum oppositum Say, 1823

Bembidion (Bracteon) carinula Chaudoir, 1868

Bembidion (Bracteon) inaequale Say, 1823

Bembidion (Bracteon) levettei carrianum Casey, 1924

Bembidion (Bracteon) punctatostriatum Say, 1823

Bembidion (Diplocampa) transparens transparens (Gebler, 1830)*

Bembidion (Eupetedromus) graciliforme Hayward, 1897

Bembidion (Eupetedromus) immaturum Lindroth, 1954

Bembidion (Eupetedromus) incrematum LeConte, 1860*

Bembidion (Eupetedromus) iridipenne Bousquet & Webster, 2006

Bembidion (Eupetedromus) variegatum Say, 1823

Bembidion (Furcacampa) impotens Casey, 1918

Bembidion (Furcacampa) mimus Hayward, 1897

Bembidion (Furcacampa) versicolor (LeConte, 1847)

Bembidion (Hirmoplataphus) concolor (Kirby, 1837)

Bembidion (Hirmoplataphus) nigrum Say, 1823

Bembidion (Hirmoplataphus) salebratum (LeConte, 1847)

Bembidion (Hydriomicrus) quadratulum Notman, 1920

Bembidion (Hydriomicrus) semistriatum (Haldeman, 1843)

Bembidion (Hydrium) nitidum (Kirby, 1837)

Bembidion (Melomalus) planatum (LeConte, 1847)

Bembidion (Metallina) properans (Stephens, 1828)†

Bembidion (Notaphus) castor Lindroth, 1963

Bembidion (Notaphus) constrictum (LeConte, 1847)

Bembidion (Notaphus) contractum Say, 1823

Bembidion (Notaphus) nigripes (Kirby, 1837)*

Bembidion (Notaphus) oberthueri Hayward, 1901

Bembidion (Notaphus) patruele Dejean, 1831

Bembidion (Notaphus) rapidum (LeConte, 1847)

Bembidion (Notaphus) versutum LeConte, 1878

Bembidion (Ochthedromus) americanum Dejean, 1831

Bembidion (Ocydromus) scopulinum (Kirby, 1837)*

Bembidion (Odontium) confusum Hayward, 1897

Bembidion (Peryphanes) grapii Gyllenhal, 1827*

Bembidion (Peryphanes) lacunarium (C.C.A. Zimmermann, 1869)

Bembidion (Peryphanes) stephensii Crotch, 1866†

Bembidion (Peryphus) bruxellense Wesmael, 1835†

Bembidion (Peryphus) femoratum femoratum Sturm, 1825†

Bembidion (Peryphus) obcurellum obscurellum (Motschulsky, 1845)*

Bembidion (Peryphus) petrosum petrosum Gebler, 1833*

Bembidion (Peryphus) sejunctum sejunctum Casey, 1918

Bembidion (Peryphus) tetracolum tetracolum Say, 1823†

Bembidion (Peryphus) transversale Dejean, 1831

Bembidion (Plataphus) carolinense Casey, 1924

Bembidion (Plataphus) occultator Notman, 1920

Bembidion (Plataphus) rusticum rusticum Casey, 1918

Bembidion (Plataphus) sulcipenne prasinoides Lindroth, 1963

Bembidion (Pseudoperyphus) antiquum Dejean, 1831

Bembidion (Pseudoperyphus) chalceum Dejean, 1831

Bembidion (Pseudoperyphus) honestum Say, 1823

Bembidion (Pseudoperyphus) louisella Maddison, 2008

Bembidion (Pseudoperyphus) rothfelsi Maddison, 2008

Bembidion (Pseudoperyphus) rufotinctum Chaudoir, 1868

Bembidion (Semicampa) muscicola Hayward, 1897

Bembidion (Semicampa) nigrivestris Bousquet, 2006

Bembidion (Semicampa) praticola Lindroth, 1963

Bembidion (Semicampa) semicinctum Notman, 1919

Bembidion (Trepanedoris) concretum Casey, 1918

Bembidion (Trepanedoris) fortestriatum (Motschulsky, 1845)

Bembidion (Trepanedoris) frontale (LeConte, 1847)

Bembidion (Trepanedoris) pseudocautum Lindroth, 1963

Bembidion (Trichoplataphus) planum (Haldeman, 1843)

Bembidion (Trichoplataphus) rolandi Fall, 1922

Subtribe Tachyina Motschulsky, 1862

Elaphropus (Barytachys) anceps (LeConte, 1848)

Elaphropus (Barytachys) granarius (Dejean, 1831)

Elaphropus (Barytachys) incurvus (Say, 1830)

Elaphropus (Barytachys) saturatus (Casey, 1918)

Elaphropus (Barytachys) tripunctatus (Say, 1830)

Elaphropus (Barytachys) vernicatus (Casey, 1918)

*Polyderis laeva* (Say, 1823)

*Porotachys bisulcatus* (Nicolai, 1822)†

Tachys (Paratachys) rhodeanus Casey, 1918

Tachys (Paratachys) scitulus LeConte, 1848

Tachyta (Tachyta) angulata Casey, 1918

Subtribe Xystosomina Erwin, 1994

*Mioptachys flavicauda* (Say, 1823)

**Tribe Trechini Bonelli, 1810**

*Blemus discus discus* (Fabricius, 1792)†

Trechus (Trechus) apicalis Motschulski, 1845*

Trechus (Trechus) crassiscapus Lindroth, 1955

Trechus (Trechus) rubens (Fabricius, 1792)†

**Subfamily PATROBINAE Kirby, 1837**

**Tribe Patrobini Kirby, 1837**

Subtribe Deltomerina Chaudoir, 1871

Diplous (Platydius) rugicollis (Randall, 1838)

Subtribe Patrobina Kirby, 1837

*Patrobus foveocollis* (Eschscholtz, 1823)*

*Patrobus lecontei* Chaudoir, 1872

*Patrobus longicornis* (Say, 1823)

*Patrobus septentrionis septentrionis* Dejean, 1828*

*Platypatrobus lacustris* Darlington, 1838

**Subfamily BRACHININAE Bonelli, 1810**

**Tribe Brachinini Bonelli, 1810**

Subtribe Brachinina Bonelli, 1810

Brachinus (Neobrachinus) cordicollis Dejean, 1826

Brachinus (Neobrachinus) cyanipennis Say, 1823

Brachinus (Neobrachinus) cyanochroaticus Erwin, 1969

Brachinus (Neobrachinus) fumans (Fabricius, 1781)

**Subfamily HARPALINAE Bonelli, 1810**

**Tribe Atranini Horn, 1881**

*Atranus pubescens* (Dejean, 1828)

**Tribe Chlaeniini Brullé, 1834**

Chlaenius (Agostenus) alternatus Horn, 1871

Chlaenius (Agostenus) niger Randall, 1838

Chlaenius (Anomoglossus) emarginatus Say, 1823

Chlaenius (Brachylobus) lithophilus Say, 1823

Chlaenius (Chlaeniellus) impunctifrons Say, 1823

Chlaenius (Chlaeniellus) pensylvanicus pensylvanicus Say, 1823

Chlaenius (Chlaeniellus) tricolor tricolor Dejean, 1826

Chlaenius (Chlaenius) sericeus (Forster, 1771)

Chlaenius (Lithochlaenius) cordicollis Kirby, 1837

**Tribe Harpalini Bonelli, 1810**

Subtribe Anisodactylina Lacordaire, 1854

Amphasia (Pseudamphasia) sericea (Harris, 1828)

Anisodactylus (Anadaptus) discoideus Dejean, 1831

Anisodactylus (Anadaptus) sanctaecrucis (Fabricius, 1798)

Anisodactylus (Anisodactylus) harrisii LeConte, 1863

Anisodactylus (Anisodactylus) kirbyi Lindroth, 1953

Anisodactylus (Anisodactylus) nigerrimus (Dejean, 1831)

Anisodactylus (Anisodactylus) nigrita Dejean, 1829

Anisodactylus (Gynandrotarsus) rusticus (Say, 1823)

Anisodactylus (Spongopus) verticalis (LeConte, 1847)

Notiobia (Anisotarsus) terminata (Say, 1823)

*Xestonotus lugubris* (Dejean, 1829)

Subtribe Harpalina Bonelli, 1810

Harpalus (Harpalus) affinis (Schrank, 1781)†

Harpalus (Harpalus) herbivagus Say, 1823

Harpalus (Harpalus) opacipennis (Haldeman, 1843)

Harpalus (Harpalus) plenalis Casey, 1914

Harpalus (Harpalus) rubripes (Duftschmid, 1812)†

Harpalus (Harpalus) solitaris Dejean, 1829*

Harpalus (Harpalus) somnulentus Dejean, 1829

Harpalus (Opadius) fulvilabris Mannerheim, 1853

Harpalus (Opadius) indigens Casey, 1924

Harpalus (Opadius) laevipes Zetterstedt, 1828*

Harpalus (Opadius) laticeps LeConte, 1850

Harpalus (Opadius) lewisii LeConte, 1865

Harpalus (Opadius) megacephalus LeConte, 1847

Harpalus (Opadius) nigritarsis C.R. Sahlberg, 1827*

Harpalus (Pseudophonus) pensylvanicus (DeGeer, 1774)

Harpalus (Pseudophonus) rufipes (DeGeer, 1774)†

*Ophonus puncticeps* Stephens, 1828†

Selenophorus (Selenophorus) gagatinus Dejean, 1829

Selenophorus (Selenophorus) opalinus (LeConte, 1863)

Trichotichnus (Trichotichnus) vulpeculus (Say, 1823)

Subtribe Stenolophina Kirby, 1837

Acupalpus (Acupalpus) canadensis Casey, 1924

Acupalpus (Acupalpus) carus (LeConte, 1863)

Acupalpus (Acupalpus) nanellus Casey, 1914

Acupalpus (Tachistodes) pauperculus Dejean, 1829

*Agonoleptus conjunctus* (Say, 1823)

Bradycellus (Catharellus) lecontei Csiki, 1932

Bradycellus (Lipalocellus) nigrinus (Dejean, 1829)

Bradycellus (Lipalocellus) semipubescens Lindroth, 1968

Bradycellus (Stenocellus) neglectus (LeConte, 1847)

Bradycellus (Stenocellus) nigriceps LeConte, 1869

Bradycellus (Stenocellus) rupestris (Say, 1823)

Bradycellus (Triliarthrus) atrimedeus (Say, 1823)

Bradycellus (Triliarthrus) badipennis (Haldeman, 1843)

Bradycellus (Triliarthrus) kirbyi (Horn, 1883)

Bradycellus (Triliarthrus) lugubris (LeConte, 1847)

Dicheirotrichus (Trichocellus) cognatus (Gyllenhal, 1827)*

Stenolophus (Agonoderus) comma (Fabricius, 1775)

Stenolophus (Stenolophus) fuliginosus Dejean, 1829

Stenolophus (Stenolophus) fuscatus Dejean, 1829

Stenolophus (Stenolophus) ochropezus (Say, 1823)

**Tribe Lebiini Bonelli, 1810**

Subtribe Cymindidina Laporte, 1834

Cymindus (Pinacodera) limbata Dejean, 1831

Cymindus (Tarulus) borealis LeConte, 1863

Cymindus (Tarulus) cribricollis Dejean, 1831

Cymindus (Tarulus) neglecta Haldeman, 1843

Subtribe Dromiusina Bonelli, 1810

*Apristus latens* (LeConte, 1846)

*Apristus subsulcatus* (Dejean, 1826)

*Dromius piceus* Dejean, 1831

*Syntomus americanus* (Dejean, 1831)

Subtribe Lebiina Bonelli, 1810

Lebia (Lebia) fuscata Dejean, 1825

Lebia (Lebia) moesta LeConte, 1850

Lebia (Lebia) ornata Say, 1823

Lebia (Lebia) pumila Dejean, 1831

**Lebia (Lebia) solea Hentz, 1830**

Lebia (Lebia) viridis Say, 1823

Lebia (Loxopeza) tricolor Say, 1823

**Tribe Licinini Bonelli, 1810**

Subtribe Dicaelina Laporte, 1834

Diplocheila (Isorembus) assimilis (LeConte, 1844)

Diplocheila (Isorembus) obtusa (LeConte, 1847)

Diplocheila (Isorembus) striatopunctata (LeConte, 1844)

Subtribe Licinina Bonelli, 1810

Badister (Badister) neopulchellus Lindroth, 1954

Badister (Badister) notatus Haldeman, 1843

Badister (Badister) obtusus LeConte, 1878

Badister (Baudia) grandiceps Casey, 1920

Badister (Baudia) micans LeConte, 1844

Badister (Baudia) reflexus LeConte, 1880

Badister (Baudia) transversus Casey, 1920

**Tribe Oodini Laferté-Sénectère, 1851**

*Lachnocrepis parallela* (Say, 1830)

***Oodes fluvialis* LeConte, 1863**

**Tribe Perigonini Horn, 1881**

Perigonia (Trechicus) nigriceps (Dejean, 1831)†

**Tribe Platynini Bonelli, 1810**

Agonum (Agonum) muelleri (Herbst, 1784)†

Agonum (Agonum) piceolum (LeConte, 1879)

Agonum (Agonum) placidum (Say, 1823)

Agonum (Europhilus) anchomenoides Randall, 1838

Agonum (Europhilus) canadense Goulet, 1969

Agonum (Europhilus) consimile (Gyllenhal, 1810)*

Agonum (Europhilus) darlingtoni Lindroth, 1954

Agonum (Europhilus) gratiosum (Mannerheim, 1853)*

Agonum (Europhilus) lutulentum (LeConte, 1854)

Agonum (Europhilus) palustre Goulet, 1969

Agonum (Europhilus) picicornoides Lindroth, 1966

Agonum (Europhilus) retractum LeConte, 1846

Agonum (Europhilus) sordens Kirby, 1837

Agonum (Europhilus) superioris Lindroth, 1966

Agonum (Europhilus) thoreyi Dejean, 1828*

Agonum (Olisares) aeruginosum Dejean, 1828

Agonum (Olisares) affine Kirby, 1837

Agonum (Olisares) crenistriatum (LeConte, 1863)

Agonum (Olisares) cupreum Dejean, 1831

Agonum (Olisares) cupripenne (Say, 1823)

Agonum (Olisares) excavatum Dejean, 1828

Agonum (Olisares) extensicolle (Say, 1823)

Agonum (Olisares) fidele Casey, 1920

Agonum (Olisares) harrisii LeConte, 1846

Agonum (Olisares) melanarium Dejean, 1828

Agonum (Olisares) metallescens (LeConte, 1854)

Agonum (Olisares) mutatum (Gemminger & Harold, 1868)

Agonum (Olisares) octopunctatum (Fabricius, 1798)

Agonum (Olisares) propinquum (Gemminger & Harold, 1868)

Agonum (Olisares) tenue (LeConte, 1854)

Agonum (Olisares) trigeminum Lindroth, 1954

Agonum (Platynomicrus) nigriceps LeConte, 1846*

*Olisthopus parmatus* (Say, 1823)

*Oxypselaphus pusillus* (LeConte, 1854)

*Paranchus albipes* (Fabricius, 1794)†

**Platynus (Batenus) cincticollis (Say, 1823)**

Platynus (Batenus) mannerheimii (Dejean, 1828)*

Platynus (Platynus) decentis (Say, 1823)

Platynus (Platynus) indecentis Liebherr & Will, 1996

Platynus (Platynus) opaculus LeConte, 1863

Platynus (Platynus) tenuicollis (LeConte, 1846)

*Sericoda obsoleta* (Say, 1823)

*Sericoda quadripuncatata* (DeGeer, 1774)*

**Tribe Pterostichini Bonelli, 1810**

Subtribe Pterostichina Bonelli, 1810

*Gastrellarius honestus* (Say, 1823)

Myas (Trigonognatha) cyanescens Dejean, 1828

Poecilus (Poecilus) chalcites (Say, 1823)

Poecilus (Poecilus) lucublandus (Say, 1823)

Pterostichus (Argutor) commutabilis (Motschulsky, 1866)

Pterostichus (Bothriopterus) adstrictus Eschscholtz, 1823*

Pterostichus (Bothriopterus) mutus (Say, 1823)

Pterostichus (Bothriopterus) pensylvanicus LeConte, 1873

Pterostichus (Cylindrocharis) rostratus (Newman, 1838)

Pterostichus (Euferonia) coracinus (Newman, 1838)

Pterostichus (Euferonia) lachrymosus (Newman, 1838)

Pterostichus (Hypherpes) adoxus (Say, 1823)

Pterostichus (Hypherpes) tristis (Dejean, 1828)

Pterostichus (Lenapterus) punctatissimus (Randall, 1838)

Pterostichus (Melanius) castor Goulet & Bousquet, 1983

Pterostichus (Melanius) corvinus (Dejean, 1828)

Pterostichus (Morphnosoma) melanarius melanarius (Illiger, 1798)†

Pterostichus (Phonias) corrusculus LeConte, 1873

Pterostichus (Phonias) femoralis (Kirby, 1837)

Pterostichus (Phonias) patruelis (Dejean, 1831)

Pterostichus (Pseudomaseus) luctuosus (Dejean, 1828)

Pterostichus (Pseudomaseus) tenuis (Casey, 1924)

Stomis (Stomis) pumicatus (Panzer, 1795)†

**Tribe Sphodrini Laporte, 1834**

Subtribe Atranopsina Baehr, 1982

*Pseudamara arenaria* (LeConte, 1847)

Subtribe Calathina Laporte, 1834

Calathus (Acalathus) advena (LeConte, 1846)

Calathus (Neocalathus) gregarius (Say, 1823)

Calathus (Neocalathus) ingratus Dejean, 1828

Subtribe Sphodrina Laporte, 1834

Laemostenus (Pristonychus) terricola terricola (Herbst, 1784)†

Subtribe Synuchina Lindroth, 1956

*Synuchus impunctatus* (Say, 1823)

**Tribe Zabrini Bonelli, 1810**

Subtribe Amarina C.C.A. Zimmermann, 1832

Amara (Amara) aenea (DeGeer, 1774)†

Amara (Amara) aeneopolita Casey, 1918

Amara (Amara) communis (Panzer, 1797)†

Amara (Amara) cupreolata Putzeys, 1866

Amara (Amara) familiaris (Duftschmid, 1812)†

Amara (Amara) littoralis Dejean, 1828*

Amara (Amara) lunicollis Schiodte, 1837*

Amara (Amara) neoscotica Casey, 1924

Amara (Amara) otiosa Casey, 1918

Amara (Amara) ovata (Fabricius, 1792)†

Amara (Amarocelia) ellipsis (Casey, 1918)

Amara (Amarocelia) erratica (Duftschmid, 1812)*

Amara (Amarocelia) laevipennis Kirby, 1837

Amara (Amarocelia) patruelis Dejean, 1831*

Amara (Bradytus) apricaria (Paykull, 1790)†

Amara (Bradytus) avida (Say, 1823)

Amara (Bradytus) fulva (O.F. Müller, 1776)†

Amara (Bradytus) latior (Kirby, 1837)

Amara (Celia) bifrons (Gyllenhal, 1810)†

Amara (Celia) musculus (Say, 1823)

Amara (Celia) rubrica Haldeman, 1843

Amara (Celia) sinuosa (Casey, 1918)

Amara (Curtonotus) aulica (Panzer, 1796)†

Amara (Curtonotus) lacustris LeConte, 1855

Amara (Curtonotus) torrida (Panzer, 1796)*

Amara (Paracelia) quenseli quenseli (Schönherr, 1806)*

Amara (Percosia) obesa (Say, 1823)

Amara (Xenocelia) gibba (LeConte, 1847)

Amara (Zezea) angustatoides Hieke, 2000

Amara (Zezea) flebilis (Casey, 1918)

Amara (Zezea) pallipes Kirby, 1837

**Family HALIPLIDAE Aubé, 1836**

(Crawling water beetles)

Haliplus (Haliplus) immaculicollis Harris, 1828

Haliplus (Haliplus) longulus LeConte, 1850

Haliplus (Liaphlus) canadensis Wallis, 1933

Haliplus (Liaphlus) connexus Matheson, 1912

Haliplus (Liaphlus) cribrarius LeConte, 1850

Haliplus (Liaphlus) fulvus (Fabricius, 1801)*

Haliplus (Paraliaphlus) borealis LeConte, 1850

Haliplus (Paraliaphlus) leopardus Roberts, 1913

Haliplus (Paraliaphlus) pantherinus Aubé, 1823

Peltodytes (Neopeltodytes) duodecimpunctatus (Say, 1823)

Peltodytes (Neopeltodytes) edentulus (LeConte, 1863)

Peltodytes (Neopeltodytes) lengi Roberts, 1913

Peltodytes (Neopeltodytes) litoralis Matheson, 1912

Peltodytes (Neopeltodytes) tortulosus Roberts, 1913

**Family DYTISCIDAE Leach, 1815**

(Predaceous diving beetles)

**Subfamily COPELATINAE Branden, 1885**

***Copelatus glyphicus* (Say, 1823)**

**Subfamily LACCOPHILINAE Gistel, 1848**

**Tribe Laccophilini Gistel, 1848**

*Laccophilus biguttatus* Kirby, 1837*

*Laccophilus maculosus maculosus* Say, 1823

**Subfamily HYDROPORINAE Aubé, 1836**

**Tribe Bidessini Sharp, 1880**

*Liodessus affinis* (Say, 1823)

*Liodessus crotchi* Nilsson, 2001

*Liodessus noviaffinis* K.B. Miller, 1998

*Uvarus falli* (Young, 1940)

*Uvarus granarius* (Aubé, 1838)

**Tribe Hydroporini Aubé, 1836**

*Boreonectes griseostriatus* (DeGeer, 1774)

*Heterosternuta allegheniana* (Matta & Wolfe, 1979)

*Heterosternuta cocheconis* (Fall, 1917)

*Heterosternuta pulchra* (LeConte, 1855)

*Hydrocolus filiolus* (Fall, 1923)

*Hydrocolus paugus* (Fall, 1923)

*Hydrocolus persimilis* (Crotch, 1873)

*Hydrocolus rubyae* (Larson, 1975)

*Hydrocolus rufiplanulus* (Fall, 1923)

*Hydrocolus stagnalis* (Gemminger & Harold, 1868)

*Hydroporus badiellus* Fall, 1923

*Hydroporus brevicornis* Fall, 1917

*Hydroporus columbianus* Fall, 1923

*Hydroporus dentellus* Fall, 1917

*Hydroporus fuscipennis* Schaum, 1868*

*Hydroporus gossei* Larson & Roughley, 2000

*Hydroporus nigellus* Mannerheim, 1853*

*Hydroporus niger* Say, 1823

*Hydroporus notabilis* LeConte, 1850*

*Hydroporus obscurus* Sturm, 1835*

*Hydroporus puberulus* LeConte, 1850*

*Hydroporus rectus* Fall, 1923

*Hydroporus rufinasus* Mannerheim, 1852

*Hydroporus signatus signatus* Mannerheim, 1853

*Hydroporus striola* (Gyllenhal, 1826)*

*Hydroporus tenebrosus* LeConte, 1850

*Hydroporus tristis* (Paykull, 1798)*

*Nebrioporus rotundatus* (LeConte, 1863)

*Neoporus carolinus* (Fall, 1917)

*Neoporus clypealis* (Sharp, 1882)

*Neoporus dilatatus* (Fall, 1917)

*Neoporus dimidiatus* (Gemminger & Harold, 1868)

*Neoporus mellitus* (LeConte, 1855)

*Neoporus spurius* (LeConte, 1855)

*Neoporus sulcipennis* (Fall, 1917)

*Neoporus undulatus* (Say, 1823)

*Oreodytes scitulus scitulus* (LeConte, 1855)

*Sanfilippodytes pseudovilis* (Young, 1953)

**Tribe Hygrotini Portevin, 1929**

*Coelambus compar* Fall, 1919

*Coelambus falli* Wallis, 1924

*Coelambus impressopunctatus* (Schaller, 1783)*

*Coelambus laccophilinus* (LeConte, 1878)

*Coelambus nubilis* (LeConte, 1855)

*Coelambus patruelis* (LeConte, 1855)

*Coelambus picatus* (Kirby, 1837)

*Coelambus turbidus* (LeConte, 1855)

*Hygrotus farctus* (LeConte, 1855)

*Hygrotus sayi* Balfour-Browne, 1944

**Tribe Hyphydrini Gistel, 1848**

*Desmopachria convexa* (Aubé, 1838)

**Tribe Laccornini Wolfe & Roughley, 1990**

*Laccornis conoideus* (LeConte, 1850)

*Laccornis latens* (Fall, 1937)

**Subfamily AGABINAE C.G. Thomson, 1867**

Agabus (Acatodes) anthracinus Mannerheim, 1852

**Agabus (Acatodes) bicolor (Kirby, 1837)**

Agabus (Acatodes) confinis (Gyllenhal, 1808)

Agabus (Acatodes) discolor (Harris, 1828)*

Agabus (Acatodes) immaturus Larson, 1991

Agabus (Acatodes) inscriptus (Crotch, 1873)

Agabus (Acatodes) phaeopterus (Kirby, 1837)

Agabus (Acatodes) subfuscatus Sharp, 1882

Agabus (Agabus) bifarius (Kirby, 1837)*

Agabus (Agabus) punctulatus Aubé, 1838

Agabus (Gaurodytes) ambiguus (Say, 1823)

Agabus (Gaurodytes) erythropterus (Say, 1823)

Agabus (Gaurodytes) leptapsis (LeConte, 1878)

Agabus (Gaurodytes) semipunctatus (Kirby, 1837)

*Ilybiosoma seriatum* (Say, 1823)

*Ilybius angustior* (Gyllenhal, 1808)*

*Ilybius biguttulus* (Germar, 1824)

*Ilybius confusus* Aubé, 1838

*Ilybius discedens* Sharp, 1882

*Ilybius erichsoni* (Gemminger & Harold, 1868)*

***Ilybius ignarus* (LeConte, 1862)**

*Ilybius larsoni* (Fery & Nilsson, 1993)

*Ilybius opacus* (Aubé, 1837)*

*Ilybius pleuriticus* LeConte, 1850

*Ilybius wasastjernae* (C.R. Sahlberg, 1824)*

*Platambus obtusatus* (Say, 1823)

**Subfamily COPTOTOMINAE Branden, 1885**

*Coptotomus longulus lenticus* Hilsenhoff, 1980

**Subfamily COLYMBETINAE Erichson, 1837**

**Tribe Colymbetini Erichson, 1837**

*Colymbetus paykulli* Erichson, 1837*

*Colymbetus sculptilis* Harris, 1829

*Neoscutopterus angustus* (LeConte, 1850)

*Neoscutopterus hornii* (Crotch, 1873)

Rhantus (Nartus) sinuatus (LeConte, 1862)

Rhantus (Rhantus) binotatus (Harris, 1828)

Rhantus (Rhantus) consimilis Motschulsky, 1859

Rhantus (Rhantus) suturellus (Harris, 1828)*

Rhantus (Rhantus) wallisi Hatch, 1953

**Subfamily DYTISCINAE Leach, 1815**

**Tribe Aciliini C.G. Thomson, 1867**

Acilius (Acilius) semisulcatus Aubé, 1838

Acilius (Acilius) sylvanus Hilsenhoff, 1975

Acilius (Homoeolytrus) mediatus (Say, 1823)

*Graphoderus fascicollis* (Harris, 1828)

*Graphoderus liberus* (Say, 1825)

*Graphoderus perplexus* Sharp, 1882*

**Tribe Dytiscini Leach, 1815**

*Dytiscus cordieri* Aubé, 1838

*Dytiscus dauricus* Gebler, 1832*

*Dytiscus fasciventris* Say, 1824

*Dytiscus harrisii* Kirby, 1837

*Dytiscus verticalis* Say, 1823

**Tribe Hydaticini Sharp, 1880**

Hydaticus (Hydaticus) aruspex H. Clark, 1864*

Hydaticus (Hydaticus) piceus LeConte, 1863

**Suborder POLYPHAGA**

**Superfamily HYDROPHILOIDEA Latreille, 1802**

**Family HELOPHORIDAE Leach, 1815**

Helophorus (Helophorus) grandis Illiger, 1798†

Helophorus (Kyphohelophorus) tuberculatus Gyllenhal, 1808*

Helophorus (Rhopalohelophorus) angusticollis d’Orchymont, 1945

Helophorus (Rhopalohelophorus) frosti Smetana, 1985

Helophorus (Rhopalohelophorus) lacustris LeConte, 1850

Helophorus (Rhopalohelophorus) lineatus Say, 1823

**Helophorus (Rhopalohelophorus) marginicollis Smetana, 1985**

Helophorus (Rhopalohelophorus) nitiduloides d’Orchymont, 1945

**Helophorus (Rhopalohelophorus) oblongus LeConte, 1850**

Helophorus (Rhopalohelophorus) orientalis Motschulsky, 1860*

Helophorus (Rhopalohelophorus) sempervarians Angus, 1970

**Family HYDROCHIDAE C.G. Thomson, 1859**

*Hydrochus squamifer* LeConte, 1844

***Hydrochus subcupreus* Randall, 1838**

**Family HYDROPHILIDAE Latreille, 1802**

(Water scavenger beetles)

**Subfamily HYDROPHILINAE Latreille, 1802**

**Tribe Berosini Mulsant, 1844**

**Berosus (Berosus) fraternus LeConte, 1855**

**Berosus (Berosus) peregrinus (Herbst, 1797)**

**Berosus (Berosus) sayi Hansen, 1999**

**Tribe Laccobiini Houlbert, 1922**

*Laccobius agilus* (Randall, 1838)

*Laccobius minutoides* d’Orchymont, 1942

*Laccobius reflexipenis* Cheary, 1971

*Laccobius spangleri* Cheary, 1971

***Paracymus despectus* (LeConte, 1863)**

*Paracymus subcupreus* (Say, 1825)

**Tribe Hydrobiusini Mulsant, 1844**

*Hydrobius fuscipes* (Linnaeus, 1758)*

*Hydrobius melaenus* (Germar, 1824)

*Sperchopsis tessellata* (Ziegler, 1844)

**Tribe Hydrophilini Latreille, 1802**

*Hydrochara obtusata* (Say, 1823)

*Tropisternus glaber* (Herbst, 1797)

*Tropisternus mixtus* (LeConte, 1855)

**Subfamily CHAETARTHRIINAE Bedel, 1881**

**Tribe Anacaenini Hansen, 1991**

*Anacaena lutescens* (Stephens, 1829)†

Crentis (Crentis) digesta (LeConte, 1855)

Crentis (Crentis) monticola (Horn, 1890)

**Tribe Chaetarthriini Bedel, 1881**

***Chaetarthria atra* (LeConte, 1863)**

**Subfamily ENOCHRINAE Short & Fikáček, 2013**

***Cymbiodyta acuminata* Fall, 1924**

***Cymbiodyta blanchardi* Horn, 1890**

***Cymbiodyta minima* Notman, 1919**

*Cymbiodyta semistriata* (C.C.A. Zimmermann, 1869)

*Cymbiodyta vindicata* Fall, 1924

**Enochrus (Lumetus) hamiltoni (Horn, 1890)**

Enochrus (Lumetus) reflexipennis (C.C.A. Zimmermann, 1869)

Enochrus (Methydrus) cinctus (Say, 1824)

**Enochrus (Methydrus) consors (LeConte, 1863)**

**Enochrus (Methydrus) consortus Green, 1946**

Enochrus (Methydrus) ochraceus (Melsheimer, 1844)

**Enochrus (Methydrus) pygmaeus nebulosus (Say, 1824)**

*Helocombus bifidus* (LeConte, 1855)

**Subfamily SPHAERIDIINAE Latreille, 1802**

**Tribe Megasternini Mulsant, 1844**

Cercyon (Cercyon) assecla Smetana, 1978

**Cercyon (Cercyon) cinctus Smetana, 1978**

Cercyon (Cercyon) haemorrhoidalis (Fabricius, 1775)†

**Cercyon (Cercyon) herceus frigidus Smetana, 1978**

Cercyon (Cercyon) indistinctus Horn, 1890

Cercyon (Cercyon) lateralis (Marsham, 1802)†

Cercyon (Cercyon) littoralis (Gyllenhal, 1808)*

Cercyon (Cercyon) pygmaeus (Illiger, 1801)†

Cercyon (Cercyon) quisquilius (Linnaeus, 1760)†

Cercyon (Cercyon) terminatus (Marsham, 1802)†

Cercyon (Cercyon) unipunctatus (Linnaeus, 1758)†

**Cercyon (Dicyrtocercyon) ustulatus (Preyssler, 1790)**†

Cercyon (Paracercyon) analis (Paykull, 1798)†

Cercyon (Paracercyon) minusculus Melsheimer, 1844

Cercyon (Prostercyon) roseni Knisch, 1922

*Cryptopleurum minutum* (Fabricius, 1775)†

*Cryptopleurum subtile* Sharp, 1884†

**Tribe Sphaeridiini Latreille, 1802**

*Sphaeridium bipustulatum* Fabricius, 1781†

*Sphaeridium lunatum* Fabricius, 1792†

*Sphaeridium scarabaeoides* (Linnaeus, 1758)†

**Family HISTERIDAE Gyllenhal, 1808**

(Hister beetles)

**Subfamily ABRAEINAE MacLeay, 1819**

**Tribe Acritini Wenzel, 1944**

*Acritus exiguus* (Erichson, 1834)

*Aeletes politus* (LeConte, 1853)

**Tribe Plegaderini Portevin, 1929**

*Plegaderus confusus* Bousquet & Laplante, 1999

*Plegaderus sayi* Marseul, 1856

**Tribe Teretriini Bickhardt, 1914**

***Teretrius latebricola* Lewis, 1901**

**Subfamily SAPRININAE Blanchard, 1845**

*Baeckmanniolus dimidiatipennis* (J.E. LeConte, 1824)

Euspilotus (Hesperosaprinus) assimilis (Paykull, 1811)

Euspilotus (Hesperosaprinus) rossi (Wenzel, 1939)

Geomysaprinus (Priscosaprinus) moniliatus (Casey, 1916)

*Gnathoncus barbatus* Bousquet & Laplante, 1999

*Gnathoncus communis* (Marseul, 1862)†

*Gnathoncus rotundatus* (Kugelann, 1792)†

*Hypocaccus bigener* (J.E. LeConte, 1844)

*Hypocaccus fitchi* (Marseul, 1862)

*Hypocaccus fraternus* (Say, 1825)

**Subfamily DENDROPHILINAE Reitter, 1909**

**Tribe Dendrophilini Reitter, 1909**

*Dendrophilus kiteleyi* Bousquet & Laplante, 1999

*Dendrophilus punctatus* (Herbst, 1792)†

**Tribe Paromalini Reitter, 1909**

*Carcinops pumilio* (Erichson, 1834)†

*Paromalus teres* LeConte, 1878

**Subfamily HISTERINAE Gyllenhal, 1808**

**Tribe Histerini Gyllenhal, 1808**

*Atholus bimaculatus* (Linnaeus, 1758)†

*Atholus perplexus* (LeConte, 1863)

*Atholus sedecimstriatus* (Say, 1825)

*Hister abbreviatus* Fabricius, 1775

*Hister curtatus* J.E. LeConte, 1844

*Hister furtivus* J.E. LeConte, 1859

Margarinotus (Paralister) cognatus (J.E. LeConte, 1844)

Margarinotus (Paralister) faedatus (J.E. LeConte, 1845)

Margarinotus (Paralister) lecontei Wenzel, 1944

Margarinotus (Promethister) confusus Wenzel, 1944

Margarinotus (Ptomister) brunneus (Fabricius, 1775)†

Margarinotus (Ptomister) egregius (Casey, 1916)

Margarinotus (Ptomister) harrisii (Kirby, 1837)

Margarinotus (Ptomister) hudsonicus (Casey, 1893)

Margarinotus (Ptomister) immunis (Erichson, 1834)

Margarinotus (Ptomister) interruptus (Palisot de Beauvois, 1818)

Margarinotus (Ptomister) meridarius (Hoffmann, 1803)†

Margarinotus (Ptomister) stygicus (J.E. LeConte, 1845)

*Psiloscelis planipes* (LeConte, 1852)

*Spilodiscus arcuatus* (Say, 1825)

**Tribe Platysomatini Bickhardt, 1914**

Platysoma (Cylister) coarctatum J.E. LeConte, 1844

Platysoma (Cylistus) cylindricum (Paykull, 1811)

Platysoma (Cylistus) deficiens (Casey, 1924)

Platysoma (Cylistus) gracile J.E. LeConte, 1845

Platysoma (Platysoma) leconti Marseul, 1853

**Subfamily ONTHOPHILINAE MacLeay, 1819**

***Epierus pulicarius* Erichson, 1834**

**Superfamily STAPHYLINOIDEA Latreille, 1802**

**Family HYDRAENIDAE Mulsant, 1844**

(Minute moss beetles)

**Subfamily HYDRAENINAE Mulsant, 1844**

**Tribe Hydraenini Mulsant, 1844**

*Hydraena angulicollis* Notman, 1922

*Hydraena pensylvanica* Kiesenwetter, 1849

**Subfamily OCHTHEBIINAE C.G. Thomson, 1859**

**Tribe Ochthebiini C.G. Thomson, 1859**

Subtribe Ochthebiina C.G. Thomson, 1859

Ochthebius (Ochthebius) costatellus Reitter, 1897

**Family PTILIIDAE Erichson, 1845**

(Feather-winged beetles)

**Tribe Nanosellini Batrber, 1924**

*Cylindroselloides dybasi* Hall, 1999

**Tribe Ptenidiini Flach, 1889**

*Ptenidium nitidum* (Heer, 1841)†

*Ptenidium pusillum* (Gyllenhal, 1808)†

**Subfamily ACROTRICHINAE Reitter, 1909**

Acrotrichis (Acrotrichis) cognata (Matthews, 1877)

Acrotrichis (Acrotrichis) fascicularis (Herbst, 1793)†

Acrotrichis (Acrotrichis) insularis (Mäklin, 1852)

Acrotrichis (Acrotrichis) intermedia (Gillmeister, 1845)

Acrotrichis (Acrotrichis) josephi (Matthews, 1872)

Acrotrichis (Acrotrichis) longipennis (Casey, 1885)

Acrotrichis (Acrotrichis) parva Rosskothen, 1935*

Acrotrichis (Acrotrichis) sericans (Heer, 1841)†

Acrotrichis (Acrotrichis) silvatica Rosskothen, 1935†

Acrotrichis (Acrotrichis) thoracica (Waltl, 1838)†

Acrotrichis (Acrotrichis) volans (Motschulsky, 1845)*

Acrotrichis (Ctenopteryx) grandicollis (Mannerheim, 1844)†

(incerate sedis)

*Acrotrichus haldemani* (LeConte, 1863)

**Family LEIODIDAE Fleming, 1821**

(Small scavenger beetles)

**Subfamily LEIODINAE Fleming, 1821**

**Tribe Agathidiini Westwood, 1838**

*Agathidium atronitens* Fall, 1934

***Agathidium depressum* Fall, 1934**

*Agathidium difforme* (LeConte, 1850)

*Agathidium exiguum* Melsheimer, 1844

*Agathidium fawcettae* K.B. Miller & Wheeler, 2005

*Agathidium mollinum* Fall, 1934

*Agathidium rubellum* Fall, 1934

*Anisotoma basalis* (LeConte, 1853)

*Anisotoma geminata* (Horn, 1880)

*Anisotoma globososa* Hatch, 1929

*Anisotoma inops* W.J. Brown, 1937

*Anisotoma obsoleta* (Horn, 1880)

**Tribe Leiodini Fleming, 1821**

*Anogdus potens* (W.J. Brown, 1932)

*Cyrtusa subtestacea* (Gyllenhal, 1813)†

*Leiodes assimilis* (LeConte, 1850)

*Leiodes collaris* (LeConte, 1850)

*Leiodes contaminabilis* Baranowski, 1993

*Leiodes impersonata* W.J. Brown, 1932

*Leiodes neglecta* Baranowski, 1993

*Leiodes punctostriata* Kirby, 1837

*Leiodes strigata* (LeConte, 1850)

*Leiodes triepkei* (Schmidt, 1841)*

*Leiodes valida* (Horn, 1880)

*Liocyrtusa luggeri* (Hatch, 1927)

**Tribe Pseudoliodini Portevin, 1926**

Colenus (Colenus) impunctata LeConte, 1853

**Tribe Sogdini Lopatin, 1961**

*Hydnobius pumilus* LeConte, 1879

*Hydnobius substriatus* LeConte, 1863

*Platyhydnobius arizonensis* (Horn, 1885)

**Subfamily COLONINAE Horn, 1880**

Colon (Colon) asperatum Horn, 1880

Colon (Colon) bidentatum (C.R. Sahlberg, 1822)*

Colon (Colon) horni Szymczakowski, 1981

Colon (Eurycolon) magnicolle Mannerheim, 1853

Colon (Myloechus) boreale Peck & Stephan, 1996

**Subfamily Cholevinae Kirby, 1837**

**Tribe Anemadini Hatch, 1928**

Subtribe Nemadina Jeannel, 1936

Nemadus (Laferius) brachyderus (LeConte, 1863)

Nemadus (Nemadus) horni Hatch, 1933

Nemadus (Nemadus) tenuitarsus Jeannel, 1936

Nemadus (Nemadus) triangulum Jeannel, 1936

**Tribe Cholevini Kirby, 1837**

Subtribe Catopina Chaudoir, 1845

*Catops alsiosus* (Horn, 1885)*

*Catops basilaris* Say, 1823

*Catops paramericanus* Peck & Cook, 2002

*Catops simplex* Say, 1825

*Sciodrepoides terminans* (LeConte, 1850)

*Prionochaeta opaca* (Say, 1825)

**Subfamily PLATYPSYLLINAE Ritsema, 1869**

*Leptinillus validus* (Horn, 1872)

**Family SILPHIDAE Latreille, 1806**

(Carrion beetles)

**Subfamily SILPHINAE Latreille, 1806**

*Necrodes surinamensis* (Fabricius, 1775)

*Necrophila americana* (Linnaeus, 1758)

*Oiceoptoma noveboracense* (Forster, 1771)

*Thanatophilus lapponicus* (Herbst, 1793)*

**Subfamily NICROPHORINAE Kirby, 1837**

*Nicrophorus defodiens* Mannerheim, 1846

*Nicrophorus investigator* Zetterstedt, 1824*

*Nicrophorus orbicollis* Say, 1825

*Nicrophorus pustulatus* Herschel, 1807

*Nicrophorus sayi* Laporte, 1840

*Nicrophorus tomentosus* Weber, 1801

*Nicrophorus vespilloides* Herbst, 1783*

**Family STAPHYLINIDAE Latreille, 1802**

(Rove beetles)

**Subfamily OMALIINAE MacLeay, 1825**

**Tribe Anthophagini C.G. Thomson, 1859**

*Acidota crenata* (Fabricius, 1792)*

*Acidota quadrata* (Zetterstedt, 1838)*

*Acidota subcarinata* Erichson, 1840

*Arpedium angulare* Fauvel, 1878

*Brathinus nitidus* LeConte, 1852

*Brathinus varicornis* LeConte, 1852

*Eucnecosum brunnescens* (J.R. Sahlberg, 1871)*

*Eucnecosum tenue* (LeConte, 1863)*

*Geodromicus plagiatus* (Fabricius, 1798)

*Geodromicus strictus* Fauvel, 1889

*Lesteva pallipes* LeConte, 1863

*Microedus austinianus* LeConte, 1874

*Olophrum boreale* (Paykull, 1792)*

*Olophrum consimile* (Gyllenhal, 1810)*

*Olophrum obtectum* Erichson, 1840

*Olophrum rotundicolle* (C.R. Sahlberg, 1830)*

*Porrhodites inflatus* (Hatch, 1957)

*Trigonodemus striatus* LeConte, 1863

**Tribe Coryphiini Jakobson, 1908**

Subtribe Boreaphilina Zerche, 1990

*Boreaphilus henningianus* C.R. Sahlberg, 1832*

Subtribe Coryphiina Jakobson, 1908

*Coryphium nigrum* Campbell, 1978

**Tribe Eusphalerini Hatch, 1957**

Eusphalerum (Eusphalerum) convexum (Fauvel, 1878)

Eusphalerum (Eusphalerum) orientale (Bernhauer, 1912)

*Eusphalerum fenyesi* (Bernhauer, 1912)

Eusphalerum (Eusphalerum) pothos (Mannerheim, 1843)

**Tribe Omaliini MacLeay, 1825**

*Acrolocha diffusa* (Fauvel, 1878)

*Hapalaraea hamata* (Fauvel, 1878)

*Microlymma marinum* (Ström, 1783)*

*Omalium foraminosum* Mäklin, 1852*

*Omalium quadripenne* Casey, 1893

*Omalium rivulare* (Paykull, 1789)†

*Phloeonomus laesicollis* (Mäklin, 1852)

*Phloeostiba lapponica* (Zetterstedt, 1838)*

***Phyllodrepa humerosa* (Fauvel, 1878)**

*Pycnoglypta aptera* Campbell, 1983

*Pycnoglypta campbelli* Gusarov, 1995*

**Subfamily PROTEININAE Erichson, 1839**

**Tribe Proteinini Erichson, 1839**

*Megarthrus angulicollis* Mäklin, 1852

*Megarthrus excisus* LeConte, 1863

*Proteinus acadiensis* Klimaszewski, 2005

***Proteinus hughesi* Webster & Davies^§^**

***Proteinus parvulus* LeConte, 1863**

*Proteinus pseudothomasi* Klimaszewski, 2005

***Proteinus sweeneyi* Webster & Klimaszewski, 2016^§^**

**Subfamily MICROPEPLINAE Leach, 1815**

*Micropeplus browni* Campbell, 1968

*Micropeplus laticollis* Mäklin, 1853

**Subfamily PSELAPHINAE Latreille, 1802**

**Supertribe BATRISITAE Reitter, 1882**

**Tribe Batrisini Reitter, 1882**

Subtribe Batrisina Reitter, 1882

Batrisodes (Babnormodes) riparius (Say, 1824)

Batrisodes (Excavodes) frontalis (LeConte, 1849)

Batrisodes (Excavodes) lineaticollis (Aubé, 1833)

Batrisodes (Excavodes) scabriceps (LeConte, 1849)

**Supertribe EUPLECTITAE Streubel, 1839**

**Tribe Euplectini Streubel, 1839**

Euplectus (Euplectus) acomanus Casey, 1908

Euplectus (Euplectus) confluens LeConte, 1849

Euplectus (Euplectus) duryi Casey, 1908

Euplectus (Euplectus) elongatus Brendel, 1893

*Pycnoplectus linearis* (LeConte, 1849)

**Tribe Trichonychini Reitter, 1882**

Subtribe Bibloporina Park, 1951

*Bibloporus bicanalis* (Casey, 1884)

Subtribe Panaphantina Jeannel, 1950

Bibloplectus (Bibloplectoides) integer (LeConte, 1878)

*Ramecia crinita* (Brendel, 1865)

*Trimioplectus obsoletus* Brendel, 1890

Subtribe Trimiina Bowman, 1934

*Actiastes foveicollis* (LeConte, 1878)

*Actiastes globiferum* (LeConte, 1849)

*Dalmosella tenuis* Casey, 1897

**Supertribe GONIACERITAE Reiter, 1882**

**Tribe Brachyglutini Raffray, 1904**

Subtribe Brachyglutina Raffray, 1904

*Brachygluta abdominalis* (Aubé, 1833)

*Brachygluta luniger* (LeConte, 1849)

*Eutrichites zonatus* (Brendel, 1865)

*Reichenbachia borealis* Casey, 1897

*Reichenbachia corporalis* Casey, 1897

*Reichenbachia propinqua* (LeConte, 1849)

*Reichenbachia spatulifer* Casey, 1897

*Rybaxis clavata* (Brendel, 1865)

*Rybaxis conjuncta* (LeConte, 1849)

*Rybaxis mystica* Casey, 1894

*Rybaxis transversa* Fall, 1927

*Rybaxis varicornis* (Brendel, 1890)

Subtribe Decarthrina Park, 1951

Decarthron (Decarthron) abnorme (LeConte, 1849)

**Tribe Bythinini Raffray, 1890**

*Tychobythinus bythinioides* (Brendel, 1865)

**Tribe Tychini Raffray, 1904**

*Lucifotychus hirsutus* D.S. Chandler, 1991

*Lucifotychus testaceus* (Casey, 1884)

**Supertribe PSELAPHITAE Latreille, 1802**

**Tribe Ctenistini Blanchard, 1845**

*Ctenisodes piceus* (LeConte, 1849)

**Tribe Pselaphini Latreille, 1802**

Pselaphus (Pselaphus) bellax Casey, 1894

Pselaphus (Pselaphus) fustifer Casey, 1894

**Tribe Tyrini Reitter, 1882**

**Subtribe Tyrina Reitter, 1882**

*Tyrus semiruber* Casey, 1897

**Subfamily PHLOEOCHARINAE Erichson, 1839**

*Charhyphus picipennis* (LeConte, 1863)

**Subfamily OLISTHAERINAE C.G. Thomson, 1858**

*Olisthaerus substriatus* (Paykull, 1790)*

**Subfamily TACHYPORINAE MacLeay, 1825**

**Tribe Mycetoporini C.G. Thomson, 1859**

*Bolitobius cingulatus* Mannerheim, 1830*

*Bryophacis smetanai* Campbell, 1993

*Bryoporus rufescens* LeConte, 1863

*Bryoporus testaceus* LeConte, 1863

*Carphacis nepigonensis* (Bernhauer, 1912)

*Ischnosoma fimbriatum* Campbell, 1991

*Ischnosoma flavicolle* (LeConte, 1863)

*Ischnosoma pictum* (Horn, 1877)

*Ischnosoma splendidum* (Gravenhorst, 1806)*

*Ischnosoma virginicum* (Bernhauer, 1917)

Lordithon (Bolitobus) fungicola Campbell, 1982

Lordithon (Bolitobus) kelleyi (Malkin, 1944)

Lordithon (Bolitobus) longiceps (LeConte, 1863)

Lordithon (Bolitobus) quaesitor (Horn, 1877)

Lordithon (Lordithon) anticus (Horn, 1877)

Lordithon (Lordithon) appalachianus Campbell, 1982

Lordithon (Lordithon) axillaris (Gravenhorst, 1806)

Lordithon (Lordithon) campbelli Schülke, 2000

Lordithon (Lordithon) facilis (Casey, 1885)

Lordithon (Lordithon) niger (Gravenhorst, 1802)

Lordithon (Lordithon) scutellaris Campbell, 1982

Lordithon (Lordithon) thoracicus thoracicus (Fabricius)*

*Mycetoporus americanus* Erichson, 1839

*Mycetoporus consors* LeConte, 1863

*Mycetoporus horni* Bernhauer & Schubert, 1916

*Mycetoporus inquisitus* Casey, 1885

*Mycetoporus lucidulus* LeConte, 1863

***Mycetoporus rufohumeralis* Campbell, 1991**

*Mycetoporus rugosus* Hatch, 1957

*Mycetoporus triangulatus* Campbell, 1991

**Tribe Tachyporini MacLeay, 1825**

*Cilea silphoides* (Linnaeus)†

*Coproporus ventriculus* (Say, 1832)

*Nitidotachinus horni* (Campbell, 1973)

*Nitidotachinus scrutator* (Gemminger & Harold, 1868)

*Nitidotachinus tachyporoides* (Horn, 1877)

*Sepedophilus cinctulus* (Erichson, 1839)

*Sepedophilus crassus* (Gravenhorst, 1802)

***Sepedophilus immaculatus* (Stephens, 1832)**†

*Sepedophilus littoreus* (Linnaeus, 1758)†

*Sepedophilus marshami* (Stephens, 1832)†

*Sepedophilus occultus* (Casey, 1884)

*Sepedophilus testaceus* (Fabricius, 1792)†

*Sepedophilus versicolor* (Casey, 1884)

Tachinus (Tachinus) addendus Horn, 1877

Tachinus (Tachinus) basalis Erichson, 1839*

Tachinus (Tachinus) canadensis Horn, 1877

Tachinus (Tachinus) corticinus Gravenhorst, 1802†

Tachinus (Tachinus) elongatus Gyllenhal, 1810*

Tachinus (Tachinus) fimbriatus Gravenhorst, 1802

Tachinus (Tachinus) frigidus Erichson, 1839

Tachinus (Tachinus) fumipennis (Say, 1832)

Tachinus (Tachinus) limbatus Melsheimer, 1844

Tachinus (Tachinus) luridus Erichson, 1840

Tachinus (Tachinus) memnonius Gravenhorst, 1802

Tachinus (Tachinus) picipes Erichson, 1839

Tachinus (Tachinus) quebecensis Robert, 1946

Tachinus (Tachinus) rufipes (Linnaeus, 1758)†

Tachinus (Tachinus) schwarzi Horn, 1877

Tachinus (Tachinus) thruppi Hatch, 1957

Tachinus (Tachinus) vergatus Campbell, 1973

Tachyporus (Palporus) nitidulus (Fabricius, 1781)*

Tachyporus (Tachyporus) abdominalis (Fabricius, 1781)*

Tachyporus (Tachyporus) browni Campbell, 1979

Tachyporus (Tachyporus) canadensis Campbell, 1979

Tachyporus (Tachyporus) dispar (Paykull, 1789)†

Tachyporus (Tachyporus) flavipennis Campbell, 1979

Tachyporus (Tachyporus) inornatus Campbell, 1979

Tachyporus (Tachyporus) lecontei Campbell, 1979

Tachyporus (Tachyporus) maculicollis LeConte, 1866

Tachyporus (Tachyporus) nanus Erichson, 1839

Tachyporus (Tachyporus) nimbicola Campbell, 1979

Tachyporus (Tachyporus) pulchrus Blatchley, 1910

Tachyporus (Tachyporus) rulomoides Campbell, 1979

Tachyporus (Tachyporus) transversalis Gravenhorst, 1806†

**Subfamily TRICHOPHYINAE C.G. Thomson, 1858**

*Trichophya pilicornis* (Gyllenhal, 1810)†

**Subfamily HABROCERINAE Mulsant & Rey, 1876**

*Habrocerus capillaricornis* (Gravenhorst, 1806)†

*Habrocerus schwarzi* Horn, 1877

(incertae sedis)

*Habrocerus magnus* LeConte, 1878

**Subfamily ALEOCHARINAE Fleming, 1821**

**Tribe Aleocharini Fleming, 1821**

Subtribe Aleocharina Fleming, 1821

Aleochara (Aleochara) curtula (Goeze, 1777)†

Aleochara (Aleochara) gracilicornis Bernhauer, 1901

Aleochara (Aleochara) sekanai Klimazewski, 1985

Aleochara (Aleochara) tahoensis Casey, 1906

Aleochara (Aleochara) thoracica (Casey, 1894)

**Aleochara (Calochara) rubricalis (Casey, 1911)**

Aleochara (Calochara) rubripennis (Casey, 1906)

**Aleochara (Calochara) speculicollis Bernhauer, 1901**

Aleochara (Calochara) villosa Mannerheim, 1830†

Aleochara (Coprochara) bilineata Gyllenhal, 1810†

Aleochara (Coprochara) bimaculata Gravenhorst, 1802

Aleochara (Coprochara) verna Say, 1833

**Aleochara (Echochara) ocularis Klimaszewski, 1984**

Aleochara (Emplenota) litoralis (Mäklin, 1853)

Aleochara (Xenochara) castaneipennis Mannerheim, 1843

Aleochara (Xenochara) fumata Gravenhorst, 1802†

Aleochara (Xenochara) inexpectata Klimaszewski, 1984

Aleochara (Xenochara) lacertina Sharp, 1883

Aleochara (Xenochara) lanuginosa Gravenhorst, 1802†

Aleochara (Xenochara) sculptiventris (Casey, 1894)

Aleochara (Xenochara) tristis Gravenhorst, 1806†

*Amarochara formicina* Assing, 2007

*Amarochara inquilina* (Casey, 1906)

***Tinotus caviceps* Casey, 1894**

*Tinotus morion* (Gravenhorst, 1802)†

**Tribe Athetini Casey, 1910**

Subtribe Athetina Casey, 1910

***Acrotona brachyoptera* Klimaszewski & Webster, 2016^§^**

*Acrotona sequestralis* (Casey, 1910)

*Acrotona smithi* (Casey, 1910)

***Acrotona sphagnorum* Klimaszewski & Webster, 2016^§^**

***Acrotona subpygmaea* (Bernhauer, 1909)**

***Alevonota gracilenta* (Erichson, 1839)**†

*Aloconota neocambrica* Klimaszewski & Langor, 2011

*Aloconota sulcifrons* (Stephens, 1832)†

*Amisha analis* (Gravenhorst, 1802)†

Atheta (Alaobia) ventricosa Bernhauer, 1907

Atheta (Atheta) aemula (Erichson, 1839)

Atheta (Atheta) circulicollis Lohse, 1990

Atheta (Atheta) graminicola (Gravenhorst, 1806)*

Atheta (Chaetida) longicornis (Gravenhorst, 1802)†

Atheta (Datomicra) acadiensis Klimaszewski & Majka, 2007

Atheta (Datomicra) celata (Erichson, 1837)†

Atheta (Datomicra) dadopora C.G. Thomson, 1867*

Atheta (Datomicra) particula (Casey, 1910)

**Atheta (Datomicra) whitehorsensis Klimaszewski & Godin, 2012**

**Atheta (Dimetrota) alphacrenuliventris Klimaszewski & Webster, 2016^§^**

**Atheta (Dimetrota) bubo Klimaszewski & Webster, 2016^§^**

Atheta (Dimetrota) burwelli (Lohse, 1990)

**Atheta (Dimetrota) campbelli (Lohse, 1990)**

**Atheta (Dimetrota) chartersensis Klimaszewski & Webster, 2016^§^**

**Atheta (Dimetrota) cranberriensis Klimaszewski & Webster, 2016^§^**

Atheta (Dimetrota) crenuliventris Bernhauer, 1907

Atheta (Dimetrota) districta Casey, 1911

Atheta (Dimetrota) fanatica Casey, 1910

**Atheta (Dimetrota) giguereae Klimaszewski & Webster, 2016^§^**

Atheta (Dimetrota) hampshirensis Bernhauer, 1909

**Atheta (Dimetrota) makepeacei Klimaszewski & Webster, 2016^§^**

**Atheta (Dimetrota) mcalpinei Klimaszewski & Webster, 2016^§^**

Atheta (Dimetrota) modesta (Melsheimer, 1844)

**Atheta (Dimetrota) petitcapensis Klimaszewski & Webster, 2016^§^**

Atheta (Dimetrota) prudhoensis (Lohse, 1990)

Atheta (Dimetrota) pseudocrenuliventris Klimaszewski, 2005

Atheta (Dimetrota) pseudomodesta Klimaszewski, 2007

**Atheta (Dimetrota) sphagnicola Klimaszewski & Webster, 2016^§^**

Atheta (Dimetrota) strigosula Casey, 1910

Atheta (Dimetrota) terranovae Klimaszewski & Langor, 2011

Atheta (Metadimetrota) savardae Klimaszewski & Majka, 2007

Atheta (Microdota) curtipenis Klimaszewski & Webster, 2015

Atheta (Microdota) formicaensis Klimaszewski & Webster, 2015

Atheta (Microdota) macesi Klimaszewski & Webster, 2015

Atheta (Microdota) pennsylvanica Bernhauer, 1907

Atheta (Microdota) platanoffi Brundin, 1948*

Atheta (Microdota) pseudosubtilis Klimaszewski & Langor, 2011

Atheta (Microdota) subtilis (Scriba, 1866)†

Atheta (Pseudota) annexa Casey, 1910

Atheta (Pseudota) klagesi Bernhauer, 1909

**Atheta (Pseudota) pseudoklagesi Klimaszewski & Webster, 2016^§^**

Atheta (Tetropla) frosti Bernhauer, 1909

Atheta (Thinobaena) vestita (Gravenhorst, 1806)†

(incertae sedis)

*Atheta brunswickensis* Klimaszewski, 2005

*Atheta capsularis* Klimaszewski, 2005

*Atheta novaescotiae* Klimaszewski & Majka, 2006

*Atheta remulsa* Casey, 1910

***Atheta pseudoschistoglossa* Klimaszewski & Webster, 2016^§^**

***Atheta thujae* Klimaszewski & Webster, 2016^§^**

*Boreophilia eremita* (Rye, 1866)*

*Boreostiba websteri* Klimaszewski & Langor, 2011

*Clusiota grandipenis* Klimaszewski & Webster, 2015

*Clusiota impressicollis* (Bernhauer, 1907)

*Dalotia coriaria* (Kraatz, 1856)†

*Dinaraea angustula* (Gyllenhal, 1810)†

*Dinaraea backusensis* Klimaszewski & Brunke, 2012

*Dinaraea bicornis* Klimaszewski & Webster, 2013

*Dinaraea borealis* Lohse, 1990

*Dinaraea curtipenis* Klimaszewski & Webster, 2013

*Dinaraea longipenis* Klimaszewski & Webster, 2013

*Dinaraea pacei* Klimaszewski & Langor, 2011

*Dinaraea quadricornis* Klimaszewski & Webster, 2013

***Dinaraea subdepressa* (Bernhauer, 1907)**

*Dochmonota rudiventris* (Eppelsheim, 1886)*

*Earota dentata* (Bernhauer, 1906)

Geostiba (Geostiba) circellaris (Gravenhorst, 1806)†

Geostiba (Sibiota) appalachigena Gusarov, 2002

*Hydrosmecta newfoundlandica* Klimaszewski & Langor, 2011

*Hydrosmecta pseudodiosica* Lohse, 1990

***Liogluta castoris* Klimaszewski & Webster, 2016^§^**

***Liogluta microgranulosa* Klimaszewski & Webster, 2016^§^**

***Liogluta pseudocastoris* Klimaszewski & Webster, 2016^§^**

*Liogluta terminalis* (Casey, 1906)

*Liogluta aloconotoides* Lohse, 1990

*Lypoglossa angularis obtusa* (LeConte, 1866)

*Lypoglossa franclemonti* Hoebeke, 1992

*Mocyta breviuscula* (Mäklin, 1852)

*Mocyta luteola* (Erichson, 1839)

*Mocyta fungi* (Gravenhorst, 1806)†

*Mocyta sphagnorum* Klimaszewski & Webster, 2015.

*Nehemitropia lividipennis* (Mannerheim, 1830)†

***Paragoniusa myrmicae* Maruyama & Klimaszewski, 2004**

*Philhygra angusticauda* (Bernhauer, 1909)

***Philhygra atypicalis* Klimaszewski & Webster, 2016^§^**

*Philhygra botanicarum* (Muona, 1983)*

*Philhygra clemens* (Casey, 1910)

***Philhygra hygrotopora* (Kraatz, 1856)**‡

*Philhygra jarmilae* Klimaszewski & Langor, 2011

*Philhygra laevicollis* (Mäklin, 1852)

***Philhygra larsoni* Klimaszewski & Langor, 2011**

*Philhygra luridipennis* (Mannerheim, 1830)

***Philhygra proterminalis* (Bernhauer, 1907)**

***Philhygra pseudolarsoni* Klimaszewski & Godin, 2012**

*Philhygra sinuipennis* Klimaszewski & Langor, 2011

***Philhygra terrestris* Klimaszewski & Godin, 2012**

*Philhygra varula* (Casey, 1911)

Schistoglossa (Boreomorpha) blatchleyi (Bernhauer & Scheerpeltz, 1926)

Schistoglossa (Boreomorpha) sphagnorum Klimaszewski & Webster, 2009

Schistoglossa (Schistoglossa) brunswickensis Klimaszewski & Webster, 2009

**Schistoglossa (Schistoglossa) pelletieri Klimaszewski & Webster, 2016^§^**

Schistoglossa (Schistoglossa) pseudocampbelli Klimaszewski & Webster, 2009

***Seeversiella globicollis* (Bernhauer, 1907)**

***Strigota ambigua* (Erichson, 1839)**

***Strigota obscurata* Klimaszewski & Brunke, 2012**

*Strophogastra pencillata* Fenyes, 1921

*Tomoglossa decora* (Casey, 1910)

***Trichiusa hirsuta* Casey, 1906**

***Trichiusa pilosa* Casey, 1894**

***Trichiusa robustula* Casey, 1894**

Subtribe Thamiaraeina Fenyes, 1921

*Thamiaraea brittoni* (Casey, 1911)

***Thamiaraea claydeni* Klimaszewski & Webster, 2016^§^**

***Thamiaraea corverae* Klimaszewski & Webster, 2016^§^**

**Tribe Autaliini C.G. Thomson, 1859**

*Autalia rivularis* (Gravenhorst, 1802)†

**Tribe Boreocyphini Klimaszewski & Langor, 2011**

*Boreocypha websteri* Klimaszewski & Langor, 2011

**Tribe Deinopsini Sharp, 1883**

*Deinopsis canadensis* Klimaszewski, 1979

*Deinopsis harringtoni* Casey, 1911

*Deinopsis rhadina* Klimaszewski, 1979

**Tribe Diglottini Jakobson, 1909**

*Diglotta mersa* (Haliday, 1837)†

**Tribe Falagriini Mulsant & Rey, 1873**

*Cordalia obscura* (Gravenhorst, 1802)†

*Falagria caesa* Erichson, 1837†

*Falagria dissecta* Erichson, 1839

*Myrmecocephalus gatineauensis* Hoebeke, 1985

***Myrmecopora vaga* (LeConte, 1866)**

**Tribe Gymnusini Heer, 1839**

*Gymnusa atra* Casey, 1911*

*Gymnusa campbelli* Klimaszewski, 1979

*Gymnusa grandiceps* Casey, 1915

*Gymnusa pseudovariegata* Klimaszewski, 1979

**Tribe Homalotini Heer, 1839**

Subtribe Bolitocharina C.G. Thomson, 1859

Leptusa (Adoxopisalia) opaca Casey, 1894

Leptusa (Boreoleptusa) canonica Casey, 1906

Leptusa (Boreoleptusa) jucunda Klimaszewski & Majka, 2004

Leptusa (Dysleptusa) carolinensis Pace, 1989

Leptusa (Eucryptusa) brevicollis Casey, 1894

(incertae sedis)

*Leptusa gatineauensis* Klimaszewski & Pelletier, 2004

*Neotobia alberta* Ashe, 1992

*Phymatura blanchardi* (Casey, 1894)

***Pleurotobia bourdonae* Klimaszewski & Webster, 2016^§^**

***Pleurotobia brunswickensis* Klimaszewski & Webster, 2016^§^**

*Silusida marginella* (Casey, 1894)

Subtribe Gyrophaenina Kraatz, 1856

***Agaricomorpha vincenti* Klimaszewski & Webster, 2016^§^**

*Agaricomorpha websteri* Klimaszewski & Brunke, 2012

*Eumicrota corruscula* (Erichson, 1839)

*Eumicrota socia* (Erichson, 1839)

Gyrophaena (Gyrophaena) affinis Mannerheim, 1830†

**Gyrophaena (Gyrophaena) aldersonae Klimaszewski & Webster, 2016^§^**

Gyrophaena (Gyrophaena) antennalis Casey, 1906

**Gyrophaena (Gyrophaena) brevicollis Seevers, 1951**

Gyrophaena (Gyrophaena) caseyi Seevers, 1951

Gyrophaena (Gyrophaena) chippewa Seevers, 1951

Gyrophaena (Gyrophaena) criddlei Casey, 1911

Gyrophaena (Gyrophaena) dybasi Seevers, 1951

Gyrophaena (Gyrophaena) flavicornis Melsheimer, 1844

Gyrophaena (Gyrophaena) fuscicollis Casey, 1906

Gyrophaena (Gyrophaena) gilvicollis Casey, 1906

Gyrophaena (Gyrophaena) illiana Seevers, 1951

Gyrophaena (Gyrophaena) insolens Casey, 1906

Gyrophaena (Gyrophaena) involuta Casey, 1906

Gyrophaena (Gyrophaena) keeni Casey, 1911

Gyrophaena (Gyrophaena) laetula Casey, 1906

Gyrophaena (Gyrophaena) lobata Casey, 1906

Gyrophaena (Gyrophaena) michigana Seevers, 1951

Gyrophaena (Gyrophaena) modesta Casey, 1906

Gyrophaena (Gyrophaena) nana (Paykull, 1800)*

Gyrophaena (Gyrophaena) nanoides Seevers, 1951

Gyrophaena (Gyrophaena) neonana Seevers, 1951

Gyrophaena (Gyrophaena) pseudocriddlei Klimaszewski & Webster, 2009

Gyrophaena (Gyrophaena) sculptipennis Casey, 1906

Gyrophaena (Gyrophaena) uteana Casey, 1906

Gyrophaena (Gyrophaena) vitrina Casey, 1911

Gyrophaena (Gyrophaena) wisconsinica Seevers, 1951

Gyrophaena (Phaenogyra) gracilis Seevers, 1951

Gyrophaena (Phaenogyra) meduxnekeagensis Klimaszewski & Webster, 2009

Gyrophaena (Phaenogyra) subnitens Casey, 1906

Subtribe Homalotina Heer, 1839

***Anomognathus americanus* (Casey, 1894)**

*Homolota plana* (Gyllenhal, 1810)†

Subtribe Silusina Fenyes, 1918

*Silusa alternans* Sachse, 1852

*Silusa californica* Bernhauer, 1905

*Silusa densa* Fenyes, 1909

*Silusa langori* Klimaszewski, 2003

**Tribe Hoplandriini Casey, 1910**

Subtribe Hoplandriina Casey, 1910

Hoplandria (Hoplandria) lateralis (Melsheimer, 1844)

**Hoplandria (Lophomucter) laevicollis (Notman, 1920)**

**Tribe Hypocyphtini Laporte, 1835**

***Oligota chrysopyga* Kraatz, 1859**†

***Oligota parva* Kraatz, 1862**†

***Oligota polyporicola* Klimaszewski & Webster, 2016^§^**

***Oligota pusillima* Gravenhorst, 1806**†

***Oligota sevogle* Klimaszewski & Webster, 2016^§^**

**Tribe Lomechusini Fleming, 1821**

Subtribe Lomechusina Fleming, 1821

*Xenodusa reflexa* (Walker, 1866)

Subtribe Myrmedoniina C.G. Thomson, 1867

*Drusilla canaliculata* (Fabricius, 1787)†

*Meronera venustula* (Erichson, 1837)

*Pella gesneri* Klimaszewski, 2005

*Zyras obliquus* (Casey, 1894)

**Tribe Myllaenini Ganglbauer, 1895**

*Myllaena arcana* Casey, 1911

*Myllaena audax* Casey, 1911

*Myllaena insomnis* Casey, 1911

*Myllaena kaskaskia* Klimaszewski, 1982

*Myllaena ludificans* Casey, 1911

*Myllaena procidua* Casey, 1911

*Myllaena vulpina* Bernhauer, 1907

**Tribe Oxypodini C.G. Thomson, 1859**

Subtribe Dinardina Mulsant & Rey, 1873

***Blepharhymenus brendeli* (Casey, 1894)**

Subtribe Meoticina Seevers, 1978

*Alisalia elongata* Klimaszewski & Webster, 2009

*Alisalia minuta* Klimaszewski & Webster, 2009

*Alisalia testacea* Casey, 1911

***Meotica pallens* (Redtenbacher, 1849)**†

Subtribe Microglottina Fenyes, 1918

*Crataraea suturalis* (Mannerheim, 1830)†

Subtribe Oxypodina C.G. Thomson, 1859

***Calodera caseyi* Assing, 2002**

*Calodera parviceps* (Casey, 1894)

*Devia prospera* (Erichson, 1839)*

***Dexiogyia angustiventris* (Casey, 1894)**

*Gennadota canadensis* Casey, 1906

*Gnathusa tenuicornis* Fenyes, 1921

***Hylota cryptica* Klimaszewski & Webster, 2016^§^**

*Hylota ochracea* Casey, 1906

*Ilyobates bennetti* Donisthorpe, 1914†

*Mniusa minutissima* (Klimaszewski & Langor, 2011)

*Mniusa odelli* Klimaszewski & Webster, 2014

*Mniusa yukonensis* (Klimaszewski & Godin, 2012)

***Neothetalia canadiana* Klimaszewski, 2004**

*Ocyusa asperula* Casey, 1894

*Ocyusa canadensis* Lohse, 1990

Oxypoda (Bessopora) brachyptera (Stephens, 1832)†

Oxypoda (Oxypoda) grandipennis (Casey, 1911)

Oxypoda (Oxypoda) opaca (Gravenhorst, 1802)†

Oxypoda (Podoxya) amica Casey, 1906

(incertae sedis)

*Oxypoda convergens* Casey, 1894

*Oxypoda demissa* Casey, 1911

*Oxypoda frigida* Bernhauer, 1907

*Oxypoda gnara* Casey, 1911

*Oxypoda hiemalis* Casey, 1911

*Oxypoda inimica* Casey, 1911

*Oxypoda lacustris* Casey, 1906

*Oxypoda lucidula* Casey, 1906

*Oxypoda nigriceps* Casey, 1894

*Oxypoda orbicollis* Casey, 1911

*Oxypoda pseudolacustris* Klimaszewski, 2006

***Oxypoda sunpokeana* Klimaszewski & Webster, 2016^§^**

*Oxypoda vockerothi* Klimaszewski, 2006

***Parocyusa americana* (Casey, 1906)**

***Parocyusa fuliginosa* (Casey, 1906)**

Subtribe Phloeoporina C.G. Thomson, 1859

***Phloeopora canadensis* Klimaszewski & Langor, 2011**

***Phloeopora gilbertae* Klimaszewski & Webster, 2016^§^**

*Phloeopora oregona* Casey, 1906

**Tribe Placusini Mulsant & Rey, 1871**

*Euvira micmac* Klimaszewski & Majka, 2007

*Placusa incompleta* Sjöberg, 1934†

*Placusa tachyporoides* (Walt, 1838)†

*Placusa tacomae* Casey, 1894

*Placusa vaga* Casey, 1911

**Tribe Tachyusini C.G. Thomson, 1859**

*Brachyusa helenae* (Casey, 1911)

*Gnypeta atrolucens* Casey, 1894

*Gnypeta caerula* (C.R. Sahlberg, 1830)*

*Gnypeta carbonaria* (Mannerheim, 1830)*

*Gnypeta minuta* Klimaszewski & Webster, 2008

*Gnypeta nigrella* (LeConte, 1863)

*Gnypeta saccharina* Klimaszewski & Webster, 2008

Tachyusa (Tachyusa) americana Casey, 1906

Tachyusa (Tachyusa) americanoides Paśnik, 2006

Tachyusa (Tachyusa) obsoleta Casey, 1906

**Tribe Taxicerini Lohse, 1989**

*Halobrecta flavipes* C.G. Thomson, 1861†

**Subfamily SCAPHIDIINAE Latreille, 1806**

**Tribe Scaphidiini Latreille, 1806**

*Scaphidium quadriguttatum* Say, 1823

**Tribe Scaphiini Achard, 1924**

*Scaphium castanipes* Kirby, 1837

**Tribe Scaphisomatini Casey, 1893**

*Baeocera apicalis* LeConte, 1860

*Baeocera deflexa* Casey, 1893

*Baeocera indistincta* Löbl & Stephan, 1993

*Baeocera inexspectata* Löbl & Stephan, 1993

*Baeocera securiforma* (Cornell, 1967)

*Baeocera youngi* (Cornell, 1967)

*Scaphisoma convexum* Say, 1825

*Scaphisoma repandum* Casey, 1893

*Scaphisoma rubens* Casey, 1893

*Toxidium gammaroides* LeConte, 1860

**Subfamily PIESTINAE Erichson, 1839**

*Siagonium punctatum* (LeConte, 1866)

*Siagonium stacesmithi* Hatch, 1957

**Subfamily OSORIINAE Erichson, 1839**

**Tribe Thoracophorini Reitter, 1909**

Subtribe Clavilispinina Newton & Thayer, 1992

*Clavilispinus prolixus* (LeConte, 1877)

Subtribe Thoracophorina Reitter, 1909

*Thoracophorus costalis* (Erichson, 1840)

**Subfamily OXYTELINAE Fleming, 1821**

**Tribe Blediini Ádám, 2001**

*Bledius annularis* LeConte, 1863

*Bledius neglectus* Casey, 1890

***Bledius opaculus* LeConte, 1863**

*Bledius philadelphicus* Fall, 1919

*Bledius politus* Erichson, 1840

*Bledius tau* LeConte, 1877

**Tribe Coprophilini Heer, 1839**

*Coprophilus castoris* Campbell, 1979

*Coprophilus striatulus* (Fabricius, 1792)†

**Tribe Oxytelini Fleming, 1821**

*Anotylus insecatus* (Gravenhorst, 1806)†

*Anotylus rugosus* (Fabricius, 1775)†

*Anotylus tetracarinatus* (Block, 1799)†

*Apocellus sphaericollis* (Say, 1831)

***Carpelimus difficilis* (Casey, 1889)**

***Carpelimus erichsoni* (Sharp, 1871)**†

***Carpelimus gracilis* (Mannerheim, 1831)**†

***Carpelimus lacustris* (Notman, 1924)**

***Carpelimus mundus* (Sharp, 1876)**†

***Carpelimus probus* (Casey, 1889)**

***Carpelimus pusillus* (Gravenhorst, 1802)**†

***Carpelimus quadripunctatus* (Say, 1831)**

***Carpelimus rivularis* (Motschulsky, 1860)**

***Carpelimus spretus* (Casey, 1889)**

***Carpelimus subtilis* (Erichson, 1839)**†

***Carpelimus weissi* (Notman, 1924)**

*Ochthephilus ashei* Makranczy, 2014

*Ochthephilus forticornis* (Hochhuth, 1860)*

*Ochthephilus planus* (LeConte, 1861)*

Oxytelus (Epomotylus) sculptus Gravenhorst, 1806†

Oxytelus (Tanycraerus) lacqueatus (Marsham, 1802)†

Oxytelus (Tanycraerus) montanus Casey, 1893

(incertae sedis)

*Oxytelus incisus* Motschulsky, 1857

**Platystethus (Craetopycrus) degener Mulsant & Rey, 1878**†

Platystethus (Platystethus) americanus Erichson, 1840

***Thinodromus corvinus* (Casey, 1889)**

**Tribe Syntomiini Böving & Craighead, 1931**

*Deleaster dichrous* (Gravenhorst, 1802)†

*Mitosynum vockerothi* Campbell, 1982

*Syntomium grahami* Hatch, 1957

**Subfamily OXYPORINAE Fleming, 1821**

Oxyporus (Oxyporus) kiteleyi Campbell, 1978

Oxyporus (Oxyporus) major Gravenhorst, 1806

Oxyporus (Oxyporus) rufipennis LeConte, 1863

Oxyporus (Oxyporus) stygicus Say, 1831

Oxyporus (Oxyporus) vittatus vittatus Gravenhorst, 1802

Oxyporus (Pseudoxyporus) lateralis Gravenhorst, 1802

Oxyporus (Pseudoxyporus) occipitalis Fauvel, 1864

Oxyporus (Pseudoxyporus) quinquemaculatus LeConte, 1863

**Subfamily SCYDMAENINAE Leach, 1815**

**Supertribe SCYDMAENITAE Leach, 1815**

**Tribe Glandulariini Schaufuss, 1889**

***Brachycepsis subpunctata* (LeConte, 1852)**

Euconnus (Napochus) debilitans (Casey, 1897)

Euconnus (Napochus) flavitarsis (LeConte, 1852)

Euconnus (Napochus) gaudens (Casey, 1897)

Euconnus (Psomophus) fatuus (LeConte, 1852)

Euconnus (Xestophus) salinator (LeConte, 1852)

*Microscydmus misellus* (LeConte, 1852)

*Parascydmus corpusculus* (Casey, 1897)

Stenichnus (Stenichnus) turbatus (Casey, 1897)

**Subfamily STENINAE MacLeay, 1825**

*Dianous chalybaeus* LeConte, 1863

***Dianous nitidulus* LeConte, 1874**

Stenus (Hemistenus) sibiricus J.R. Sahlberg, 1880*

Stenus (Hypostenus) advena (Casey, 1884)

Stenus (Hypostenus) alexanderi Puthz, 1971*

Stenus (Hypostenus) annularis Erichson, 1840

Stenus (Hypostenus) arculus Erichson, 1840

Stenus (Hypostenus) caenicolus Notman, 1919

**Stenus (Hypostenus) destitutus Puthz, 2001**

Stenus (Hypostenus) dissentiens (Casey, 1884)

Stenus (Hypostenus) flavicornis Erichson, 1840)

Stenus (Hypostenus) lugens (Casey, 1884)

**Stenus (Hypostenus) punctatus Erichson, 1840**

Stenus (Hypostenus) quebecensis Puthz, 1971

Stenus (Hypostenus) reconditus reconditus (Casey, 1884)

Stenus (Hypostenus) rossi Sanderson, 1958

Stenus (Hypostenus) varipes (Casey, 1884)

Stenus (Metatesnus) croceatus (Casey, 1884)

Stenus (Metatesnus) niveus Fauvel, 1865*

Stenus (Metatesnus) pubescens fraternus (Casey, 1884)

Stenus (Stenus) ageus Casey, 1884*

Stenus (Stenus) angustus Casey, 1884

Stenus (Stenus) austini Casey, 1884

Stenus (Stenus) brivioi Puthz, 1972

Stenus (Stenus) canaliculatus Gyllenhal, 1827*

**Stenus (Stenus) carinicollis Casey, 1884**

Stenus (Stenus) caseyi Puthz, 1972

Stenus (Stenus) clavicornis (Scopoli, 1763)†

Stenus (Stenus) colonus Erichson, 1840

Stenus (Stenus) comma comma LeConte, 1863*

**Stenus (Stenus) difficilis Casey, 1884**

Stenus (Stenus) dolosus Casey, 1884

**Stenus (Stenus) egenulus Puthz, 1988**

Stenus (Stenus) egenus Erichson, 1840

Stenus (Stenus) erythropus Melsheimer, 1844

Stenus (Stenus) fasciculatus J.R. Sahlberg, 1871*

**Stenus (Stenus) fulvoguttatus Notman, 1920**

Stenus (Stenus) intrusus Casey, 1884*

Stenus (Stenus) juno (Paykull, 1789)*

Stenus (Stenus) laccophilus Casey, 1884

Stenus (Stenus) lustrator Erichson, 1839†

Stenus (Stenus) mammops mammops Casey, 1884*

Stenus (Stenus) melanarius melanarius Stephens, 1833*

Stenus (Stenus) morio Gravenhorst, 1806*

Stenus (Stenus) neglectus Casey, 1884

**Stenus (Stenus) pluto Casey, 1884**

Stenus (Stenus) pumilio Erichson, 1839*

Stenus (Stenus) pusillimus Puthz, 1971

Stenus (Stenus) scabiosus Casey, 1884

Stenus (Stenus) schwarzi Casey, 1884

Stenus (Stenus) scrupeus Casey, 1884

Stenus (Stenus) semicolon LeConte, 1863

Stenus (Stenus) sordidus Puthz, 1988

Stenus (Stenus) sphaerops Casey, 1884

Stenus (Stenus) strangulatus Casey, 1884

Stenus (Stenus) stygicus Say, 1831

**Stenus (Stenus) vicinus Casey, 1884**

Stenus (Stenus) vinnulus Casey, 1884*

Stenus (Tesnus) formicetorum Mannerheim, 1843*

Stenus (Tesnus) immarginatus Mäklin, 1853*

**Subfamily EUAESTHETINAE C.G. Thomson, 1859**

**Tribe Euaesthetini C.G. Thomson, 1859**

***Edaphus lederi* Eppelsheim, 1878**†

*Euaesthetus albertae* Puthz, 1998

*Euaesthetus duplex* Herman, 2001

*Euaesthetus americanus* Erichson, 1840

*Euaesthetus blanchardi* Puthz, 2014

*Euaesthetus chantali* Puthz, 1998

*Euaesthetus floridae* Casey, 1884

*Euaesthetus ganglbaueri* Bernhauer, 1928

*Euaesthetus hermani* Puthz, 2014

*Euaesthetus iripennis* Casey, 1884

*Euaesthetus laeviusculus* Mannerheim, 1844

*Euaesthetus mundulus* Casey, 1884

*Euaesthetus pugetensis* Hatch, 1957

*Euaesthetus similis* Casey, 1884

*Euaesthetus websteri* Puthz, 2014

**Subfamily PSEUDOPSINAE Ganglbauer, 1895**

***Pseudopsis sagitta* Herman, 1975**

*Pseudopsis subulata* Herman, 1975

**Subfamily PAEDERINAE Fleming, 1821**

**Tribe Paederini Fleming, 1821**

Subtribe Astenina Hatch, 1957

***Astenus americanus* (Casey, 1905)**

***Astenus brevipennis* (Austin, 1876)**

*Astenus cinctus* (Say, 1831)

*Astenus discopunctatus* (Say, 1831)

Subtribe Cryptobiina Casey, 1905

Homaeotarsus (Gastrolobium) bicolor (Gravenhorst, 1802)

Homaeotarsus (Hesperobium) cinctus (Say, 1830)

Homaeotarsus (Hesperobium) cribratus (LeConte, 1863)

Homaeotarsus (Hesperobium) pallipes (Gravenhorst, 1802)

*Ochthephilum fracticorne* (Paykull, 1800)†

Subtribe Lathrobiina Laporte, 1835

Lathrobium (Lathrobioma) othioides LeConte, 1880

Lathrobium (Lathrobioma) scolopaceum (Casey, 1905)

Lathrobium (Lathrobium) amplipenne Casey, 1905

Lathrobium (Lathrobium) armatum Say, 1830

Lathrobium (Lathrobium) confusum LeConte, 1880

Lathrobium (Lathrobium) fauveli Duvivier, 1883

Lathrobium (Lathrobium) fulvipenne (Gravenhorst, 1806)†

Lathrobium (Lathrobium) simile LeConte, 1863

Lathrobium (Lathrobium) sparsellum Casey, 1905

Lathrobium (Lathrobium) spissicorne Casey, 1905

Lathrobium (Lathrobium) washingtoni Casey, 1905

Lathrobium (Lathrolepta) debile LeConte, 1880

Lobrathium (Eulathrobium) grande (LeConte, 1863)

Lobrathium (Lobrathium) collare (Erichson, 1840)

*Tetartopeus angularis* (LeConte, 1863)

*Tetartopeus captiosus* Casey, 1905

*Tetartopeus furvulus* Casey, 1905

*Tetartopeus lacustris* Casey, 1905

*Tetartopeus niger* (LeConte, 1863)

*Tetartopeus nitidulus* (LeConte, 1880)

*Tetartopeus rubripennis* Casey, 1905

Subtribe Medonina Casey, 1905

Achenomorphus (Aderocharis) corticinus (Gravenhorst, 1802)

*Lithocharis ochracea* (Gravenhorst, 1802)†

**Medon (Medon) fusculus (Mannerheim, 1830)**†

Pseudomedon (Pseudomedon) thoracicus Casey, 1905

Sunius (Trachysectus) confluentus (Say, 1831)

Subtribe Paederina Fleming, 1821

*Paederus littorarius* Gravenhorst, 1806

Subtribe Scopaeina Mulsant & Rey, 1878

**Orus (Pycnorus) dentiger (LeConte, 1880)**

**Scopaeus (Scopaeus) minutus Erichson, 1840**†

Subtribe Stilicina Casey, 1905

*Pachystilicus hanhami* (Wickham, 1898)

*Rugilis angustatus* (Geoffroy, 1785)†

*Rugilis biarmatus* (LeConte, 1880)

***Rugilis ceylanensis* (Kraatz, 1859)**†

*Rugilis rufipes* (Germar, 1836)†

**Subfamily STAPHYLININAE Latreille, 1802**

**Tribe Diochini Casey, 1906**

*Diochus schaumi* Kraatz, 1860

**Tribe Othiini C.G. Thomson, 1859**

*Atrecus americanus* (Casey, 1906)

*Atrecus macrocephalus* (Nordmann, 1837)

**Tribe Staphylinini Latreille, 1802**

Subtribe Amblyopinina Seevers, 1944

*Heterothops fusculus* LeConte, 1863

*Heterothops minor* Smetana, 1971

*Heterothops pusio* LeConte, 1863

Subtribe Anisolinina Hayashi, 1993

*Tympanophorus puncticollis* (Erichson, 1840)

Subtribe Philonthina Kirby, 1837

*Bisnius blandus* (Gravenhorst, 1806)

*Bisnius cephalicus* (Casey, 1915)

***Bisnius fimetarius* (Gravenhorst, 1802)**†

*Bisnius palmi* (Smetana, 1955)

***Bisnius pugetensis* (Hatch, 1957)**

*Bisnius quediinus* (Horn, 1884)

*Bisnius siegwaldii* (Mannerheim, 1843)

*Bisnius sordidus* (Gravenhorst, 1802)†

Cafius (Euremus) bistriatus (Erichson, 1840)

*Erichsonius alumnus* Frank, 1975

*Erichsonius brachycephalus* Frank, 1975

*Erichsonius inutilis* (Horn, 1884)

*Erichsonius nanus* (Horn, 1884)

*Erichsonius parcus* (Horn, 1884)

*Erichsonius patella* (Horn, 1884)

*Erichsonius pusio* (Horn, 1884)

*Erichsonius rosellus* Frank, 1975

*Gabrius appendiculatus* Sharp, 1910†

*Gabrius austutoides* (Strand, 1946)†

*Gabrius brevipennis* (Horn, 1884)

*Gabrius fallaciosus* (Horn, 1884)

***Gabrius lysippus* Smetana, 1995**

*Gabrius microphthalmus* (Horn, 1884)

*Gabrius picipennis* (Mäklin, 1852)

*Gabrius ulpius* Smetana, 1995

*Hesperus apicialus* (Say, 1830)

*Laetulonthus laetulus* (Say, 1830)

*Neobisnius jucundus* (Horn, 1884)

*Neobisnius lathrobioides* (Baudi, 1848)†

*Neobisnius sobrinus* (Erichson, 1840)

*Neobisnius terminalis* (LeConte, 1863)

*Neobisnius villosulus* (Stephens, 1833)†

*Philonthus aequalis* Horn, 1884

*Philonthus boreas* Smetana, 1995

*Philonthus caeruleipennis caeruleipennis* (Mannerheim, 1830)

*Philonthus carbonarius* (Gravenhorst, 1802)†

*Philonthus cognatus* Stephens, 1832†

*Philonthus concinnus* (Gravenhorst, 1802)†

*Philonthus couleensis* Hatch, 1857

*Philonthus cruentatus* (Gmelin, 1790)†

*Philonthus debilis* (Gravenhorst, 1802)†

*Philonthus discoideus* (Gravenhorst, 1802)†

*Philonthus flavibasis* Casey, 1915

*Philonthus flumineus* Casey, 1915

*Philonthus furvus* Nordmann, 1837

*Philonthus fusiformis* Melsheimer, 1844

*Philonthus gracilior* Casey, 1915

*Philonthus hepaticus* Erichson, 1840

*Philonthus hudsonicus* Horn, 1884

*Philonthus janus* Smetana, 1995

*Philonthus jurgans* Tottenham, 1937†

*Philonthus lindrothi* Smetana, 1965

*Philonthus lomatus* Erichson, 1840

*Philonthus monaeses* Smetana, 1995

*Philonthus neonatus* Smetana, 1965

*Philonthus opacipennis* Notmann, 1919

*Philonthus palliatus* (Gravenhorst, 1806)

*Philonthus politus* (Linnaeus, 1758)†

*Philonthus pseudolodes* Smetana, 1996

*Philonthus quadricollis* Horn, 1884

*Philonthus rectangulus* Sharp, 1874†

*Philonthus schwarzi* Horn, 1884

*Philonthus sericans* (Gravenhorst, 1802)

*Philonthus sericinus* Horn, 1884

*Philonthus sessor* Smetana, 1965

*Philonthus sphagnorum* Smetana, 1995

*Philonthus spiniformis* Hatch, 1957

*Philonthus strictus* Hausen, 1891

*Philonthus subvirescens* C.G. Thomson, 1884*

*Philonthus thoracicus* (Gravenhorst, 1802)

*Philonthus umbratilis* (Gravenhorst, 1802)†

*Philonthus umbrinoides* Smetana, 1995

*Philonthus validus* Casey, 1915

*Philonthus varians* (Paykull, 1789)†

*Philonthus varro* Smetana, 1995

*Philonthus vulgatus* Casey, 1915

Subtribe Quediina Kraatz, 1857

Acylophorus (Acylophorus) caseyi Leng, 1920

Acylophorus (Acylophorus) pronus Erichson, 1840

Acylophorus (Amacylophorus) pratensis LeConte, 1863

*Anaquedius vernix* (LeConte, 1878)

*Hemiquedius ferox* (LeConte, 1878)

Quedius (Distichalius) capucinus (Gravenhorst, 1806)

Quedius (Distichalius) cinctus (Paykull, 1790)†

Quedius (Microsaurus) bicoloris Smetana & Webster, 2011

Quedius (Microsaurus) campbelli Smetana, 1971

Quedius (Microsaurus) canadensis (Casey, 1915)

Quedius (Microsaurus) criddlei (Casey, 1915)

Quedius (Microsaurus) erythrogaster Mannerheim, 1852

Quedius (Microsaurus) mesomelinus mesomelinus (Marsham, 1802)†

Quedius (Microsaurus) peregrinus (Gravenhorst, 1806)

Quedius (Microsaurus) spelaeus spelaeus Horn, 1871

Quedius (Quedionuchus) plagiatus Mannerheim, 1843*

Quedius (Quedius) curtipennis Bernhauer, 1908†

Quedius (Quedius) labradorensis labradorensis Smetana, 1965

Quedius (Raphirus) frigidus Smetana, 1971

Quedius (Raphirus) fulvicollis (Stephens, 1833)*

Quedius (Raphirus) rusticus Smetana, 1971

Quedius (Raphirus) simulator Smetana, 1971

Subtribe Staphylinina Latreille, 1802

*Creophilus maxillosus villosus* (Gravenhorst, 1802)

Dinothenarus (Dinothenarus) capitatus Bland, 1864

Dinothenarus (Parabemus) badipes (LeConte, 1863)

*Ontholestes cingulatus* (Gravenhorst, 1802)

*Platydracus cinnamopterus* (Gravenhorst, 1802)

*Platydracus fossator* (Gravenhorst, 1802)

*Platydracus violaceus* (Gravenhorst, 1802)

*Platydracus viridanus* (Horn, 1879)

*Staphylinus ornaticauda* LeConte, 1863

Tasgius (Rayacheila) melanarius melanarius (Heer, 1839)†

Tasgius (Tasgius) ater (Gravenhorst, 1802)†

**Tribe Xantholinini Erichson, 1839**

*Gyrohypnus angustatus* Stephens, 1833†

*Gyrohypnus campbelli* Smetana, 1982

*Gyrohypnus fracticornis* (O.F. Müller, 1776)†

*Hypnogyra gularis* (LeConte, 1880)

*Leptacinus intermedius* Donisthorpe, 1936†

*Neohypnus beckeri* Smetana, 1982

*Neohypnus hamatus* (Say, 1830)

*Neohypnus obscurus* (Erichson, 1839)

*Nudobius cephalus* (Say, 1830)

*Oxybleptes kiteleyi* Smetana, 1982

*Phacophallus parumpunctatus* (Gyllenhal, 1827)†

*Stictolinus flavipes* (LeConte, 1863)

Xantholinus (Xantholinus) linearis linearis (Olivier, 1795)†

*Xestolinus abdominalis* Casey, 1906

**Superfamily SCARABAEOIDEA Latreille, 1802**

**Family GEOTRUPIDAE Latreille, 1802**

(Earth-boring scarab beetles)

**Subfamily BOLBOCERATINAE Mulsant, 1852**

**Tribe Odonteini Shokhin, 2007**

*Odonteus liebecki* Wallis, 1928

**Subfamily GEOTRUPINAE Latreille, 1802**

**Tribe Geotrupini Latreille, 1802**

Geotrupus (Anoplotrupes) balyi Jekel, 1865

Geotrupus (Cnemotrupes) splendidus splendidus (Fabricius, 1775)

Geotrupus (Geotrupus) stercorarius (Linnaeus, 1758)†

**Family TROGIDAE MacLeay, 1819**

(Hide beetles)

**Subfamily TROGINAE MacLeay, 1819**

*Trox aequalis* Say, 1832

*Trox scaber* (Linnaeus, 1767)

*Trox unistriatus* Palisot de Beauvois, 1818

*Trox variolatus* Melsheimer, 1845

**Family LUCANIDAE Latreille, 1804**

(Stag beetles)

**Subfamily AESALINAE MacLeay, 1819**

**Tribe Nicagini LeConte, 1861**

*Nicagus obscurus* (LeConte, 1847)

**Subfamily SYNDESINAE MacLeay, 1819**

*Ceruchus piceus* (Weber, 1801)

**Subfamily LUCANINAE Latreille, 1804**

**Tribe Platycerinae Mulsant, 1842**

*Platycerus depressus* LeConte, 1850

*Platycerus virescens* (Fabricius, 1775)

**Family SCARABAEIDAE Latreille, 1802**

(Scarab beetles)

**Subfamily AEGIALIINAE Laporte, 1840**

Aegialia (Aegialia) opifex Horn, 1887

Aegialia (Psammoporus) criddlei W.J. Brown, 1931

Aegialia (Psammoporus) lacustris LeConte, 1850

Aegialia (Psammoporus) nana W.J. Brown, 1931

*Caelius humeralis* (W.J. Brown, 1931)

*Caelius rufescens* (Horn, 1887)

**Subfamily APHODIINAE Leach, 1815**

**Tribe Aphodiini Leach, 1815**

Subtribe Aphodiina Leach, 1815

*Acrossus rubripennis* (Horn, 1870)†

***Agoliinus guttatus* (Eschscholtz, 1823)**

*Agoliinus leopardus* (Horn, 1870)

*Agoliinus manitobensis* (W.J. Brown, 1928)

*Aphodius fimetarius* (Linnaeus, 1758)†

*Calomosternus granarius* (Linnaeus, 1767)†

***Chilothorax distinctus* (O.F. Müller, 1776)**†

*Colobopterus erraticus* (Linnaeus, 1758)†

*Dialytellus dialytoides* (Fall, 1907)

*Dialytes striatulus* (Say, 1825)

***Dialytes ulkei* Horn, 1875**

*Diapterna hyperborea* (LeConte, 1850)

*Diapterna omissa* (LeConte, 1850)

*Diapterna pinguis* (Haldeman, 1848)

*Eupleurus subterraneus* (Linnaeus, 1758)†

*Melinopterus prodromus* (Brahm, 1790)†

*Oscarinus rusicola* (Melsheimer, 1845)

*Optophorus haemorrhoidalis* (Linnaeus, 1758)†

*Planolinellus vittatus* (Say, 1825)†

*Planolinoides aenictus* (Cooper & Gordon, 1987)

*Planolinoides borealis* (Gyllenhal, 1827)*

***Planolinus tenellus* (Say, 1823)**

*Stenothorax badipes* (Melsheimer, 1845)

*Teuchestes fossor* (Linnaeus, 1758)†

*Trichonotulus scrofa* (Fabricius, 1787)†

**Tribe Eupariini Schmidt, 1910**

*Ataenius abditus* (Haldeman, 1848)

*Ataenius strigatus* (Say, 1823)

**Subfamily SCARABAEINAE Latreille, 1802**

**Tribe Onthophagini Burmeister, 1846**

Onthophagus (Onthophagus) hecate hecate (Panzer, 1794)

Onthophagus (Onthophagus) nuchicornis (Linnaeus, 1758)†

**Subfamily MELOLONTHINAE Leach, 1819**

**Tribe Dichelonychini Burmeister, 1855**

*Dichelonyx albicollis* Burmeister, 1855

*Dichelonyx diluta* Fall, 1901

*Dichelonyx elongatula* (Schönherr, 1817)

*Dichelonyx subvittata* LeConte, 1856

**Tribe Diplotaxini Kirby, 1837**

*Diplotaxis tristis* Kirby, 1837

**Tribe Hopliini Latreille, 1829**

Subtribe Hopliina Latreille, 1829

*Hoplia trifasciata* Say, 1825

**Tribe Melolonthini Leach, 1819**

Subtribe Melolonthina Leach, 1819

*Phyllophaga anxia* (LeConte, 1850)

*Phyllophaga drakii* (Kirby, 1837)

*Phyllophaga futilis* (LeConte, 1850)

Subtribe Rhizotrogina Burmeister, 1855

***Amphimallon majale* (Razoumowsky, 1789)**†

**Tribe Sericini Kirby, 1837**

Subtribe Sericina Kirby, 1837

*Serica atracapilla* (Kirby, 1837)

*Serica georgiana lecontei* Dawson, 1921

*Serica tristis* LeConte, 1850

**Subfamily RUTELINAE MacLeay, 1819**

**Tribe Anomalini Struebel, 1839**

Subtribe Popilliina Ohaus, 1918

*Popillia japonica* Newman, 1838†

**Subfamily DYNASTINAE MacLeay, 1819**

**Tribe Pentodontini Mulsant, 1842**

*Tomarus relictus* (Say, 1825)

**Subfamily CETONIINAE Leach, 1815**

**Tribe Cremastocheilini Burmeister, 1841**

Subtribe Cremastocheilina Burmeister, 1841

Cremastocheilus (Cremastocheilus) castaneae castaneae Knoch, 1801

**Tribe Trichiini Fleming, 1821**

Subtribe Osmodermatina Schenkling, 1922

*Osmoderma eremicola* (Knoch, 1801)

*Osmoderma scabra* (Palisot de Beauvois, 1805)

Subtribe Trichiina Fleming, 1821

*Gnorimella maculosa* (Knoch, 1801)

*Trichiotinus assimilis* (Kirby, 1837)

**Superfamily SCIRTOIDEA Fleming, 1821**

**Family EUCINETIDAE Lacordaire, 1857**

(Plate-thigh beetles)

*Eucinetus haemorrhoidalis* (Germar, 1818)†

*Eucinetus morio* LeConte, 1853

*Nycteus oviformis* (LeConte, 1866)

*Nycteus punctulatus* (LeConte, 1875)

*Nycteus testaceus* (LeConte, 1866)

**Family CLAMBIDAE Fischer von Waldheim, 1821**

(Minute beetles)

**Subfamily CLAMBINAE Fischer von Waldheim, 1821**

*Clambus armadillo armadillo* (DeGeer, 1774)†

*Clambus howdeni* Endrödy-Younga, 1981

**Family SCIRTIDAE Fleming, 1821**

(Marsh beetles)

**Subfamily SCIRTINAE Fleming, 1821**

*Cyphon collaris* (Guérin-Méneville, 1843)

*Cyphon confusus* W.J. Brown, 1930

*Cyphon neovariabilis* Klausnitzer, 1976

*Cyphon obscurus* (Guérin-Méneville, 1843)

*Cyphon padi* (Linnaeus, 1758)†

*Cyphon ruficollis* (Say, 1825)

*Cyphon variabilis* (Thunberg, 1785)*

*Elodes maculicollis* Horn, 1880

*Microcara explanata* (LeConte, 1866)

*Prionocyphon discoideus* (Say, 1825)

*Prionocyphon limbatus* LeConte, 1866

*Sacodes pulchella* (Guérin-Méneville, 1843)

***Sacodes thoracica* (Guérin-Méneville, 1843)**

*Sarabandus robustus* (LeConte, 1875)

*Scirtes tibialis* Guérin-Méneville, 1843

**Superfamily BUPRESTOIDEA Leach, 1815**

**Family BUPRESTIDAE Leach, 1815**

(Metallic wood-boring beetles)

**Subfamily CHRYSOCHROINAE Laporte, 1835**

**Tribe Chrysochroini Laporte, 1835**

Subtribe Chalcophorina Lacordaire, 1857

*Chalcophora fortis* LeConte, 1860

*Chalcophora liberata* (Germar, 1824)

*Chalcophora virginiensis* (Drury, 1770)

**Tribe Dicercini Gistel, 1848**

Subtribe Dicercina Gistel, 1848

***Dicerca callosa callosa* Casey, 1909**

*Dicerca caudata* LeConte, 1860

*Dicerca divaricata* (Say, 1823)

*Dicerca lugubris* LeConte, 1860

*Dicerca punctulata* (Schönherr, 1817)

*Dicerca tenebrica* (Kirby, 1837)

*Dicerca tenebrosa* (Kirby, 1837)

*Dicerca tuberculata* (Laporte & Gory, 1837)

**Tribe Poecilonotini Jakobson, 1913**

Subtribe Poecilonotina Jakobson, 1913

*Poecilonota cyanipes* (Say, 1823)

***Poecilonota ferrea* (Melsheimer, 1845)**

**Subfamily BUPRESTINAE Leach, 1815**

**Tribe Anthaxiini Gory & Laporte, 1839**

Anthaxia (Haplanthaxia) quercata (Fabricius, 1801)

**Anthaxia (Haplanthaxia) viridifrons Gory, 1841**

Anthaxia (Melanthaxia) inornata (Randall, 1838)

**Tribe Buprestini Leach, 1815**

Subtribe Buprestina Leach, 1815

Buprestis (Buprestis) consularis Gory, 1840

Buprestis (Buprestis) maculativentris Say, 1824

Buprestis (Cypriacis) striata (Fabricius, 1775)

Buprestis (Cypriacis) sulcicollis (LeConte, 1860)

Buprestis (Nelsonocheira) fasciata (Fabricius, 1787)

**Tribe Chrysobothrini Gory & Laporte, 1836**

*Chrysobothris dentipes* (Germar, 1824)

*Chrysobothris femorata* (Olivier, 1790)

*Chrysobothris harrisi* (Hentz, 1827)

*Chrysobothris neopusilla* Fisher, 1942

*Chrysobothris pusilla* Gory & Laporte, 1837

*Chrysobothris rotundicollis* Gory & Laporte, 1837

*Chrysobothris scabripennis* Gory & Laporte, 1837

*Chrysobothris sexsignata* (Say, 1833)

*Chrysobothris trinervia* (Kirby, 1837)

*Chrysobothris verdigripennis* Frost, 1910

**Tribe Melanophilini Bedel, 1921**

*Melanophila acuminata* (DeGeer, 1774)*

*Phaenops abies* (Champlain & Knull, 1923)

*Phaenops aeneola* (Melsheimer, 1845)

*Phaenops drummondi* (Kirby, 1837)

*Phaenops fulvoguttata* (Harris, 1829)

**Subfamily AGRILINAE Laporte, 1835**

**Tribe Agrilini Laporte, 1835**

Subtribe Agrilina Laporte, 1835

*Agrilus anxius* Gory, 1841

*Agrilus arcuatus* (Say, 1825)

*Agrilus bilineatus* (Weber, 1801)

*Agrilus crataegi* Frost, 1912

*Agrilus criddlei* Frost, 1920

*Agrilus crinicornis* Horn, 1891

*Agrilus cuprescens cuprescens* (Ménétriés, 1832)†

*Agrilus granulatus liragus* Barter & W.J. Brown, 1950

*Agrilus horni* Kerremans, 1900

***Agrilus juglandis* Knull, 1920**

***Agrilus masculinus* Horn, 1891**

*Agrilus obsoletoguttatus* Gory, 1841

***Agrilus osburni* Knull, 1937**

*Agrilus pensus* Horn, 1891

*Agrilus politus* (Say, 1825)

***Agrilus pseudocoryli* Fisher, 1928**

*Agrilus putillus putillus* Say, 1834

*Agrilus ruficollis* (Fabricius, 1787)

*Agrilus sayi* Saunders, 1870

**Tribe Coraebini Bedel, 1921**

Subtribe Coraebina Bedel, 1921

*Eupristocerus cogitans* (Weber, 1801)

**Tribe Tracheini Laporte, 1835**

Subtribe Brachina LeConte, 1861

*Brachys aerosus* Melsheimer, 1845

*Brachys ovatus* (Weber, 1801)

*Taphrocerus gracilis* (Say, 1825)

**Superfamily BYRRHOIDEA Latreille, 1804**

**Family BYRRHIDAE Latreille, 1804**

(Pill beetles)

**Subfamily BYRRHINAE Latreille, 1804**

**Tribe Byrrhini Latreille, 1804**

*Byrrhus americanus* LeConte, 1850

*Byrrhus cyclophorus* Kirby, 1837

*Byrrhus geminatus* LeConte, 1854*

*Byrrhus kirbyi* LeConte, 1854

*Cytilus alternatus* (Say, 1825)

*Porcinolus undatus* (Melsheimer, 1844)

**Subfamily SYNCALYPTINAE Mulsant & Rey, 1869**

**Tribe Syncalyptini Mulsant & Rey, 1869**

*Chaetophora spinosa* (Rossi, 1794)†

**Family ELMIDAE Curtis, 1830**

(Riffle beetles)

**Subfamily ELMINAE Curtis, 1830**

**Tribe Elmini Curtis, 1830**

***Dubiraphia minima* Hilsenhoff, 1973**

*Dubiraphia quadrinotata* (Say, 1825)

***Dubiraphia vittata* (Melsheimer, 1844)**

*Microcylloepus pusillus pusillus* (LeConte, 1852)

*Optioservus fastiditus* (LeConte, 1850)

*Optioservus ovalis* (LeConte, 1863)

*Optioservus trivittatus* (W.J. Brown, 1930)

*Oulimnius latiusculus* (LeConte, 1866)

*Promoresia elegans* (LeConte, 1852)

*Promoresia tardella* (Fall, 1925)

*Stenelmis crenata* (Say, 1824)

*Stenelmis mera* Sanderson, 1938

*Macronychus glabratus* Say, 1825

**Family DRYOPIDAE Billberg, 1820**

(Long-toed water beetles)

*Dryops viennensis* (Laporte, 1840)†

*Helichus basalis* LeConte, 1852

*Helichus striatus* LeConte, 1852

**Family LIMNICHIDAE Erichson, 1846**

(Minute marsh-loving beetles)

**Subfamily LIMNICHINAE Erichson, 1846**

**Tribe Limnichini Erichson, 1846**

***Limnichites punctatus* (LeConte, 1854)**

**Family HETEROCERIDAE MacLeay, 1825**

(Variegated mud-loving beetles)

**Subfamily HETEROCERINAE MacLeay, 1825**

**Tribe Heterocerini MacLeay, 1825**

*Heterocerus fatuus* Kiesenwetter, 1851

*Heterocerus pallidus* Say, 1823

*Heterocerus parrotus* Pacheco, 1964

***Heterocerus subtilis* W.V. Miller, 1988**

*Heterocerus tristis* Mannerheim, 1853

*Heterocerus undatus* Melsheimer, 1844

**Family PSEPHENIDAE Lacordaire, 1854**

(Water-penny beetles)

**Subfamily EUBRIINAE Lacordaire, 1857**

*Ectopria nervosa* (Melsheimer, 1845)

*Ectopria thoracica* (Ziegler, 1845)

**Subfamily PSEPHENINAE Lacordaire, 1854**

*Psephenus herricki* (DeKay, 1844)

**Family PTILODACTYLIDAE Laporte, 1836**

(Toed-winged beetles)

**Subfamily ANCHYTARSINAE Champion, 1897**

*Anchytarsus bicolor* (Melsheimer, 1845)

**Subfamily PTILODACTYLINAE Laporte, 1836**

***Ptilodactyla carinata* Johnson & Freytag, 1978**

**Superfamily ELATEROIDEA Leach, 1815**

**Family ARTEMATOPODIDAE Lacordaire, 1857**

(Artematopodid beetles)

**Subfamily ARTEMATOPODINAE Lacordaire, 1857**

*Macropogon piceus* LeConte, 1861

**Family EUCNEMIDAE Eschscholtz, 1829**

(False click beetles)

**Subfamily MELASINAE Fleming, 1821**

**Tribe Dirhagini Reitter, 1911**

*Entomophthalmus rufiolus* (LeConte, 1866)

*Microrhagus pectinatus* LeConte, 1866

*Microrhagus subsinuatus* LeConte, 1852

*Microrhagus triangularis* (Say, 1823)

***Sarpedon scabrosus* Bonvouloir, 1875**

**Tribe Epiphanini Muona, 1993**

*Epiphanis cornutus* Eschscholtz, 1829

*Hylis terminalis* (LeConte, 1866)

***Dirrhagofarsus ernae* Otto, Muona & Mcclarin, 2014**†

**Tribe Melasini Fleming, 1821**

Subtribe Melasina Fleming, 1821

*Isorhipis obliqua* (Say, 1839)

*Isorhipis ruficornis* (Say, 1823)

**Tribe Xylobiini Reitter, 1911**

*Xylophilus cylindriformis* (Horn, 1871)

**Subfamily EUCNEMINAE Eschscholtz, 1829**

**Tribe Mesogenini Muona, 1993**

*Stethon pectorosus* LeConte, 1866

**Subfamily MACRAULACINAE Fleutiaux, 1923**

**Tribe Macraulacini Fleutiaux, 1923**

*Deltometopus amoenicornis* (Say, 1839)

*Dromaeolus harringtoni* Horn, 1886

***Isarthrus calceatus* (Say, 1839)**

*Isarthrus rufipes* (Melsheimer, 1844)

*Onichodon canadensis* (W.J. Brown, 1940)

*Onichodon orchesides* Newman, 1838

**Tribe Nematodini Leiler, 1976**

*Nematodes penetrans* (LeConte, 1852)

**Family THROSCIDAE Laporte, 1840**

(Throscid beetles)

*Aulonothroscus constrictor* (Say, 1839)

*Trixagus carnicollis* (C. Schaeffer, 1916)

***Trixagus chevrolati* (Bonvouloir, 1859)**

**Family ELATERIDAE Leach, 1815**

(Click beetles)

**Subfamily AGRYPNINAE Candèze, 1857**

**Tribe Agrypnini Candèze, 1857**

*Danosoma brevicorne* (LeConte, 1853)

*Danosoma obtectum* (Say, 1839)

*Lacon auroratus* (Say, 1839)

***Lacon maculatus* (LeConte, 1866)**

**Subfamily LISSOMINAE Laporte, 1835**

*Oestodes tenuicollis* (Randall, 1838)

**Subfamily PITYOBIINAE Hyslop, 1917**

*Pityobius anguinus* LeConte, 1853

**Subfamily DENDROMETRINAE Gistel, 1848**

**Tribe Dendrometrini Gistel, 1848**

Subtribe Dendrometrina Gistel, 1848

*Athous acanthus* (Say, 1839)

*Athous brightwelli* (Kirby, 1837)

*Athous campyloides* Newman, 1833†

***Athous equestris* (LeConte, 1853)**

*Athous fossularis* (LeConte, 1853)

*Athous orvus* Becker, 1974

*Athous posticus* (Melsheimer, 1845)

*Athous productus* (Randall, 1838)

*Athous rufifrons* (Randall, 1838)

*Athous scapularis* (Say, 1839)

*Elathous discalceatus* (Say, 1834)

*Limonius aeger* LeConte, 1853

***Limonius aurifer* LeConte, 1853**

*Limonius confusus* LeConte, 1853

*Limonius pectoralis* LeConte, 1866

***Limonius stigma* (Herbst, 1806)**

*Limonius subauratus* LeConte, 1853

Subtribe Denticollina Stein & Weise, 1877

*Denticollis denticornis* (Kirby, 1837)

Subtribe Hemicrepidiina Champion, 1896

***Harminius triundulatus* (Mannerheim, 1853)**

*Hemicrepidius brevicollis* (Candèze, 1863)

*Hemicrepidius hemipodus* (Say, 1825)

*Hemicrepidius memnonius* (Herbst, 1806)

*Hemicrepidius niger* (Linnaeus, 1758)†

**Tribe Hypnoidini Schwarz, 1906**

*Hypnoidus abbreviatus* (Say, 1823)

*Hypnoidus bicolor* (Eschscholtz, 1829)*

*Hypnoidus rivularius* (Gyllenhal, 1827)*

*Ligmargus lecontei* (Leng, 1918)

Margaiostus (Margaiostus) grandicollis (LeConte, 1863)

**Tribe Prosternini Gistel, 1856**

*Actenicerus cuprascens* LeConte, 1853

*Anostirus vernalis* (Hentz, 1827)

*Beckerus appressa* (Randall, 1838)

*Corymbitodes dorothyae* (Knull, 1959)

*Corymbitodes elongaticollis* (Hamilton, 1893)

*Corymbitodes pygmaeus* (Van Dyke, 1932)

*Corymbitodes tarsalis* (Melsheimer, 1845)

*Ctenicera kendalli* Kirby, 1837

Eanus (Eanus) estriatus (LeConte, 1853)

Eanus (Eanus) maculipennis LeConte, 1863

**Eanus (Paranomus) decoratus (Mannerheim, 1853)**

*Hypoganus rotundicollis* (Say, 1825)

*Hypoganus sulcicollis* (Say, 1833)

*Liotrichus falsificus* (LeConte, 1853)

*Liotrichus spinosus* (LeConte, 1853)

*Liotrichus vulneratus* (LeConte, 1863)

*Metanomus insidiosus* (LeConte, 1853)

*Nitidolimonius resplendens* (Eschscholtz, 1829)

*Oxygonus montanus* C. Schaeffer, 1917

*Oxygonus obesus* (Say, 1823)

*Paractenicera fulvipes* (Bland, 1863)

*Prosternon medianum* (Germar, 1843)

*Pseudanostirus hamatus* (Say, 1834)

*Pseudanostirus hieroglyphicus* (Say, 1834)

*Pseudanostirus nigricollis* (Bland, 1864)

*Pseudanostirus propolus propolus* (LeConte, 1853)

*Pseudanostirus triundulatus* (Randall, 1838)

*Selatosomus appropinquans* (Randall, 1838)

*Selatosomus pulcher* (LeConte, 1853)

*Selatosomus splendens* (Ziegler, 1844)

*Setasomus aratus* (LeConte, 1853)

*Setasomus nitidulus* (LeConte, 1853)

*Setasomus rufopleuralis* (Fall, 1933)

*Sylvanelator cylindriformis* (Herbst, 1806)

**Subfamily NEGASTRIINAE Nakane & Kishii, 1956**

**Tribe Negastriini Nakane & Kishii, 1956**

*Microhypnus striatulus* (LeConte, 1853)

*Negastrius arnetti* Stibick, 1991

*Negastrius atrosus* Wells, 1996

*Negastrius delumbis* (Horn, 1891)

*Neohypdonus tumescens* (LeConte, 1853)

*Oedostethus femoralis* LeConte, 1853

*Paradonus olivereae* Stibick, 1991

*Paradonus pectoralis* (Say, 1834)

*Zorochros melsheimeri* (Horn, 1891)

**Subfamily ELATERINAE Leach, 1815**

**Tribe Agriotini Laporte, 1840**

Subtribe Agriotina Laporte, 1840

*Agriotes collaris* (LeConte, 1853)

*Agriotes fucosus* (LeConte, 1853)

*Agriotes limosus* (LeConte, 1853)

*Agriotes mancus* (Say, 1823)

*Agriotes pubescens* Melsheimer, 1845

*Agriotes quebecensis* W.J. Brown, 1933

*Agriotes sputator* (Linnaeus, 1758)†

*Agriotes stabilis* (LeConte, 1853)

*Dalopius agnellus* W. J. Brown, 1934

*Dalopius brevicornis* W. J. Brown, 1934

*Dalopius cognatus* W. J. Brown, 1934

*Dalopius fuscipes* W. J. Brown, 1934

*Dalopius gentilus* W. J. Brown, 1934

*Dalopius pallidus* W. J. Brown, 1934

*Dalopius vagus* W. J. Brown, 1934

**Tribe Ampedini Gistel, 1848**

*Ampedus apicatus* (Say, 1834)

*Ampedus areolatus* (Say, 1823)

*Ampedus collaris* (Say, 1825)

*Ampedus deletus* (LeConte, 1853)

*Ampedus evansi* W.J. Brown, 1933

*Ampedus fusculus* (LeConte, 1853)

*Ampedus laurentinus* W.J. Brown, 1933

***Ampedus linteus* (Say, 1839)**

*Ampedus luctuosus* (LeConte, 1853)

*Ampedus miniipennis* (LeConte, 1853)

*Ampedus mixtus* (Herbst, 1806)

*Ampedus molestus* (LeConte, 1853)

*Ampedus nigricans* Germar, 1844

*Ampedus nigricollis* (Herbst, 1806)

*Ampedus nigrinus* (Herbst, 1784)*

*Ampedus oblessus* (Say, 1833)

*Ampedus protervus* (LeConte, 1853)

*Ampedus pullus* Germar, 1844

*Ampedus rubricus* (Say, 1825)

*Ampedus sanguinipennis* (Say, 1823)

*Ampedus sayi* (LeConte, 1853)

*Ampedus semicinctus* (Randall, 1838)

*Ampedus vitiosus* (LeConte, 1853)

**Tribe Elaterini Leach, 1815**

*Elator abruptus* Say, 1825

*Sericus honestus* (Randall, 1838)

*Sericus incongruus* (LeConte, 1853)

*Sericus viridanus* (Say, 1825)

**Tribe Megapenthini Gurjeva, 1973**

*Megapenthes rogersi* Horn, 1871

*Megapenthes solitarius* Fall, 1934

*Megapenthes stigmosus* (LeConte, 1853)

**Tribe Melanotini Candèze, 1859**

*Melanotus castanipes* (Paykull, 1800)‡

*Melanotus decumanus* (Erichson, 1841)

*Melanotus leonardi* (LeConte, 1853)

*Melanotus sagittarius* (LeConte, 1853)

*Melanotus similis* (Kirby, 1837)

**Tribe Pomachiliini Candèze, 1859**

*Idolus bigeminata* (Randall, 1838)

*Idolus debilis* (LeConte, 1884)

**Subfamily CARDIOPHORINAE Candèze, 1859**

*Cardiophorus convexulus* LeConte, 1853

*Cardiophorus gagates* Erichson, 1840

*Cardiophorus propinquus* Lanchester, 1971

**Family LYCIDAE Laporte, 1836**

(Net-winged beetles)

**Subfamily DICTYOPTERINAE Houlbert, 1922**

**Tribe Dictyopterini Houlbert, 1922**

*Dictyoptera aurora* (Herbst, 1784)*

*Greenarus thoracicus* (Randall, 1838)

**Subfamily LYCINAE Laporte, 1836**

**Tribe Calochromini Lacordaire, 1857**

*Calochromus perfacetus* (Say, 1825)

**Tribe Calopterini Green, 1949**

Subtribe Calopterina Green, 1949

*Caenia dimidiata* (Fabricius, 1801)

*Calopteron terminale* (Say, 1823)

*Leptoceletes basalis* (LeConte, 1847)

**Tribe Erotini LeConte, 1881**

*Eropterus arculus* Green, 1951

*Eros humeralis* (Fabricius, 1801)

Erotides (Erotides) sculptilis (Say, 1835)

Lopheros (Lopheros) crenatus (Germar, 1824)

Lopheros (Lopheros) fraternus (Randall, 1838)

**Tribe Platerodini Kleine, 1929**

*Plateros bispiculatus* Green, 1953

*Plateros flavoscutellatus* Blatchley, 1914

*Plateros lictor* (Newman, 1838)

*Plateros subfurcatus* Green, 1953

*Plateros volatus* Green, 1953

**Family LAMPYRIDAE Rafinesque, 1815**

(Fireflies)

**Subfamily LAMPYRINAE Rafinesque, 1815**

**Tribe Cratomorphini Green, 1948**

*Pyractomena angulata* (Say, 1825)

*Pyractomena borealis* (Randall, 1838)

*Pyractomena linearis* LeConte, 1851

**Tribe Lucidotini Lacordaire, 1857**

Subtribe Lucidotina Lacordaire, 1857

*Lucidota atra* (Olivier, 1790)

Subtribe Photinina LeConte, 1881

Ellychnia (Ellychnia) corrusca (Linnaeus, 1767)

***Photinus aquilonius* Lloyd, 1969**

*Photinus ardens* LeConte, 1851

***Photinus ignitus* Fall, 1927**

*Photinus obscurellus* LeConte, 1851

*Pyropyga decipiens* (Harris, 1836)

*Pyropyga nigricans* (Say, 1823)

**Subfamily PHOTURINAE Lacordaire, 1857**

*Photuris fairchildi* Barber, 1951

**Family CANTHARIDAE Imhoff, 1856**

(Soldier beetles)

**Subfamily CANTHARINAE Imhoff, 1856**

**Tribe Cantharini Imhoff, 1856**

*Atalantycha bilineata* (Say, 1823)

*Cantharis livida* Linnaeus, 1758†

*Cantharis rufa* Linnaeus, 1758†

*Cantharis tuberculata* (LeConte, 1851)

*Pacificanthia curtisi* (Kirby, 1837)

*Pacificanthia rotundicollis* (Say, 1825)

***Rhagonycha dichroa* (LeConte, 1851)**

*Rhagonycha excavata* (LeConte, 1881)

*Rhagonycha fraxini* (Say, 1823)

*Rhagonycha fulva* (Scopoli, 1763)†

*Rhagonycha greeni* (Fall, 1936)

*Rhagonycha imbecillis* (LeConte, 1851)

*Rhagonycha luteicollis* (Germar, 1824)

*Rhagonycha mandibularis* (Kirby, 1837)

*Rhagonycha mollis mollis* Fall, 1936

*Rhagonycha nanula* (LeConte, 1881)

*Rhagonycha proxima* (Green, 1941)

*Rhagonycha recta* (Melsheimer, 1846)

*Rhagonycha scitula* (Say, 1825)

*Rhagonycha septentrionis* (Green, 1941)

***Rhagonycha sylvatica* (Green, 1941)**

***Rhagonycha tantilla* (LeConte, 1881)**

*Rhagonycha tenuis* (Green, 1940)

*Rhagonycha umbrina* (Green, 1841)

*Rhagonycha vilis* (LeConte, 1851)

*Rhaxonycha bilobata* (McKey-Fender, 1941)

*Rhaxonycha carolina* (Fabricius, 1801)

**Tribe Podabrini Gistel, 1856**

*Dichelotarsus cinctipennis* (LeConte, 1866)

*Dichelotarsus flavimanus* Motschulsky, 1860*

*Dichelotarsus laevicollis* (Kirby, 1837)

*Dichelotarsus limbellus* (LeConte, 1881)

*Dichelotarsus pattoni* (LeConte, 1866)

*Dichelotarsus piniphilus* (Eschscholtz, 1830)

*Dichelotarsus probus* (Fall, 1927)

*Dichelotarsus puberulus* (LeConte, 1850)

*Dichelotarsus punctatus* (LeConte, 1850)

*Dichelotarsus puncticollis* (Kirby, 1837)

*Dichelotarsus simplex* (Couper, 1865)

*Podabrus diadema* (Fabricius, 1798)

*Podabrus frosti* Fender, 1946

*Podabrus intrusus* Green, 1947

*Podabrus longicornis* Fall, 1928

*Podabrus modestus* (Say, 1823)

*Podabrus nothoides* LeConte, 1881

*Podabrus planulus* Green, 1947

*Podabrus rugosulus* LeConte, 1850

***Podabrus tricostatus* (Say, 1835)**

**Subfamily SILINAE Mulsant, 1862**

**Tribe Silini Mulsant, 1862**

*Polemius canadensis* W.J. Brown, 1940

*Polemius laticornis* (Say, 1825)

*Polemius repandus* LeConte, 1881

*Silis difficilis difficilis* LeConte, 1850

*Silis percomis* (Say, 1835)

**Subfamily MALTHININAE Kiesenwetter, 1852**

**Tribe Malthodini Böving & Craighead, 1930**

*Malthodes fragilis* (LeConte, 1851)

*Malthodes fuliginosus fuliginosus* LeConte, 1866

*Malthodes niger* (LeConte, 1851)

*Malthodes parvulus* (LeConte, 1851)

*Malthodes similis* Fender, 1951

**Subfamily CHAULIOGNATHINAE LeConte, 1861**

**Tribe Chauliognathini LeConte, 1861**

*Chauliognathus pensylvanicus* (DeGeer, 1774)

**Superfamily DERODONTOIDEA LeConte, 1861**

**Family DERODONTIDAE LeConte, 1861**

(Tooth-necked fungus beetles)

**Subfamily LARICOBIINAE Mulsant & Rey, 1864**

*Laricobius erichsonii* Rosenhauer, 1846†

*Laricobius rubidus* LeConte, 1861

**Superfamily BOSTRICHOIDEA Latreille, 1802**

**Family DERMESTDAE Latreille, 1804**

(Skin beetles)

**Subfamily DERMESTINAE Latreille, 1804**

**Tribe Dermestini Latreille, 1804**

Dermestis (Dermestis) lardarius Linnaeus, 1758†

Dermestis (Dermestis) pulcher LeConte, 1854

Dermestis (Dermestinus) talpinus Mannerheim, 1843

Dermestis (Dermestinus) undulatus Brahms, 1790†

**Subfamily ORPHILINAE LeConte, 1861**

*Orphilus ater* Erichson, 1846

**Subfamily ATTAGENINAE Laporte, 1840**

**Tribe Attagenini Laporte, 1840**

Attagenus (Attagenus) pellio (Linnaeus, 1758)†

Attagenus (Attagenus) unicolor japonicus Reitter, 1877)†

**Subfamily MEGATOMINAE Leach, 1815**

**Tribe Anthrenini Gistel, 1848**

Anthrenus (Anthrenus) scrophulariae scrophulariae (Linnaeus, 1758)†

Anthrenus (Florilinus) castaneae Melsheimer, 1844

Anthrenus (Florilinus) museorum (Linnaeus, 1761)†

Anthrenus (Helocerus) fuscus Olivier, 1789†

**Anthrenus (Nathrenus) verbasci (Linnaeus, 1767)** †

**Tribe Megatomini Leach, 1815**

**Megatoma (Perimegatoma) cylindrica (Kirby, 1837)**

***Trogoderma ornatum* (Say, 1825)**

*Trododerma sinistrum* Fall, 1926

**Family ENDECATOMIDAE LeConte, 1861**

*Endecatomus rugosus* (Randall, 1838)

**Family BOSTRICHIDAE Latreille, 1802**

(Bostrichid powder-post beetles)

**Subfamily DINODERINAE C.G. Thomson, 1863**

Dinoderus (Dinoderus) minutus (Fabricius, 1775)†

*Stephanopachys rugosus* (Olivier, 1795)

*Stephanopachys substriatus* (Paykull, 1800)*

**Subfamily LYCTINAE Billberg, 1820**

**Tribe Lyctini Billberg, 1820**

*Lyctus brunneus* (Stephens, 1830)†

*Lyctus linearis* (Goeze, 1777)†

***Lyctus planicollis* LeConte, 1858**

**Family PTINIDAE Latreille, 1892**

(Death watch and spider beetles)

**Subfamily EUCRADINAE LeConte, 1861**

**Tribe Eucradini LeConte, 1861**

***Eucrada humeralis* (Melsheimer, 1846)**

**Subfamily PTININAE Latreille, 1802**

**Tribe Gibbiini Jacquelin du Val, 1860**

*Gibbium aequinoctiale* Boieldieu, 1854†

**Tribe Meziini Bellés, 1985**

*Mezium affine* Boieldieu, 1856†

**Tribe Ptinini Latreille, 1802**

*Epauloecus unicolor* (Piller & Mitterpacher, 1783)†

*Niptus hololeucus* (Faldermann, 1835)†

*Pseudeurostus hilleri* (Reitter, 1877)†

Ptinus (Cyphoderes) raptor Sturm, 1837†

Ptinus (Ptinus) bicinctus Sturm, 1837†

Ptinus (Ptinus) fur (Linnaeus, 1758)†

Ptinus (Ptinus) latro Fabricius, 1775†

Ptinus (Tectoptinus) tectus Boieldieu, 1856†

**Subfamily ERNOBIINAE Pic, 1912**

***Ernobius filicornis* LeConte, 1879**

***Ernobius luteipennis* LeConte, 1879**

*Ernobius mollis* (Linnaeus, 1758)†

***Ernobius opicus* Fall, 1905**

***Ernobius schedli* W.J. Brown, 1932**

***Utobium elegans* (Horn, 1894)**

***Utobium granulatum* R.E. White, 1976**

***Xestobium gaspensis* R.E. White, 1975**

**Subfamily ANOBIINAE Fleming, 1821**

*Anobium punctatum* (DeGeer, 1774)†

*Hadrobregmus notatus* (Say, 1825)

*Hemicoelus carinatus* (Say, 1823)

***Hemicoelus defectus* (Fall, 1905)**

***Hemicoelus pusillus* (Fall, 1905)**

*Hemicoelus umbrosus* (Fall, 1905)

*Microbregma emarginatum* (Duftschmid, 1825)†

***Oligomerus alternans* LeConte, 1865**

***Oligomerus obtusus* LeConte, 1865**

***Platybregmus canadensis* Fisher, 1934**

*Priobium sericeum* (Say, 1825)

*Stegobium paniceum* (Linnaeus, 1758)†

**Subfamily PTILININAE Shuckard, 1839**

*Ptilinus lobatus* Casey, 1898

***Ptilinus pruinosus* Casey, 1898**

*Ptilinus ruficornis* Say, 1823

**Subfamily XYLETININAE Gistel, 1848**

**Tribe Lasiodermini Böving, 1927**

*Lasioderma serricorne* (Fabricius, 1792)†

*Euvrilletta harrisii* (Fall, 1905)

**Tribe Xyletinini Gistel, 1848**

***Vrilletta laurentina* Fall, 1905**

*Xyletinus fucatus* LeConte, 1865

***Xyletinus lugubris* LeConte, 1878**

**Subfamily DORCATOMINAE C.G. Thomson, 1859**

***Caenocara oculatum* (Say, 1824)**

***Dorcatoma falli* R.E. White, 1965**

*Dorcatoma pallicornis* LeConte, 1874

***Sculptotheca puberula* (LeConte, 1865)**

***Stagetus profundus* (LeConte, 1865)**

**Superfamily LYMEXYLOIDEA Fleming, 1821**

**Family LYMEXYLIDAE Fleming, 1821**

(Ship-timber beetles)

**Subfamily HYLECOETINAE Germar, 1818**

*Elateroides lugubris* (Say, 1835)

**Superfamily CLEROIDEA Latreille, 1802**

**Family TROGOSSITIDAE Latreille, 1802**

(Bark-gnawing beetles)

**Subfamily PELTINAE Latreille, 1806**

**Tribe Lophocaterini Crowson, 1964**

*Grynocharis quadrilineata* (Melsheimer, 1844)

**Tribe Peltini Latreille, 1806**

*Peltis fraterna* (Randall, 1838)

*Peltis septentrionalis* (Randall, 1838)

**Tribe Thymalini Léveillé, 1888**

*Thymalus marginicollis* Chevrolat, 1842

**Subfamily TROGOSSITINAE Latreille, 1802**

**Tribe Calityini Reitter, 1922**

*Calitys scabra* (Thunberg, 1784)*

**Tribe Trogossitini Latreille, 1802**

*Tenebriodes corticalis* (Melsheimer, 1844)

*Tenebriodes mauritanicus* (Linnaeus, 1758)‡

**Family CLERIDAE Latreille, 1802**

(Checkered beetles)

**Subfamily TILLINAE Fischer von Waldheim, 1813**

*Cymatodera bicolor* (Say, 1825)

***Cymatodera inornata* (Say, 1835)**

**Subfamily HYDNOCERINAE Spinola, 1844**

**Tribe Hydnocerini Spinola, 1844**

*Isohydnocera curtipennis* (Newman, 1840)

*Phyllobaenus humeralis* (Say, 1823)

*Phyllobaenus lecontei* (Wolcott, 1912)

*Phyllobaenus pallipennis* (Say, 1825)

*Phyllobaenus verticalis* (Say, 1835)

**Subfamily CLERINAE Latreille, 1802**

*Enoclerus muttkowskii* (Wolcott, 1909)

*Enoclerus nigripes nigripes* (Say, 1823)

*Enoclerus nigripes rufiventris* (Spinola, 1844)

*Thanasimus dubius* (Fabricius, 1777)

***Thanasimus trifasciatus* (Say, 1825)**

*Thanasimus undatulus nubilus* (Klug, 1842)

***Thanasimus undatulus undatulus* (Say, 1835)**

*Trichodes nutalli* (Kirby, 1818)

**Subfamily KORYNETINAE Laporte, 1836**

***Chariessa pilosa* (Forster, 1771)**

*Madoniella dislocata* (Say, 1825)

*Necrobia violacea* (Linnaeus, 1758)†

**Subfamily THANEROCLERINAE Chapin, 1924**

**Tribe Thaneroclerini Chapin, 1924**

*Zenodosus sanguineus* (Say, 1835)

**Family MELYRIDAE Leach, 1815**

(Soft-winged flower beetles)

**Subfamily DASYTINAE Laporte, 1840**

**Tribe Dasytini Laporte, 1840**

*Dolichosoma foveicolle* (Kirby, 1837)

***Hoppingiana hudsonica* (LeConte, 1866)**

**Subfamily MALACHIINAE Fleming, 1821**

**Tribe Malachiini Fleming, 1821**

**Attalus (Acletus) nigrellus (LeConte, 1852)**

Attalus (Attalus) morulus (LeConte, 1852)

**Attalus (Attalus) terminalis (Erichson, 1840)**

***Collops tricolor* (Say, 1823)**

*Collops vittatus* (Say, 1823)

Malachius (Malachius) aeneus (Linnaeus, 1761)†

*Nodopus flavilabris* (Say, 1825)

**Superfamily CUCUJOIDEA Latreille, 1802**

**Family BYTURIDAE Gistel, 1848**

(Fruitworm beetles)

**Subfamily BYTURINAE Gistel, 1848**

*Byturus unicolor* Say, 1823

**Family SPHINIDIDAE Jacquelin du Val, 1860**

(Cryptic slime mold beetles)

**Subfamily ODONTOSPHINDINAE Sen Gupta & Crowson, 1979**

*Odontosphindus denticollis* LeConte, 1878

**Subfamily SPHINDINAE Jacquelin du Val, 1860**

*Eurysphindus hirtus* LeConte, 1878

*Sphindus americanus* LeConte, 1866

*Sphindus trinifer* Casey, 1898

**Family BIPHYLLIDAE LeConte, 1861**

(False skin beetles)

*Diplocoelus brunneus* LeConte, 1863

**Family EROTYLIDAE Latreille, 1802**

(Pleasing fungus beetles)

**Subfamily LANGURIINAE Hope, 1840**

**Tribe Languriini Hope, 1840**

*Acropteroxys gracilis* (Newman, 1838)

**Subfamily EROTYLINAE Latreille, 1802**

**Tribe Dacnini Gistel, 1848**

Dacne (Dacne) quadrimaculata (Say, 1835)

**Tribe Tritomini Curtis, 1834**

*Triplax dissimulator* (Crotch, 1873)

*Triplax flavicollis* Lacordaire, 1842

*Triplax frosti* Casey, 1924

*Triplax macra* LeConte, 1854

*Triplax thoracica* Say, 1825

*Tritoma humeralis* Fabricius, 1801

*Tritoma pulchra* Say, 1826

*Tritoma sanguinipennis* (Say, 1825)

**Family MONOTOMIDAE Laporte, 1840**

(Root-eating beetles)

**Subfamily RHIZOPHAGINAE Redtenbacher, 1845**

Rhizophagus (Anomophagus) brunneus brunneus Horn, 1879

Rhizophagus (Rhizophagus) dimidiatus Mannerheim, 1843

Rhizophagus (Rhizophagus) minutus rotundicollis Bousquet, 1990

Rhizophagus (Rhizophagus) remotus LeConte, 1866

**Subfamily MONOTOMINAE Laporte, 1840**

**Tribe Europini Sen Gupta, 1988**

***Bactridium striolatum* (Reitter, 1873)**

*Pycnotomina cavicollis* (Horn, 1879)

**Tribe Monotomini Laporte, 1840**

***Monotoma americana* Aubé, 1837**

*Monotoma bicolor* A. Villa & G.B. Villa, 1835†

*Monotoma longicollis* (Gyllenhal, 1827)†

*Monotoma picipes* Herbst, 1793†

*Monotoma producta* LeConte, 1855

**Family CRYPTOPHAGIDAE Kirby, 1826**

(Silken fungus beetles)

**Subfamily CRYPTOPHAGINAE Kirby, 1826**

**Tribe Caenoscelini Casey, 1900**

***Caenoscelis basalis* Casey, 1900**

*Caenoscelis subdeplanata* Brisout de Barneville, 1882*

**Tribe Cryptophagini Kirby, 1826**

*Antherophagus convexulus* LeConte, 1863

*Antherophagus ochraceus* Melsheimer, 1844

*Cryptophagus acutangulus* Gyllenhal, 1827*

*Cryptophagus corticinus* C.G. Thomson, 1863*

***Cryptophagus difficilis* Casey, 1900**

*Cryptophagus fallax* Balfour-Browne, 1953†

*Cryptophagus jakowlewi* Reitter, 1888*

*Cryptophagus mainensis* Casey, 1924

*Cryptophagus pilosus* Gyllenhal, 1827†

*Cryptophagus saginatus* Sturm, 1845†

***Cryptophagus scanicus* (Linnaeus, 1758)**†

***Cryptophagus scutellatus* Newman, 1834**†

*Cryptophagus setulosus* Sturm, 1845*

*Cryptophagus subfumatus* Kraatz, 1856†

*Henoticus serratus* (Gyllenhal, 1808)‡

*Henotiderus centromaculatus* Reitter, 1877*

*Myrmedophila americana* (LeConte, 1879)

*Pteryngium crenatum* (Gyllenhal, 1808)†

*Telmatophilus americanus* LeConte, 1863

*Telmatophilus typhae* (Fallén, 1802)†

**Subfamily ATOMARIINAE LeConte, 1861**

**Tribe Atomariini LeConte, 1861**

Atomaria (Anchicera) apicalis Erichson, 1846†

Atomaria (Anchicera) distincta Casey, 1900

Atomaria (Anchicera) ephippiata C.C.A. Zimmermann, 1869

Atomaria (Anchicera) fuscata Schönherr, 1808†

**Atomaria (Anchicera) lederi Johnson, 1970**‡

Atomaria (Anchicera) lewisi Reitter, 1877†

Atomaria (Anchicera) pusilla (Paykull, 1798)†

Atomaria (Anchicera) testacea Stephens, 1830†

Atomaria (Atomaria) nigrirostris Stephens, 1830*

**Family SILVANIDAE Kirby, 1837**

(Silvanid flat bark beetles)

**Subfamily BRONTINAE Blanchard, 1845**

**Tribe Brontini Blanchard, 1845**

*Dendrophagus cygnaei* Mannerheim, 1846

**Tribe Telephanini LeConte, 1861**

***Telephanus atricapillus* Erichson, 1846**

*Telephanus velox* (Haldeman, 1846)

**Subfamily SILVANINAE Kirby, 1837**

***Ahasverus advena* (Waltl, 1834)**†

*Ahasverus longulus* (Blatchley, 1910)

*Nausibius clavicornis* (Kugelann, 1794)†

*Oryzaephilus mercator* (Fauvel, 1889)†

*Oryzaephilus surinamensis* (Linnaeus, 1758)†

*Silvanus bidentatus* (Fabricius, 1792)†

*Silvanus muticus* Sharp, 1899

**Family CUCUJIDAE Latreille, 1802**

(Flat bark beetles)

*Cucujus clavipes clavipes* Fabricius, 1777

*Pediacus fuscus* Erichson, 1845*

**Family PHALACRIDAE Leach, 1815**

(Shining flower beetles)

**Subfamily PHALACRINAE Leach, 1815**

*Acylomus pugetanus* Casey, 1916

*Olibrus semistriatus* LeConte, 1856

*Phalacrus politus* Melsheimer, 1844

*Stilbus apicalis* (Melsheimer, 1844)

**Family LAEMOPHLOEIDAE Ganglbauer, 1899**

(Lined flat bark beetles)

*Charaphloeus convexulus* (LeConte, 1879)

*Cryptolestes pusillus* (Schönherr, 1817)†

***Cryptolestes turcicus* (Grouvelle, 1876)**†

*Laemophloeus biguttatus* (Say, 1825)

*Laemophloeus fasciatus* Melsheimer, 1844

*Leptophloeus angustulus* (LeConte, 1866)

*Placonotus zimmermanni* (LeConte, 1854)

**Family KATERETIDAE Kirby, 1837**

(Short-winged flower beetles)

*Brachypterolus pulicarius* (Linnaeus, 1758)†

*Brachypterus urticae* (Fabricius, 1792)†

*Heterhelus abdominalis* (Erichson, 1843)

*Heterhelus sericans sericans* (LeConte, 1859)

Kateretes (Kateretes) pusillus (Thunberg, 1794)*

**Family NITIDULIDAE Latreille, 1802**

(Sap beetles)

**Subfamily EPURAEINAE Kirejtshuk, 1986**

**Tribe Epuraeini Kirejtshuk, 1986**

Epuraea (Epuraea) aestiva (Linnaeus, 1758)†

Epuraea (Epuraea) erichsoni Reitter, 1873

Epuraea (Epuraea) flavomaculata Mäklin, 1853

Epuraea (Epuraea) helvola Erichson, 1843

Epuraea (Epuraea) linearis Mäklin, 1853*

Epuraea (Epuraea) pallescens labilis Erichson, 1843

Epuraea (Epuraea) planulata Erichson, 1843

Epuraea (Epuraea) rufomarginata (Stephens, 1830)*

Epuraea (Epuraea) terminalis (Mannerheim, 1843)*

Epuraea (Epuraea) truncatella (Mannerheim, 1846)

Epuraea (Epuraeanella) nearctica Kirejtshuk & Kvamme, 2001

Epuraea (Epuraeanella) obtusicollis Reitter, 1873

Epuraea (Epuraeanella) rufa (Say, 1825)

(incertae sedis)

*Epuraea avara* (Randall, 1838)

*Epuraea hornii* Crotch, 1874

*Epuraea obliqua* Hatch, 1962

*Epuraea peltoides* Horn, 1879

***Epuraea populi* Dodge, 1939**

*Epuraea rufida* (Melsheimer, 1844)

*Epuraea umbrosa* Horn, 1879

**Subfamily CARPOPHILINAE Erichson, 1842**

Carpophilus (Carpophilus) hemipterus (Linnaeus, 1758)†

Carpophilus (Carpophilus) sayi Parsons, 1845

Carpophilus (Ecnomorphus) brachypterus (Say, 1825)

Carpophilus (Myothorax) dimidiatus (Fabricius, 1792)†

Carpophilus (Semocarpolus) marginellus Motschulsky, 1858†

**Subfamily MELIGETHINAE C.G. Thomson, 1859**

*Brassicogethes simplipes* (Easton, 1947)

*Brassicogethes viridescens* (Fabricius, 1787)†

*Fabogethes nigrescens* (Stephens, 1830)*

**Subfamily NITIDULINAE Latreille, 1802**

**Tribe Cychramini Gistel, 1848**

*Cychramus adustus* Erichson, 1843

**Tribe Cyllodini Everts, 1898**

*Cyllodes biplagiatus* LeConte, 1866

**Tribe Nitidulini Latreille, 1802**

*Lobiopa undulata* (Say, 1825)

*Nitidula bipunctata* (Linnaeus, 1758)*

*Nitidula rufipes* (Linnaeus, 1767)†

*Omosita colon* (Linnaeus, 1758)†

*Omosita discoidea* (Fabricius, 1775)‡

*Omosita nearctica* Kirejtshuk, 1987

*Phenolia grossa* (Fabricius, 1801)

***Stelidota coenosa* Erichson, 1843**

*Stelidota octomaculata* (Say, 1825)

*Thalycra concolor* (LeConte, 1850)

**Subfamily CILLAEINAE Kirejtshuk & Audisio, 1986**

*Colopterus truncatus* (Randall, 1838)

*Conotelus obscurus* Erichson, 1843

**Subfamily CRYPTARCHINAE C.G. Thomson, 1859**

**Tribe Cryptarchini C.G. Thomson, 1859**

Cryptarcha (Cryptarcha) ampla Erichson, 1843

Cryptarcha (Cryptarcha) strigatula Parsons, 1938

Cryptarcha (Lepiarcha) concinna (Melsheimer, 1853)

Glischrochilus (Glischrochilus) confluentus (Say, 1823)

Glischrochilus (Glischrochilus) moratus W.J. Brown, 1932

Glischrochilus (Glischrochilus) vittatus (Say, 1835)

Glischrochilus (Librodor) fasciatus (Olivier, 1790)

Glischrochilus (Librodor) quadrisignatus (Say, 1835)

Glischrochilus (Librodor) sanguinolentus sanguinolentus (Olivier, 1790)

Glischrochilus (Librodor) siepmanni W.J. Brown, 1932

**Family CERYLONIDAE Billberg, 1820**

(Cerylonid beetles)

**Subfamily CERYLONINAE Billberg, 1820**

*Cerylon castaneum* Say, 1827

*Cerylon unicolor* (Ziegler, 1845)

*Philothermus glabriculus* LeConte, 1863

**Family ENDOMYCHIDAE Leach, 1815**

(Handsome fungus beetles)

**Subfamily ANAMORPHINAE Strohecker, 1953**

***Symbiotes duryi* Blatchley, 1910**

***Symbiotes gibberosus* (Lucas, 1849)**†

**Subfamily LEIESTINAE C.G. Thomson, 1863**

*Phymaphora pulchella* Newman, 1838

**Subfamily DANASCELINAE Tomaszewska, 2000**

*Hadromychus chandleri* Bousquet & Leschen, 2002

**Subfamily ENDOMYCHINAE Leach, 1815**

*Endomychus biguttatus* Say, 1824

**Subfamily STENOTARSINAE Chapuis, 1876**

*Danae testacea* (Ziegler, 1845)

**Subfamily LYCOPERDININAE Bromhead, 1838**

*Lycoperdina ferruginea* J.E. LeConte, 1824

*Mycetina perpulchra* (Newman, 1838)

**Family COCCINELLIDAE Latreille, 1807**

(Ladybird beetles)

**Subfamily MICROWEISEINAE Leng, 920**

**Tribe Microweiseini Leng, 1920**

*Coccidophilus marginatus* (LeConte, 1878)

*Microweisea misella* (LeConte, 1878)

**Subfamily COCCINELLINAE Latreille, 1807**

**Tribe Brachiacanthini Mulsant, 1850**

*Brachiacantha decempustulata* (Melsheimer, 1847)

*Brachiacantha ursina* (Fabricius, 1787)

**Tribe Chilocorini Mulsant, 1846**

*Chilocorus stigma* (Say, 1835)

**Tribe Coccidulini Mulsant, 1846**

*Coccidula lepida* LeConte, 1852

**Tribe Coccinellini Latreille, 1807**

*Adalia bipunctata* (Linnaeus, 1758)‡

*Anatis labiculata* (Say, 1824)

*Anatis mali* (Say, 1824)

*Anisosticta bitriangularis* (Say, 1824)

*Calvia quatuordecimguttata* (Linnaeus, 1758)*

*Coccinella hieroglyphica kirbyi* Crotch, 1874

*Coccinella monticola* Mulsant, 1850

*Coccinella septempunctata* Linnaeus, 1758†

*Coccinella transversoguttata richardsoni* W. Brown, 1962

*Coccinella trifasciata perplexa* Mulsant, 1850

*Coccinella undecimpunctata undecimpunctata* Linnaeus, 1758†

*Harmonia axyridis* (Pallas, 1773)†

*Hippodamia convergens* Guérin-Méneville, 1842

*Hippodamia parenthesis* (Say, 1824)

*Hippodamia quinquesignata quinquesignata* (Kirby, 1837)

*Hippodamia tredecimpunctata tibialis* (Say, 1824)

*Hippodamia variegata* (Goeze, 1777)†

*Macronaemia episcopalis* (Kirby, 1837)

*Mulsantina hudsonica* (Casey, 1899)

*Mulsantina picta* (Randall, 1838)

*Myzia pullata* (Say, 1826)

*Naemia seriata seriata* (Melsheimer, 1847)

*Propylaea quatuordecimpunctata* (Linnaeus, 1758)†

*Psyllobora vigintimaculata* (Say, 1824)

**Tribe Diomini Gordon, 1999**

***Diomus terminatus* (Say, 1835)**

**Tribe Epilachnini Mulsant, 1846**

*Epilachna variventris* Mulsant, 1850

**Tribe Hyperaspidini Mulsant, 1846**

*Hyperaspis bigeminata* (Randall, 1838)

*Hyperaspis binotata* (Say, 1826)

*Hyperaspis consimilis* LeConte, 1852

*Hyperaspis disconotata* Mulsant, 1850

*Hyperaspis octavia* Casey, 1908

*Hyperaspis undulata* (Say, 1824)

**Tribe Scymnini Mulsant, 1846**

***Didion nanum* (LeConte, 1852)**

*Didion punctatum* (Melsheimer, 1847)

Nephus (Nephus) ornatus ornatus (LeConte, 1850)

Scymnus (Pullus) brullei Mulsant, 1850

Scymnus (Pullus) iowensis Casey, 1899

Scymnus (Pullus) lacustris LeConte, 1850

**Tribe Stethorini Dobzhansky, 1924**

*Stethorus punctum punctum* (LeConte, 1852)

**Family CORYLOPHIDAE LeConte, 1852**

(Minute fungus beetles)

**Subfamily CORYLOPHINAE LeConte, 1852**

**Tribe Orthoperini Jacquelin du Val, 1857**

***Orthoperus scutellaris* LeConte, 1878**

*Orthoperus suturalis* LeConte, 1878

**Tribe Parmulini Poey, 1854**

*Clypastraea fasciata* (Say, 1827)

***Clypastraea fusca* (Harold, 1875)**

*Clypastraea lugubris* (LeConte, 1852)

***Clypastraea lunata* (LeConte, 1852)**

**Tribe Peltinodini Paulian, 1950**

***Holopsis marginicollis* (LeConte, 1852)**

**Tribe Rypobiini Paulian, 1950**

*Rypobius marinus* LeConte, 1852

**Tribe Sericoderini Matthews, 1888**

***Sericoderus lateralis* (Gyllenhal, 1827)**†

**Family LATRIDIIDAE Erichson, 1842**

(Minute brown scavenger beetles)

**Subfamily LATRIDIINAE Erichson, 1842**

Cartodere (Aridius) nodifer (Westwood, 1839)†

Cartodere (Cartodere) constricta (Gyllenhal, 1827)†

*Dienerella argus* (Reitter, 1884)†

*Dienerella filiformis* (Gyllenhal, 1827)†

*Dienerella ruficollis* (Marsham, 1802)†

*Enicmus aterrimus* Motschulsky, 1866

*Enicmus fictus* Fall, 1899

*Enicmus histrio* Joy & Tomlin, 1910†

*Enicmus tenuicornis* LeConte, 1878

*Latridius consimilis* Mannerheim, 1844*

***Latridius hirtus* Gyllenhal, 1827**†

*Latridius minutus* (Linnaeus, 1767)†

*Stephostethus breviclavis* (Fall, 1899)

*Stephostethus liratus* (LeConte, 1863)

*Thes bergrothi* (Reitter, 1880)†

**Subfamily CORTICARIINAE Curtis, 1829**

*Corticaria elongata* (Gyllenhal, 1827)†

*Corticaria ferruginea* Marsham, 1802*

*Corticaria impressa* (Olivier, 1790)†

*Corticaria rubripes* Mannerheim, 1844*

*Corticaria saginata* Mannerheim, 1844†

*Corticarina cavicollis* (Mannerheim, 1844)

*Corticarina longipennis* (LeConte, 1855)

*Corticarina minuta* (Fabricius, 1792)*

*Cortinicara gibbosa* (Herbst, 1793)†

Melanophthalma (Cortilena) picta (LeConte, 1855

Melanophthalma (Melanophthalma) helvola Motschulsky, 1866

Melanophthalma (Melanophthalma) inermis Motschulsky, 1866

**Superfamily TENEBRIONOIDEA Latreille, 1802**

**Family MYCETOPHAGIDAE Leach, 1815**

(Hairy fungus beetles)

**Subfamily MYCETOPHAGINAE Leach, 1815**

**Tribe Mycetophagini Leach, 1815**

Litargus (Paralitargus) didesmus (Say, 1826)

Litargus (Tilargus) tetraspilotus LeConte, 1856

Mycetophagus (Ilendus) pluripunctatus LeConte, 1856

Mycetophagus (Mycetophagus) flexuosus Say, 1826

Mycetophagus (Mycetophagus) punctatus Say, 1826

Mycetophagus (Mycetophagus) serrulatus (Casey, 1900)

Mycetophagus (Parilendus) quadriguttatus P.W.J. Müller, 1821‡

**Tribe Typhaeini C.G. Thomson, 1863**

*Typhaea stercorea* (Linnaeus, 1758)†

**Family CIIDAE Leach, 1819**

(Minute tree-fungus beetles)

**Subfamily CIINAE Leach, 1819**

**Tribe Ciini Leach, 1819**

***Ceracis singularis* (Dury, 1917)**

***Ceracis thoracicornis* (Ziegler, 1845)**

*Cis americanus* Mannerheim, 1852

***Cis angustus* Hatch, 1962**

*Cis creberrimus* Mellié, 1849

***Cis fuscipes* Mellié, 1849**

***Cis horridulus* Casey, 1898**

*Cis levettei* (Casey, 1898)

*Cis striatulus* Mellié, 1849*

*Cis striolatus* Casey, 1898

*Cis submicans* Abeille de Perrin, 1874*

*Cis pistorius* Casey, 1898

*Cis laricinus* (Mellié, 1849)*

*Dolichocis indistinctus* Hatch, 1962

*Dolichocis manitoba* Dury, 1919

*Hadreule elongatula* (Gyllenhal, 1827)†

***Malacocis brevicollis* (Casey, 1898)**

***Orthocis punctatus* (Mellié, 1849)**

***Plesiocis cribrum* Casey, 1898**

**Tribe Orophiini C.G. Thomson, 1863**

*Octotemnus glabriculus* (Gyllenhal, 1827)*

*Octotemnus laevis* Casey, 1898

**Family TETRATOMIDAE Billberg, 1820**

(Tetratomid beetles)

**Subfamily TETRATOMINAE Billberg, 1820**

**Tetratoma (Abstrulia) canadensis Nikitsky & Chantal, 2004**

Tetratoma (Abstrulia) tesselata (Melsheimer, 1844)

**Subfamily PISENINAE Miyatake, 1960**

*Pisenus humeralis* (Kirby, 1837)

**Subfamily PENTHINAE Lacordaire, 1859**

*Penthe obliquata* (Fabricius, 1801)

*Penthe pimelia* (Fabricius, 1801)

**Subfamily HALLOMENINAE Gistel, 1848**

**Hallomenus (Hallomenus) binotatus (Quensel, 1790)**†

**Hallomenus (Hallomenus) debilis LeConte, 1866**

**Hallomenus (Hallomenus) scapularis Melsheimer, 1846**

Hallomenus (Xeuxes) serricornis LeConte, 1877

**Subfamily EUSTROPHINAE Gistel, 1848**

**Tribe Eustrophini Gistel, 1848**

*Eustrophus tomentosus* Say, 1826

*Synstrophus repandus* (Horn, 1888)

**Tribe Holostrophini Nikitsky, 1998**

Pseudoholostrophus (Holostrophinus) discolor (Horn, 1888)

**Family MELANDRYIDAE Leach, 1815**

(False darkling beetles)

**Subfamily MELANDRYINAE Leach, 1815**

**Tribe Hypulini Gistel, 1848**

*Hypulus simulator* Newman, 1838

***Microtonus sericans* LeConte, 1862**

*Symphora flavicollis* (Haldeman, 1848)

*Symphora rugosa* (Haldeman, 1848)

*Zilora hispida* LeConte, 1866

*Zilora nuda* Provancher, 1877

**Tribe Melandryini Leach, 1815**

***Emmesa blackmani* (Hatch, 1927)**

*Emmesa connectens* Newman, 1838

*Emmesa labiata* (Say, 1824)

*Melandrya striata* Say, 1824

*Phryganophilus collaris* LeConte, 1859

*Prothalpia undata* LeConte, 1862

**Tribe Orchesiini Mulsant, 1856**

*Microscapha clavicornis* LeConte, 1866

*Orchesia castanea* (Melsheimer, 1846)

*Orchesia cultriformis* Laliberté, 1967

*Orchesia ovata* Laliberté, 1967

**Tribe Serropalpini Latreille, 1829**

*Dircaea liturata* (LeConte, 1866)

*Dolotarsus lividus* (C.R. Sahlberg, 1833)*

*Enchodes sericea* (Haldeman, 1848)

***Phloiotrya concolor* (LeConte, 1866)**

*Phloiotrya fusca* (LeConte, 1878)

*Scotochroa atra* LeConte, 1874

*Scotochroa buprestoides* (Kirby, 1837)

*Scotochroides antennatus* Mank, 1839

*Serropalpus coxalis* Mank, 1839

*Serropalpus substriatus* Haldeman, 1848

*Spilotus quadripustulatus* (Melsheimer, 1846)

*Xylita laevigata* (Hellenius, 1786)*

**Family MORDELLIDAE Latreille, 1802**

(Tumbling flower beetles)

**Subfamily MORDELLINAE Latreille, 1802**

**Tribe Mordellini Latreille, 1802**

*Mordella atrata atrata* Melsheimer, 1846

*Mordella marginata marginata* Melsheimer, 1846

*Mordella melaena* Germar, 1824

*Mordellaria borealis* (LeConte, 1862)

*Mordellaria serval* (Say, 1835)

*Mordellaria undulata* (Melsheimer, 1846)

*Tomoxia inclusa* LeConte, 1862

*Tomoxia lineela* LeConte, 1862

*Yakuhananomia bidentata* (Say, 1824)

**Tribe Mordellistenini Ermisch, 1941**

*Falsomordellistena discolor* (Melsheimer, 1846)

*Falsomordellistena pubescens* (Fabricius, 1798)

*Gilpostenoda ambusta* (LeConte, 1862)

*Mordellina ancilla* (LeConte, 1862)

*Mordellina infima* (LeConte, 1862)

*Mordellina pustulata* (Melsheimer, 1846)

*Mordellistena aspersa* (Melsheimer, 1846)

*Mordellistena cervicalis* LeConte, 1862

*Mordellistena errans* Fall, 1907

*Mordellistena frosti* Liljeblad, 1918

***Mordellistena fulvicollis* (Melsheimer, 1846)**

*Mordellistena fuscipennis* (Melsheimer, 1846)

*Mordellistena indistincta* J.B. Smith, 1882

*Mordellistena limbalis* (Melsheimer, 1846)

*Mordellistena marginalis* (Say, 1824)

*Mordellistena ornata* (Melsheimer, 1846)

*Mordellistena picilabris* Helmuth, 1864

*Mordellistena rubrilabris* Helmuth, 1864

*Mordellistena syntaenia* Liljeblad, 1921

*Mordellistena tosta* LeConte, 1862

*Mordellistena trifasciata* (Say, 1826)

*Mordellochroa scapularis* (Say, 1824)

***Pseudotolida arida* (LeConte, 1862)**

**Family RIPIPHORIDAE Gemminger, 1870**

(Wedge-shaped beetles)

**Subfamily PELECOTOMINAE Seidlitz, 1875**

*Pelecotoma flavipes* Melsheimer, 1846

**Subfamily RIPIPHORINAE Gemminger, 1870**

**Tribe Ripiphorini Gemminger, 1870**

*Ripiphorus fasciatus* (Say, 1824)

**Family ZOPHERIDAE Solier, 1834**

(Zopherid beetles)

**Subfamily COLYDIINAE Billberg, 1820**

**Tribe Synchitini Erichson, 1845**

*Bitoma crenata* (Fabricius, 1775)†

*Lasconotus borealis* Horn, 1878

*Synchita fuliginosa* Melsheimer, 1844

**Subfamily ZOPHERINAE Solier, 1834**

**Tribe Phellopsini Ślipiński and Lawrence, 1999**

*Phellopsis obcordata* (Kirby, 1837)

**Family TENEBRIONIDAE Latreille, 1802**

(Darkling beetles)

**Subfamily LAGRIINAE Latreille, 1825**

**Tribe Goniaderini Lacordaire, 1859**

*Paratenetus exutus* Bousquet & Bouchard, 2014

*Paratenetus punctatus* Spinola, 1844

**Tribe Lagriini Latreille, 1825**

Subtribe Lagriina Latreille, 1825

*Arthromacra aenea aenea* (Say, 1824)

**Subfamily TENEBRIONINAE Latreille, 1802**

**Tribe Alphitobiini Reitter, 1917**

*Alphitobius diaperinus* (Panzer, 1796)†

*Alphitobius laevigatus* (Fabricius, 1781)†

**Tribe Bolitophagini Kirby, 1837**

*Bolitophagus corticola* Say, 1826

*Bolitotherus cornutus* (Fabricius, 1801)

*Eleates depressus* (Randall, 1838)

**Tribe Helopini Latreille, 1802**

*Helops blandi* Bousquet & Bouchard, 2012

**Tribe Opatrini Brullé, 1832**

*Blapstinus metallicus* (Fabricius, 1801)

**Tribe Tenebrionini Latreille, 1802**

*Neatus tenebrioides* (Palisot de Beauvois, 1811)

*Tenebrio molitor* Linnaeus, 1758†

**Tribe Triboliini Gistel, 1848**

*Latheticus oryzae* Waterhouse, 1880†

Tribolium (Tribolium) castaneum (Herbst, 1797)‡

Tribolium (Tribolium) confusum Jacquelin du Val, 1861‡

Tribolium (Tribolium) destructor Uyttenboogaart, 1933†

Tribolium (Tribolium) madens (Charpentier, 1825)†

**Subfamily ALLECULINAE Laporte, 1840**

**Tribe Alleculini Laporte, 1840**

Subtribe Alleculina Laporte, 1840

*Hymenorus molestus* Fall, 1931

*Hymenorus niger* (Melsheimer, 1846)

*Hymenorus obesus* Casey, 1891

Subtribe Gonoderina Seidlitz, 1896

*Androchirus erythropus* (Kirby, 1837)

*Capnochroa fuliginosa* (Melsheimer, 1846)

*Isomira quadristriata* (Couper, 1865)

*Isomira sericea* (Say, 1823)

*Pseudocistela brevis* (Say, 1824)

Subtribe Mycetocharina Gistel, 1848

*Mycetochara analis* (LeConte, 1878)

*Mycetochara bicolor* (Couper, 1865)

*Mycetochara binotata* (Say, 1824)

*Mycetochara foveata* (LeConte, 1866)

*Mycetochara fraterna* (Say, 1824)

**Subfamily DIAPERINAE Latreille, 1802**

**Tribe Diaperini Latreille, 1802**

Subtribe Adelinina LeConte, 1862

***Cynaeus angustus* (LeConte, 1851)**

***Gnatocerus cornutus* (Fabricius, 1798)**†

Subtribe Diaperina Latreille, 1802

*Diaperis maculata* Olivier, 1791

*Neomida bicornis* (Fabricius, 1777)

*Platydema americanum* Laporte & Brullé, 1831

*Platydema teleops* Triplehorn, 1965

**Tribe Hypophlaeini Billberg, 1820**

*Corticeus praetermissus* (Fall, 1926)

*Corticeus tenuis* (LeConte, 1878)

**Tribe Scaphidemini Reitter, 1922**

*Scaphidema aeneolum* (LeConte, 1850)

**Subfamily STENOCHIINAE Kirby, 1837**

**Tribe Cnodalonini Oken, 1843**

*Alobates pensylvanicus* (DeGeer, 1775)

*Iphthiminus opacus* (LeConte, 1866)

*Upis ceramboides* (Linnaeus, 1758)*

*Xylopinus aenescens* LeConte, 1866

*Xylopinus saperdoides* (Olivier, 1795)

**Family SYNCHROIDAE Lacordaire, 1859**

(Synchroid beetles)

*Synchroa punctata* Newman, 1838

**Family STENOTRACHELIDAE C.G. Thomson, 1859**

(False long-horned beetles)

**Subfamily CEPHALOINAE LeConte, 1862**

*Cephaloon lepturoides* Newman, 1838

*Cephaloon ungulare* LeConte, 1874

**Subfamily NEMATOPLINAE LeConte, 1862**

*Nematoplus collaris* LeConte, 1855

**Family OEDEMERIDAE Latreille, 1810**

(False blister beetles)

**Subfamily CALOPODINAE Costa, 1852**

*Calopus angustus* LeConte, 1851

**Subfamily OEDEMERINAE Latreille, 1810**

**Tribe Asclerini Gistel, 1848**

*Asclera puncticollis* (Say, 1824)

*Asclera ruficollis* (Say, 1824)

**Tribe Ditylini Mulsant, 1858**

*Ditylus caeruleus* (Randall, 1838)

**Tribe Nacerdini Mulsant, 1858**

*Nacerdes melanura* (Linnaeus, 1758)†

**Family MELOIDAE Gyllenhal, 1810**

(Blister beetles)

**Subfamily MELOINAE Gyllenhal, 1810**

**Tribe Epicautini Parker & Böving, 1924**

*Epicauta cinerea* (Forster, 1771)

Epicauta (Epicauta) funebris Horn, 1873

Epicauta (Epicauta) pensylvanica (DeGeer, 1775)

Epicauta (Macrobasis) murina (LeConte, 1853)

**Tribe Lyttini Solier, 1851**

Lytta (Pomphopoea) sayi LeConte, 1853

**Tribe Meloini Gyllenhal, 1810**

Meloe (Meloe) angusticollis Say, 1824

Meloe (Meloe) impressus Kirby, 1837

**Family MYCTERIDAE Oken, 1853**

(Mycterid beetles)

**Subfamily EURYPINAE J. Thomson, 1860**

Lacconotus (Lacconotus) punctatus LeConte, 1862

**Family BORIDAE C.G. Thomson, 1859**

(Conifer bark beetles)

**Subfamily BORINAE C.G. Thomson, 1859**

*Boros unicolor* Say, 1827

*Lecontia discicollis* (LeConte, 1850)

**Family PYTHIDAE Solier, 1834**

(Pythid beetles)

*Priognathus monilicornis* (Randall, 1838)

*Pytho americanus* Kirby, 1837

*Pytho niger* Kirby, 1837

*Pytho seidlitzi* Blair, 1925

*Pytho strictus* LeConte, 1866

**Family PYROCHROIDAE Latreille, 1806**

(Fire-colored beetles)

**Subfamily PEDILINAE Lacordaire, 1859**

*Pedilus canaliculatus* (LeConte, 1866)

*Pedilus elegans* (Hentz, 1830)

*Pedilus lugubris* (Say, 1826)

**Subfamily PYROCHROINAE Latreille, 1806**

*Dendroides canadensis* Latreille, 1810

*Dendroides concolor* (Newman, 1838)

***Dendroides testaceus* LeConte, 1855**

*Neopyrochroa femoralis* (LeConte, 1855)

*Schizotus cervicalis* Newman, 1838

**Family SALPINGIDAE Leach, 1815**

(Narrow-waisted bark beetles)

**Subfamily SALPINGINAE Leach, 1815**

*Rhinosimus viridiaeneus* Randall, 1838

*Sphaeriestes virescens* (LeConte, 1850)

**Family ANTHICIDAE Latreille, 1819**

(Antlike flower beetles)

**Subfamily EURYGENIINAE LeConte, 1862**

**Tribe Eurygeniini LeConte, 1862**

*Stereopalpus rufipes* Casey, 1895

**Subfamily ANTHICINAE Latreille, 1819**

**Tribe Anthicini Latreille, 1819**

*Amblyderus pallens* (LeConte, 1850)

*Anthicus cervinus* LaFerté-Sénectère, 1847

*Anthicus coracinus* LeConte, 1852

*Anthicus flavicans* LeConte, 1852

*Anthicus haldemani* LeConte, 1852

*Anthicus heroicus* Casey, 1895

*Anthicus melancholicus* LaFerté-Sénectère, 1847

*Anthicus scabriceps* LeConte, 1850

*Malporus formicarius* (LaFerté-Sénectère, 1847)

*Omonadus floralis* (Linnaeus, 1758)†

*Omonadus formicarius* (Goeze, 1777)†

*Sapintus pubescens* (LaFerté-Sénectère, 1847)

*Sapintus pusillus* (LaFerté-Sénectère, 1847)

**Subfamily NOTOXINAE Stephens, 1829**

*Notoxus anchora* Hentz, 1827

*Notoxus bifasciatus* (LeConte, 1847)

**Family ADERIDAE Csiki, 1909**

(Antlike leaf beetles)

**Tribe Aderini Csiki, 1909**

Subtribe Aderina Csiki, 1909

***Aderus populneus* (Creutzer, 1796)**†

Subtribe Syzetoninina Báguena Corella, 1948

***Vanonus calvescens* Casey, 1895**

*Vanonus huronicus* Casey, 1895

*Vanonus wickhami* Casey, 1895

**Tribe Euglenesini Seidlitz, 1875**

*Zonantes faciatus* (Melsheimer, 1846)

*Zonantes pallidus* Werner, 1990

**Family SCRAPTIIDAE Gistel, 1848**

(Scraptiid beetles)

**Subfamily SCRAPTIINAE Gistel, 1848**

**Tribe Scraptiini Gistel, 1848**

*Canifa pallipes* (Melsheimer, 1846)

*Canifa pusilla* (Haldeman, 1848)

**Subfamily ANASPIDINAE Mulsant, 1856**

**Tribe Anaspidini Mulsant, 1856**

*Anaspis flavipennis* Haldeman, 1848

***Anaspis nigrina* Csiki, 1915**

*Anaspis rufa* Say, 1826

**Family ISCHALIIDAE Blair, 1920**

*Ischalia costata* (LeConte, 1866)

**Superfamily CHRYSOMELOIDEA Latreille, 1802**

**Family CERAMBYCIDAE Latreille, 1802**

(Longhorn beetles)

**Subfamily PARANDRINAE Blanchard, 1845**

**Tribe Parandrini Blanchard, 1845**

*Neandra brunnea* (Fabricius, 1798)

**Subfamily PRIONINAE Latreille, 1802**

**Tribe Meroscelisini J. Thomson, 1860**

*Tragosoma harrisii* LeConte, 1851

**Tribe Prionini Latreille, 1802**

*Orthosoma brunneum* (Forster, 1771)

**Subfamily LEPTURINAE Latreille, 1802**

**Tribe Desmocerini Blanchard, 1845**

*Desmocerus palliatus* (Forster, 1771)

**Tribe Encyclopini LeConte, 1873**

*Encyclops caerulea* (Say, 1826)

**Tribe Lepturini Latreille, 1802**

*Acmaeopsoides rufula* (Haldeman, 1847)

*Analeptura lineola* (Say, 1824)

*Anastrangalia sanguinea* (LeConte, 1859)

Anoplodera (Anoploderomorpha) pubera (Say, 1826)

*Bellamira scalaris* (Say, 1826)

*Brachyleptura champlaini* Casey, 1913

*Brachyleptura circumdata* (Olivier, 1795)

*Brachyleptura rubrica* (Say, 1824)

*Grammoptera haematites* (Newman, 1841)

*Grammoptera subargentata* (Kirby, 1837)

*Idiopidonia pedalis* (LeConte, 1861)

*Judolia montivagans montivagans* (Couper, 1864)

Lepturobosca (Cosmosalia) chrysocoma (Kirby, 1837)

*Lepturopsis biforis* (Newman, 1841)

*Neoalosterna capitata* (Newman, 1841)

*Pedostrangalia deleta* (LeConte, 1850)

*Pedostrangalia plebeja* (Randall, 1838)

*Pedostrangalia subhamata* (Randall, 1838)

*Pygoleptura nigrella nigrella* (Say, 1826)

*Stictoleptura canadensis canadensis* (Olivier, 1795)

*Strangalepta abbreviata* (Germar, 1824)

*Strophiona nitens* (Forster, 1771)

*Trachysida aspera brevifrons* (Howden, 1959)

*Trachysida mutabilis* (Newman, 1841)

*Trigonarthris minnesotana* (Casey, 1913)

*Trigonarthris proxima* (Say, 1824)

*Typocerus acuticauda acuticauda* Casey, 1913

*Typocerus sparsus* LeConte, 1878

*Typocerus velutinus velutinus* (Olivier, 1795)

*Xestoleptura tibialis* (LeConte, 1850)

**Tribe Oxymirini Danilevsky, 1997**

*Anthophylax attenuatus* (Haldeman, 1847)

*Anthophylax cyaneus* (Haldeman, 1847)

*Anthophylax viridis* LeConte, 1850

**Tribe Rhagiini Kirby, 1837**

*Acmaeops discoideus* (Haldeman, 1847)

*Acmaeops proteus proteus* (Kirby, 1837)

*Brachysomida bivittata* (Say, 1824)

*Centrodera decolorata* (Harris, 1841)

*Evodinus monticola monticola* (Randall, 1838)

*Gaurotes cyanipennis* (Say, 1824)

*Gnathacmaeops pratensis* (Laicharting, 1784)*

Pidonia (Pidonia) ruficollis (Say, 1824)

Pidonia (Pidonia) vibex (Newman, 1841)

*Rhagium inquisitor* (Linnaeus, 1758)*

*Stenocorus schaumii* (LeConte, 1850)

*Stenocorus vittiger* (Randall, 1838)

**Tribe Sachalinobiini Danilevsky, 2010**

*Sachalinobia rugipennis* (Newman, 1844)

**Subfamily SPONDYLIDINAE Audinet-Serville, 1832**

**Tribe Asemini J. Thomson, 1860**

*Arhopalus foveicollis* (Haldeman, 1847)

*Arhopalus obsoletus* (Randall, 1838)

*Asemum striatum* (Linnaeus, 1758)*

*Tetropium cinnamopterum* Kirby, 1837

*Tetropium fuscum* (Fabricius, 1787)†

*Tetropium schwarzianum* Casey, 1891

**Tribe Atimiini LeConte, 1873**

*Atimia confusa confusa* (Say, 1826)

**Tribe Spondylidini Audinet-Serville, 1832**

*Neospondylis upiformis* (Mannerheim, 1843)

**Subfamily CERAMBYCINAE Latreille, 1802**

**Tribe Anaglyptini Lacordaire, 1868**

*Cyrtophorus verrucosus* (Olivier, 1795)

*Microclytus compressicollis* (Laporte & Gory, 1835)

**Tribe Callidiini Kirby, 1837**

*Callidium frigidum* Casey, 1912

*Callidium violaceum* (Linnaeus, 1758)†

*Phymatodes aereus* (Newman, 1838)

*Phymatodes amoenus* (Say, 1824)

*Phymatodes dimidiatus* (Kirby, 1837)

*Phymatodes maculicollis* LeConte, 1878

*Phymatodes testaceus* (Linnaeus, 1758)†

*Pronocera collaris collaris* (Kirby, 1837)

*Ropalopus sanguinicollis* (Horn, 1860)

*Semanotus ligneus* (Fabricius, 1787)

*Semanotus terminatus* (Casey, 1912)

**Tribe Clytini Mulsant, 1839**

*Clytus marginicollis* Laporte & Gory, 1835

*Clytus ruricola* (Olivier, 1795)

*Glycobius speciosus* (Say, 1828)

*Megacyllene robiniae* (Forster, 1771)

*Neoclytus acuminatus acuminatus* (Fabricius, 1775)

*Neoclytus leucozonus leucozonus* (Laporte & Gory, 1835)

*Neoclytus mucronatus mucronatus* (Fabricius, 1775)

*Sarosesthes fulminans* (Fabricius, 1775)

*Xylotrechus aceris* Fisher, 1917

*Xylotrechus annosus annosus* (Say, 1826)

*Xylotrechus colonus* (Fabricius, 1775)

*Xylotrechus integer* (Haldeman, 1847)

*Xylotrechus quadrimaculatus* (Haldeman, 1847)

*Xylotrechus sagittatus sagittatus* (Germar, 1821)

*Xylotrechus undulatus* (Say, 1824)

**Tribe Elaphidiini J. Thomson, 1864**

*Anelaphus parallelus* (Newman, 1840)

*Anelaphus villosus* (Fabricius, 1793)

*Psyrassa unicolor* (Randall, 1838)

**Tribe Hesperophanini Mulsant, 1839**

*Tylonotus bimaculatus* Haldeman, 1847

**Tribe Molorchini Gistel, 1848**

*Molorchus bimaculatus bimaculatus* Say, 1824

**Tribe Obriini Mulsant, 1839**

*Obrium rufulum* Gahan, 1908

**Tribe Stenopterini Gistel, 1848**

*Callimoxys sanguinicollis* (Olivier, 1795)

**Tribe Trachyderini Dupont, 1836**

Subtribe Trachyderina Dupont, 1836

*Purpuricenus humeralis* (Fabricius, 1798)

**Subfamily LAMIINAE Latreille, 1825**

**Tribe Acanthocinini Blanchard, 1845**

*Acanthocinus pusillus* (Kirby, 1837)

*Astyleiopus variegatus* (Haldeman, 1847)

*Astylopsis collaris* (Haldeman, 1847)

*Astylopsis macula* (Say, 1826)

*Astylopsis sexguttata* (Say, 1826)

*Graphisurus fasciatus* (DeGeer, 1775)

*Hyperplatys aspersa* (Say, 1824)

*Hyperplatys maculata* Haldeman, 1847

*Lepturges angulatus* (LeConte, 1852)

*Lepturges symmetricus* (Haldeman, 1847)

*Sternidius misellus* (LeConte, 1852)

*Urgleptes querci* (Fitch, 1858)

*Urgleptes signatus* (LeConte, 1852)

**Tribe Acanthoderini J. Thomson, 1860**

*Aegomorphus modestus* (Gyllenhal, 1817)

*Oplosia nubila* (LeConte, 1862)

**Tribe Desmiphorini J. Thomson, 1860**

*Eupogonius subarmatus* (LeConte, 1859)

*Psenocerus supernotatus* (Say, 1823)

**Tribe Monochamini Gistel, 1848**

*Microgoes oculatus* (LeConte, 1862)

*Monochamus carolinensis* (Olivier, 1795)

*Monochamus marmorator* Kirby, 1837

*Monochamus mutator* LeConte, 1850

*Monochamus notatus* (Drury, 1773)

*Monochamus scutellatus* (Say, 1824)

**Tribe Phytoeciini Mulsant, 1839**

*Oberea affinis* Leng & Hamilton, 1896

*Oberea myops* Haldeman, 1847

*Oberea pallida* Casey, 1913

*Oberea praelonga* Casey, 1913

*Oberea schaumii* LeConte, 1852

*Oberea tripunctata* (Swederus, 1787)

**Tribe Pogonocherini Mulsant, 1839**

*Pogonocherus mixtus* Haldeman, 1847

*Pogonocherus parvulus* LeConte, 1852

*Pogonocherus pencillatus* LeConte, 1850

**Tribe Saperdini Mulsant, 1839**

*Saperda calcarata* Say, 1824

*Saperda candida* Fabricius, 1787

*Saperda fayi* Bland, 1863

*Saperda imitans* Felt & Joutel, 1904

*Saperda inornata* Say, 1824

*Saperda lateralis* Fabricius, 1775

*Saperda obliqua* Say, 1826

*Saperda populnea moesta* LeConte, 1850

*Saperda tridentata* Olivier, 1795

*Saperda vestita* Say, 1824

**Tribe Tetropini Portevin, 1927**

*Tetrops praeusta* (Linnaeus, 1758)†

**Family MEGALOPODIDAE Latreille, 1802**

(Megalopodid leaf beetles)

**Subfamily ZEUGOPHORINAE Böving & Craighead, 1931**

**Zeugophora (Zeugophora) abnormis (LeConte, 1850)**

**Zeugophora (Zeugophora) puberula Crotch, 1873**

**Zeugophora (Zeugophora) scutellaris Suffrian, 1840**†

Zeugophora (Zeugophora) varians Crotch, 1873

**Family ORSODACNIDAE C.G. Thomson, 1859**

(Orsodacnid leaf beetles)

**Subfamily ORSODACNINAE C.G. Thomson, 1859**

*Orsodacne atra* (Ahrens, 1810)

**Family CHRYSOMELIDAE Latreille, 1802**

(Leaf beetles)

**Subfamily BRUCHINAE Latreille, 1802**

**Tribe Bruchini Latreille, 1802**

Subtribe Acanthoscelidina Bridwell, 1946

*Acanthoscelides obtectus* (Say, 1831)†

***Bruchidius villosus* (Fabricius, 1775)**†

*Meibomeus musculus* (Say, 1831)

Subtribe Bruchina Latreille, 1802

*Bruchus pisorum* (Linnaeus, 1758)†

Subtribe Megacerina Bridwell, 1946

Megacerus (Megacerus) discoidus (Say, 1824)

**Subfamily DONACIINAE Kirby, 1837**

**Tribe Donaciini Kirby, 1837**

Donacia (Donacia) cincticornis Newman, 1838

Donacia (Donacia) palmata Olivier, 1795

Donacia (Donacia) piscatrix Lacordaire, 1845

Donacia (Donacia) proxima Kirby, 1837

Donacia (Donaciomima) caerulea Olivier, 1795

Donacia (Donaciomima) confluenta Say, 1826

Donacia (Donaciomima) distincta LeConte, 1851

Donacia (Donaciomima) fulgens LeConte, 1851

Donacia (Donaciomima) hirticollis Kirby, 1837

Donacia (Donaciomima) magnifica LeConte, 1851

Donacia (Donaciomima) porosicollis Lacordaire, 1845

Donacia (Donaciomima) rugosa LeConte, 1878

Donacia (Donaciomima) subtilis Kunze, 1818

Donacia (Donaciomima) tuberculifrons C. Schaeffer, 1919

**Tribe Haemoniini Chen, 1941**

*Neohaemonia melsheimeri* (Lacordaire, 1845)

*Neohaemonia nigricornis* (Kirby, 1837)

**Tribe Plateumarini Böving, 1922**

*Plateumaris balli* Askevold, 1991

*Plateumaris flavipes* (Kirby, 1837)

*Plateumaris frosti* (C. Schaeffer, 1925)

*Plateumaris fulvipes* (Lacordaire, 1845)

*Plateumaris germari* (Mannerheim, 1843)

*Plateumaris metallica* (Ahrens, 1810)

*Plateumaris nitida* (Germar, 1811)

*Plateumaris pusilla* (Say, 1826)

*Plateumaris rufa* (Say, 1826)

*Plateumaris shoemakeri* (C. Schaeffer, 1925)

**Subfamily CRIOCERINAE Latreille, 1804**

**Tribe Criocerini Latreille, 1804**

*Crioceris asparagi* (Linnaeus, 1758)†

*Crioceris duodecimpunctata* (Linnaeus, 1758)†

*Liliocerus lilii* (Scopoli, 1763)†

**Tribe Lemini Gyllenhal, 1813**

Lema (Lema) puncticollis (Curtis, 1830)†

Oulema (Oulema) melanopus (Linnaeus, 1758)†

**Subfamily CASSIDINAE Gyllenhal, 1813**

**Tribe Cassidini Gyllenhal, 1813**

Cassida (Cassida) rubiginosa O.F. Müller, 1776†

Charidotella (Chaerocassis) purpurata (Boheman, 1855)

Charidotella (Charidotella) sexpunctata bicolor (Fabricius, 1798)

*Deloyala guttata* (Olivier, 1790)

*Plagiometriona clavata clavata* (Fabricius, 1798)

**Tribe Chalepini Weise, 1910**

Anisostena (Anisostena) nigrita (Olivier, 1808)

*Baliosus nervosus* (Panzer, 1794)

*Odontota dorsalis* (Thunberg, 1805)

*Sumitrosis inaequalis* (Weber, 1801)

*Sumitrosis rosea* (Weber, 1801)

**Tribe Uroplatini Weise, 1910**

*Glyphuroplata pluto* (Newman, 1840)

*Microrhopala excavata excavata* (Olivier, 1808)

*Microrhopala vittata* (Fabricius, 1798)

*Microrhopala xerene* (Newman, 1838)

**Subfamily CHRYSOMELINAE Latreille, 1802**

**Tribe Chrysomelini Latreille, 1802**

Calligrapha (Bidensomela) bidenticola W.J. Brown, 1945

Calligrapha (Bidensomela) californica coreopsivora W.J. Brown, 1945

Calligrapha (Calligrapha) alni C. Schaeffer, 1928

Calligrapha (Calligrapha) alnicola W.J. Brown, 1945

Calligrapha (Calligrapha) confluens C. Schaeffer, 1928

Calligrapha (Calligrapha) ignota W.J. Brown, 1945

Calligrapha (Calligrapha) multipunctata (Say, 1824)

Calligrapha (Calligrapha) philadelphica (Linnaeus, 1758)

Calligrapha (Calligrapha) rowena Knab, 1909

Calligrapha (Calligrapha) suturella C. Schaeffer, 1933

Calligrapha (Calligrapha) tiliae W.J. Brown, 1945

Calligrapha (Calligrapha) vicina C. Schaeffer, 1933

Calligrapha (Calligrapha) virginea W.J. Brown, 1945

Calligrapha (Graphicallo) lunata (Fabricius, 1787)

Chrysolina (Chalcoidea) marginata marginata (Linnaeus, 1758)

Chrysolina (Hypericia) hyperici hyperici (Forster, 1771)†

Chrysolina (Hypericia) quadrigemina (Suffrian, 1851)†

Chrysomela (Chrysomela) crotchi W.J. Brown, 1956

Chrysomela (Macrolina) laurentia W.J. Brown, 1956

Chrysomela (Macrolina) lineatopunctata (Forster, 1771)

Chrysomela (Macrolina) mainensis mainensis Bechyné, 1954

Gastrophysa (Gastrophysa) polygoni (Linnaeus, 1758)†

Gonioctena (Gonioctena) americana (C. Schaeffer, 1924)

*Labidomera clivicollis* (Kirby, 1837)

*Leptinotarsa decemlineata* (Say, 1824)

Phaedon (Phaedon) armoraciae armoraciae (Linnaeus, 1758)†

Phaedon (Phaedon) laevigatus (Duftschmid, 1825)†

Phaedon (Phaedon) oviformis (LeConte, 1861)

Phaedon (Phaedon) viridis Melsheimer, 1847

Phratora (Phratora) americana canadensis W.J. Brown, 1951

Phratora (Phratora) purpurea purpurea W.J. Brown, 1951

Plagiodera (Plagiodera) versicolora (Laicharting, 1781)†

Prasocuris (Hydrothassa) vittata (Olivier, 1807)

**Subfamily GALERUCINAE Latreille, 1802**

**Tribe Alticini Newman, 1834**

Altica (Altica) ambiens alni Harris, 1869

Altica (Altica) browni Mohamedsaid, 1984

Altica (Altica) carinata Germar, 1824

Altica (Altica) chalybaea Illiger, 1807

Altica (Altica) corni Woods, 1918

Altica (Altica) kalmiae (Melsheimer, 1847)

Altica (Altica) knabii (Blatchley, 1910)

Altica (Altica) prasina populi W.J. Brown, 1938

Altica (Altica) rosae Woods, 1918

Altica (Altica) sylvia Malloch, 1919

Altica (Altica) tombacina Mannerhiem, 1853

Altica (Altica) ulmi Woods, 1918

Altica (Altica) woodsi Isely, 1920

*Capraita subvittata* (Horn, 1889)

*Chaetocnema borealis* R. White, 1996

*Chaetocnema concinna* (Marsham, 1802)†

*Chaetocnema confinis* Crotch, 1873

*Chaetocnema minuta* Melsheimer, 1847

*Chaetocnema protensa* LeConte, 1878

*Crepidodera heikertingeri* (Lazorko, 1974)

*Crepidodera luminosa* Parry, 1986

*Crepidodera nana* (Say, 1824)

*Crepidodera populivora* Parry, 1986

*Crepidodera violacea* Melsheimer, 1847

*Dibolia borealis* Chevrolat, 1834

*Dibolia chelones* Parry, 1974

*Dibolia melampyri* Parry, 1974

*Disonycha alternata* (Illiger, 1807)

*Disonycha latifrons* C. Schaeffer, 1919

*Disonycha procera* Casey, 1884

*Disonycha uniguttata* (Say, 1824)

*Disonycha xanthomelas* (Dalman, 1823)

*Distigmoptera borealis* Blake, 1943

*Distigmoptera impennata* Blake, 1943

*Epitrix cucumeris* (Harris, 1851)

*Hippuriphila canadensis* W.J. Brown, 1942

*Kuschelina vians* (Illiger, 1807)

*Longitarus erro* Horn, 1889

***Longitarsus ganglbauri* Heikertinger, 1873**†

*Longitarus jacobaeae* Waterhouse, 1858†

*Longitarus luridis* (Scopoli, 1763)†

***Longitarsus rubiginosus* (Foudras, 1859)**†

*Longitarus testaceus* Melsheimer, 1847

*Mantura chrysanthemi* (Koch, 1803)†

*Neocrepidodera robusta* (LeConte, 1874)

*Phyllotreta aerea* Allard, 1859

*Phyllotreta armoraciae* (Koch, 1803)†

*Phyllotreta cruciferae* (Goeze, 1777)†

*Phyllotreta robusta* LeConte, 1878

*Phyllotreta striolata* (Fabricius, 1803)†

*Phyllotreta zimmermanni* (Crotch, 1873)*

*Psylliodes affinis* (Paykull, 1799)†

*Psylliodes cucullatus* (Illiger, 1807)†

*Psylliodes napi* (Fabricius, 1792)†

***Psylliodes picinus* (Marsham, 1802)**†

*Psylliodes punctulatus* Melsheimer, 1847

*Systena frontalis* (Fabricius, 1801)

*Systena hudsonias* (Forster, 1771)

**Tribe Galerucini Latreille, 1802**

*Erynephala maritima* (LeConte, 1865)

Galerucella (Galerucella) nymphaeae (Linnaeus, 1758)*

*Neogalerucella calmariensis* (Linnaeus, 1767)†

*Neogalerucella pusilla* (Duftschmid, 1825)†

*Ophraella communa* LeSage, 1986

*Ophraella conferta* (LeConte, 1865)

*Ophraella cribrata* (LeConte, 1865)

*Ophraella notata* (Fabricius, 1801)

*Pyrrhalta viburni* (Paykull, 1799)†

*Tricholochmaea alni* (Fall, 1924)

*Tricholochmaea cavicollis* (LeConte, 1865)

*Tricholochmaea decora decora* (Say, 1824)

*Tricholochmaea kalmiae* (Fall, 1924)

*Tricholochmaea perplexa* (Fall, 1924)

*Tricholochmaea ribicola ribicola* (W.J. Brown, 1938)

*Tricholochmaea rufosanguinea* (Say, 1826)

*Tricholochmaea spiraeae* (Fall, 1924)

*Tricholochmaea tuberculata* (Say, 1824)

*Tricholochmaea vaccinii* (Fall, 1924)

*Trirhabda borealis* Blake, 1931

*Trirhabda canadensis* (Kirby, 1837)

*Trirhabda virgata* LeConte, 1865

*Xanthogaleruca luteola* (O.F. Müller, 1766)†

**Tribe Luperini Gistel, 1848**

*Acalymma gouldi* Barber, 1947

*Acalymma vittatum* (Fabricius, 1775)

*Diabrotica barberi* R.F. Smith & Lawrence, 1967

*Diabrotica undecimpunctata howardi* Barber, 1947

*Phyllobrotica decorata* (Say, 1824)

*Phyllobrotica limbata* (Fabricius, 1801)

*Scelolyperus meracus* (Say, 1826)

**Subfamily CRYPTOCEPHALINAE Gyllenhal, 1813**

**Tribe Cryptocephalini Gyllenhal, 1813**

Subtribe Cryptocephalina Gyllenhal, 1813

*Bassareus formosus* (Melsheimer, 1847)

*Bassareus mammifer* (Newman, 1840)

*Cryptocephalus gibbicollis gibbicollis* Haldeman, 1849

*Cryptocephalus notatus* Fabricius, 1787

*Cryptocephalus venustus* Fabricius, 1787

*Diachus auratus* (Fabricius, 1801)

*Diachus catarius* (Suffrian, 1852)

*Triachus vacuus* LeConte, 1880

Subtribe Monachulina Leng, 1920

*Lexiphanes saponatus* (Fabricius, 1801)

Subtribe Pachybrachina Chapuis, 1874

Pachybrachis (Pachybrachis) bivittatus (Say, 1824)

Pachybrachis (Pachybrachis) hepaticus hepaticus (Melsheimer, 1847)

Pachybrachis (Pachybrachis) m-nigrum (Melsheimer, 1847)

Pachybrachis (Pachybrachis) nigricornis (Say, 1824)

Pachybrachis (Pachybrachis) obsoletus Suffrian, 1852

Pachybrachis (Pachybrachis) peccans Suffrian, 1852

**Pachybrachis (Pachybrachis) tridens (Melsheimer, 1847)**

**Tribe Fulcidacini Jakobson, 1924**

*Exema canadensis* Pierce, 1940

***Neochlamisus bebbiane* (W.J. Brown, 1943)**

***Neochlamisus chamaedaphnes* (W.J. Brown, 1943)**

*Neochlamisus comptoniae* (W.J. Brown, 1943)

*Neochlamisus cribripennis* (LeConte, 1878)

*Neochlamisus eubati* (W.J. Brown, 1943)

*Neochlamisus fragariae* (W.J. Brown, 1943)

**Subfamily EUMOLPINAE Hope, 1840**

**Tribe Bromiini Baly, 1865**

*Bromius obscurus* (Linnaeus, 1758)*

*Graphops pubescens* (Melsheimer, 1847)

*Xanthonia decemnotata* (Say, 1824)

***Xanthonia villosula* (Melsheimer, 1847)**

**Tribe Eumolpini Hope, 1840**

*Chrysochus auratus* (Fabricius, 1775)

**Tribe Typophorini Baly, 1865**

*Paria fragariae fragariae* Wilcox, 1954

***Paria pratensis* Balsbaugh, 1970**

*Paria thoracica* (Melsheimer, 1847)

**Subfamily SYNETINAE LeConte & Horn, 1883**

*Syneta extorris borealis* W. J. Brown, 1961

*Syneta ferruginea* (Germar, 1811)

*Syneta pilosa* W.J. Brown, 1940

**Superfamily CURCULIONOIDEA Latreille, 1802**

**Family NEMONYCHIDAE Bedel, 1882**

(Pine flower snout beetles)

**Subfamily CIMBERIDINAE Gozis, 1882**

**Tribe Cimberidini Gozis, 1882**

*Cimberis elongata* (LeConte, 1876)

***Cimberis pallipennis* (Blatchley, 1916)**

*Cimberis pilosa* (LeConte, 1876)

**Family ANTHRIBIDAE Billberg, 1820**

(Fungus weevils)

**Subfamily ANTHRIBINAE Billberg, 1820**

**Tribe Cratoparini LeConte, 1876**

*Euparius marmoreus* (Olivier, 1795)

*Euparius paganus* Gyllenhal, 1833

**Tribe Stenocerini Kolbe, 1895**

*Allandrus bifasciatus* LeConte, 1876

*Allandrus populi* Pierce, 1930

**Tribe Trigonorhinini Valentine, 1999**

*Trigonorhinus sticticus* (Boheman, 1833)

**Tribe Tropiderini Lacordaire, 1865**

*Eurymycter fasciatus* (Olivier, 1795)

*Eurymycter latifascia* (Pierce, 1930)

*Gonotropis dorsalis* (Thunberg, 1796)*

**Tribe Zygaenodini Lacordaire, 1865**

***Eusphyrus walshii* LeConte, 1876**

*Ormiscus saltator* LeConte, 1876

**Subfamily CHORAGINAE Kirby, 1819**

**Tribe Choragini Kirby, 1819**

***Choragus harrisii* LeConte, 1878**

*Choragus sayi* LeConte, 1876

***Charagus zimmermanni* LeConte, 1878**

*Euxenus punctatus* LeConte, 1876

**Family ATTELABIDAE Billberg, 1820**

(Leaf-rolling weevils)

**Subfamily ATTELABINAE Billberg, 1820**

**Tribe Attelabini Billberg, 1820**

Subtribe Himatolabina Legalov, 2003

*Himatolabus pubescens* (Say, 1826)

**Subfamily RHYNCHITINAE Gistel, 1848**

**Tribe Auletini Desbrochers des Loges, 1908**

Subtribe Auletina Desbrochers des Loges, 1908

Auletobius (Mesauletes) cassandrae (LeConte, 1876)

**Tribe Rhynchitini Gistel, 1848**

*Temnocerus cyanellus* (LeConte, 1876)

*Temnocerus perplexus* (Blatchley, 1916)

**Family BRENTIDAE Billberg, 1820**

(Straight-snouted weevils)

**Subfamily Brentinae Billberg, 1820**

**Tribe Brentini Billberg, 1820**

Subtribe Arrhenodina Lacordaire, 1865

*Arrhenodes minutus* (Drury, 1773)

**Subfamily APIONINAE Schönherr, 1823**

**Supertribe APIONITAE Schönherr, 1823**

**Tribe Apionini Schönherr, 1823**

Subtribe Aplemonina Kissinger, 1968

*Perapion curtirostre* (Germar, 1817)†

Subtribe Ixapiina Alonso-Zarazaga, 1990

Neapion (Xixias) frosti (Kissinger, 1968)

Subtribe Oxystomatina Alonso–Zarazaga, 1990

*Loborhynchapion cyanitinctum* (Fall, 1827)

Subtribe Piezotrachelina Voss, 1959

*Fallapion finitimum* (Fall, 1898)

*Fallapion pennsylvanicum* (Boheman, 1839)

Subtribe Synapiina Alonso-Zarazaga, 1990

Ischnoterapion (Chorapion) virens (Herbst, 1797)†

Subtribe Trichapiina Alonso-Zarazaga, 1990

*Betulapion simile walshii* (J.B. Smith, 1884)

*Trichapion nigrum* (Herbst, 1797)

*Trichapion porcatum* (Boheman, 1839)

*Trichapion reconditum* (J.B. Smith, 1884)

Apionini Incertae Sedis

*Coelocephalapion emaciipes* (Fall, 1898)

**Tribe Podapiini Wanat, 2001**

*Podapion gallicolla* Riley, 1883

**Subfamily NANOPHYINAE Gistel, 1848**

**Tribe Nanophyini Gistel, 1848**

***Nanophyes marmoratus marmoratus* (Goeze, 1777)**†

**Family DRYOPHTHORIDAE Schönherr, 1825**

**Subfamily DRYOPHTHORINAE Schönherr, 1825**

*Dryophthorus americanus* Bedel, 1885

**Subfamily RHYNCHOPHORINAE Schönherr, 1833**

**Tribe Litosomini Lacordaire, 1865**

*Sitophilus granarius* (Linnaeus, 1758)†

*Sitophilus oryzae* (Linnaeus, 1763)†

**Tribe Sphenophorini Lacordaire, 1865**

*Sphenophorus costipennis* Horn, 1873

*Sphenophorus parvulus* Gyllenhal, 1838

*Sphenophorus pertinax pertinax* (Olivier, 1807)

*Sphenophorus striatipennis* Chittenden, 1905

*Sphenophorus venatus venatus* (Say, 1832)

*Sphenophorus zeae* Walsh, 1867

**Family BRACHYCERIDAE Billberg, 1820**

**Subfamily ERIRHININAE Schönherr, 1825**

**Tribe Erirhinini Schönherr, 1825**

*Grypus equiseti* (Fabricius, 1775)*

*Notaris aethiops* (Fabricius, 1792)*

*Notaris puncticollis* (LeConte, 1876)

***Procas lecontei* Bedel, 1879**

*Tournotaris bimaculata* (Fabricius, 1787)*

*Notiodes ovalis* (LeConte, 1876)

*Onychylis nigrirostris* (Boheman, 1843)

**Tribe Tanysphyrini Gistel, 1848**

*Tanysphyrus lemnae* (Fabricius, 1792)†

**Family CURCULIONIDAE Latreille, 1802**

(Snout beetles)

**Subfamily CURCULIONINAE Latreille, 1802**

**Tribe Acalyptini C.G. Thomson, 1859**

Subtribe Acalyptina C.G. Thomson, 1859

*Acalyptus carpini* (Fabricius, 1792)

**Tribe Anthonomini C.G. Thomson, 1859**

Anthonomus (Anthonomus) corvulus LeConte, 1876

Anthonomus (Anthonomus) haematopus Boheman, 1843

Anthonomus (Anthonomus) interstitialis Dietz, 1891

Anthonomus (Anthonomus) lecontei Burke, 1975

Anthonomus (Anthonomus) molochinus Dietz, 1891

**Anthonomus (Anthonomus) pusillus LeConte, 1876**

Anthonomus (Anthonomus) robustulus LeConte, 1876

Anthonomus (Anthonomus) rutilus (Boheman, 1843)

Anthonomus (Anthonomus) signatus Say, 1832

Anthonomus (Anthonomus) simiolus Blatchley, 1916

Anthonomus (Anthonomus) subfasciatus LeConte, 1876

Anthonomus (Cnemocyllus) elongatus LeConte, 1876

**Anthonomus (Cnemocyllus) pictus Blatchley, 1922**

Anthonomus (Paranthonomus) profundus LeConte, 1876

Anthonomus (Tachypterellus) quadrigibbus Say, 1832

*Pseudanthonomus crataegi* (Walsh, 1867)

*Pseudanthonomus seriesetosus* Dietz, 1891

*Pseudanthonomus validus* Dietz, 1891

**Tribe Curculionini Latreille, 1802**

Subtribe Archariina Pelsue & O’Brien, 2011

***Archarius salicivorus* (Paykull, 1792)**†

Subtribe Curculionina Latreille, 1802

*Curculio nasicus* (Say, 1831)

*Curculio obtusus* (Blanchard, 1884)

*Curculio sulcatulus* (Casey, 1897)

**Tribe Ellescini C.G. Thomson, 1859**

Subtribe Dorytomina Bedel, 1886

*Dorytomus frostii* Blatchley, 1916

***Dorytomus hirtus* LeConte, 1876**

*Dorytomus laticollis* LeConte, 1876

*Dorytomus luridis* (Mannerheim, 1853)

*Dorytomus marmoreus* Casey, 1892

*Dorytomusm parvicollis* Casey, 1892

*Dorytomus vagenotatus* Casey, 1892

Subtribe Ellescina C.G. Thomson, 1859

***Ellescus bipunctatus* (Linnaeus, 1758)**

*Ellescus ephippiatus* (Say, 1832)

*Proctorus armatus* LeConte, 1876

*Proctorus decipiens* (LeConte, 1876)

**Tribe Mecinini Gistel, 1848**

*Cleopomiarus hispidulus* (LeConte, 1876)

***Mecinus janthinus* (Germar, 1821)**†

*Rhinusa antirrhini* (Paykull, 1800)†

*Rhinusa tetra* (Fabricius, 1792)†

**Tribe Otidocephalini Lacordaire, 1863**

***Myrmex chevrolatii* (Horn, 1873)**

**Tribe Piazorhinini Lacordaire, 1863**

*Piazorhinus pictus* LeConte, 1876

*Piazorhinus scutellaris* (Say, 1826)

**Tribe Rhamphini Rafinesque, 1815**

Subtribe Rhamphina Rafinesque, 1815

*Isochnus rufipes* (LeConte, 1876)

*Isochnus sequensi* (Stierlin, 1894)†

*Orchestes mixtus* Blatchley, 1916

*Orchestes pallicornis* Say, 1832

*Orchestes testaceus* (O.F. Müller, 1776)*

*Tachyerges ephippiatus* (Say, 1832)

*Tachyerges niger* (Horn, 1873)

*Tachyerges salicis* (Linnaeus, 1758)*

**Tribe Smicronychini Seidlitz, 1891**

Smicronyx (Pseudosmicronyx) corniculatus (Fåhraeus, 1843)

**Tribe Tychiini Gistel, 1848**

Subtribe Lignyodina Bedel, 1883

*Lignyodes bischoffi* (Blatchley, 1916)

*Lignyodes helvolus* (LeConte, 1876)

*Lignyodes horridulus* (Casey, 1892)

Subtribe Tychiina Gistel, 1848

*Tychius meliloti* Stephens, 1831†

*Tychius picirostris* (Fabricius, 1787)†

*Tychius stephensi* Schönherr, 1836†

**Subfamily BAGOINAE C.G. Thomson, 1859**

*Bagous nebulosus* LeConte, 1876

*Bagous obliquus* LeConte, 1876

*Bagous planatus* LeConte, 1876

**Subfamily BARIDINAE Schönherr, 1836**

**Tribe Apostasimerini Schönherr, 1844**

Subtribe Zygobaridina Pierce, 1907

*Cylindridia prolixa* (LeConte, 1876)

Dirabius (Dirabius) rectirostris (LeConte, 1876)

*Stethobaris commixta* Blatchley, 1916

*Stethobaris ovata* (LeConte, 1868)

**Tribe Baridini Schönherr, 1836**

Subtribe Baridina Schönherr, 1836

*Cosmobaris scolopacea* Germar, 1819†

*Plesiobaris disjuncta* Casey, 1892

**Tribe Madarini Jekel, 1865**

Subtribe Leptoschoinina Lacordaire, 1865

*Odontocorynus salebrosus* (Casey, 1892)

Subtribe Madarina Jekel, 1865

***Madarellus undulatus* (Say, 1824)**

**Subfamily CEUTORHYNCHINAE Gistel, 1848**

**Tribe Ceutorhynchini Gistel, 1848**

*Amalus scortillum* (Herbst, 1795)†

*Ceutorhynchus americanus* Buchanan, 1937

*Ceutorhynchus erysimi* (Fabricius, 1787)†

*Ceutorhynchus hamiltoni* Dietz, 1896

*Ceutorhynchus neglectus* Blatchley, 1916

*Ceutorhynchus obstrictus* (Marsham, 1802)†

*Ceutorhynchus omissus* Fall, 1917

*Ceutorhynchus querceti* (Gyllenhal, 1813)*

*Ceutorhynchus semirufus* LeConte, 1876

*Ceutorhynchus typhae* (Herbst, 1795)†

*Glocianus punctiger* (C.R. Sahlberg, 1835)†

***Microplontus campestris* (Gyllenhal, 1837)**†

*Nedyus apicalis* (Dietz, 1896)

**Tribe Cnemogonini Colonnelli, 1979**

*Acanthoscelidius acephalus* (Say, 1824)

*Auleutes epilobii* (Paykull, 1800)*

*Auleutes tenuipes* (LeConte, 1876)

*Cnemogonus lecontei* Dietz, 1896

*Dietzella zimmermanni* (Gyllenhal, 1837)

*Parauleutes nebulosus* (LeConte, 1876)

*Perigaster cretura* (Herbst, 1797)

*Perigaster liturata* (Dietz, 1896)

**Tribe Mononychini LeConte, 1876**

*Mononychus vulpeculus* (Fabricius, 1801)

**Tribe Phytobiini Gistel, 1848**

*Euhrychiopsis lecontei* (Dietz, 1896)

*Parenthis vestitus* Dietz, 1896

*Pelenomus fuliginosus* (Dietz, 1896)

*Pelenomus squamosus* LeConte, 1876

*Pelenomus sulcicollis* (Fåhraeus, 1843)

***Pelenomus waltoni* (Boheman, 1843)**†

***Rhinoncus bruchoides* (Herbst, 1784)**†

*Rhinoncus leucostigma* (Marsham, 1802)†

*Rhinoncus pericarpius* (Linnaeus, 1758

*Rhinoncus longulus* LeConte, 1876

*Rhinoncus pericarpius* (Linnaeus, 1758)†

*Rhinoncus castor* (Fabricius, 1792)

***Rhinoncus perpendicularis* (Reich, 1797)**†

*Rhinoncus pyrrhopus* Boheman, 1845†

**Tribe Scleropterini Schultze, 1902**

*Acallodes saltoides* Dietz, 1896

**Subfamily CONODERINAE Schönherr, 1833**

**Tribe Lechriopini Lacordaire, 1865**

*Acoptus suturalis* LeConte, 1876

*Lechriops oculatus* (Say, 1824)

*Psomus armatus* Dietz, 1891

**Tribe Zygopini Lacordaire, 1865**

*Cylindrocopturus longulus* (LeConte, 1876)

**Subfamily COSSONINAE Schönherr, 1825**

**Tribe Cossonini Schönherr, 1825**

*Cossonus americanus* Buchanan, 1936

***Cossonus impressifrons* Boheman, 1838**

***Cossonus pacificus* Van Dyke, 1916**

*Cossonus platalea* Say, 1832

**Tribe Onycholipini Wollaston, 1873**

*Stenoscelis brevis* (Boheman, 1845)

**Tribe Rhyncolini Gistel, 1848**

Subtribe Phloeophagina Voss, 1955

*Phloeophagus apionides* Horn, 1873

*Phloeophagus canadensis* Van Dyke, 1927

*Phloeophagus minor* Horn, 1873

Subtribe Rhyncolina Gistel, 1848

*Carphonotus testaceus* Casey, 1892

*Himatium errans* LeConte, 1876

*Rhyncolus brunneus* Mannerheim, 1843

***Rhyncolus knowltoni* (Thatcher, 1940)**

*Rhyncolus macrops* Buchanan, 1946

**Subfamily CRYPTORHYNCHINAE Schönherr, 1825**

**Tribe Cryptorhynchini Schönherr, 1825**

Subtribe Cryptorhynchina Schönherr, 1825

*Cryptorhynchus lapathi* (Linnaeus, 1758)*

***Eubulus bisignatus* (Say, 1832)**

*Eubulus parochus* (Herbst, 1797)

*Tyloderma nigrum* Casey, 1884

**Subfamily CYCLOMINAE Schönherr, 1826**

**Tribe Listroderini LeConte, 1876**

*Listronotus alternatus* (Dietz, 1889)

*Listronotus appendiculatus* (Boheman, 1842)

*Listronotus caudatus* (Say, 1824)

*Listronotus deceptus* (Blatchley, 1916)

*Listronotus delumbis* (Gyllenhal, 1834)

*Listronotus humilis* (Gyllenhal, 1834)

*Listronotus laramiensis* (Angell, 1893)

*Listronotus lutulentus* (Boheman, 1843)

*Listronotus maculicollis* (Kirby, 1837)

*Listronotus oregonensis oregonensis* (LeConte, 1857)

*Listronotus sparsus* (Say, 1832)

*Listronotus squamiger* (Say, 1832)

*Listronotus tuberosus* LeConte, 1876

**Subfamily ENTIMINAE Schönherr, 1823**

**Tribe Cneorhinini Lacordaire, 1863**

*Philopedon plagiatum* (Schaller, 1783)†

**Tribe Geonemini Gistel, 1848**

*Barynotus obscurus* (Fabricius, 1775)†

*Barynotus schoenherri* (Zetterstedt, 1838)†

**Tribe Hormorini Horn, 1876**

*Hormorus undulatus* (Uhler, 1856)

**Tribe Otiorhynchini Schönherr, 1826**

*Otiorhynchus ligneus* (Olivier, 1807)†

*Otiorhynchus ovatus* (Linnaeus, 1758)†

*Otiorhynchus rugifrons* (Gyllenhal, 1813)†

*Otiorhynchus singularis* (Linnaeus, 1767)†

*Otiorhynchus sulcatus* (Fabricius, 1775)†

**Tribe Peritelini Lacordaire, 1863**

*Nemocestes horni* Van Dyke, 1936

**Tribe Phyllobiini Schönherr, 1826**

*Phyllobius intrusus* Kôno, 1948†

*Phyllobius oblongus* (Linnaeus, 1758)†

**Tribe Polydrusini Schönherr, 1823**

*Pachyrhinus elegans* (Couper, 1865)

***Polydrusus cervinus* (Linnaeus, 1758)**†

*Polydrusus formosus* (Mayer, 1779)†

*Polydrusus impressifrons* Gyllenhal, 1834†

**Tribe Sciaphilini Sharp, 1891**

*Barypeithes pellucidus* (Boheman, 1834)†

*Sciaphilus asperatus* (Bonsdorff, 1785)†

**Tribe Sitonini Gistel, 1848**

*Sitona cylindricollis* Fåhraeus, 1840†

*Sitona hispidulus* (Fabricius, 1777)†

*Sitona lepidus* Gyllenhal, 1834†

*Sitona lineellus* (Bonsdorff, 1785)*

**Tribe Trachyphloeini Gistel, 1848**

Subtribe Trachyphloeina Gistel, 1848

*Romualdius bifoveolatus* (Beck, 1817)†

**Tribe Tropiphorini Marseul, 1863**

*Phyxelis rigidus* (Say, 1832)

*Tropiphorus terricola* (Newman, 1838)†

**Subfamily HYPERINAE Marseul, 1863**

**Tribe Hyperini Marseul, 1863**

Brachypera (Antidonus) zoilus (Scopoli, 1763)†

*Hypera castor* (LeConte, 1876)

*Hypera compta* (Say, 1832)

*Hypera meles* (Fabricius, 1792)†

*Hypera nigrirostris* (Fabricius, 1775)†

*Hypera postica* (Gyllenhal, 1813)†

**Subfamily LIXINAE Schönherr, 1823**

**Tribe Cleonini Schönherr, 1823**

*Cleonis pigra* (Scopoli, 1763)†

*Scaphomorphus calandroides* (Randall, 1838)

**Tribe Lixini Schönherr, 1823**

*Lixus rubellus* Randall, 1838

**Subfamily MESOPTILIINAE Lacordaire, 1863**

**Tribe Magdalidini Pascoe, 1870**

*Magdalis alutacea* LeConte, 1878

*Magdalis armicollis* (Say, 1824)

*Magdalis barbita* (Say, 1832)

*Magdalis gentilis* LeConte, 1876

*Magdalis hispoides* LeConte, 1876

*Magdalis inconspicua* Horn, 1873

*Magdalis perforata* Horn, 1873

***Magdalis piceae* Buchanan, 1934**

*Magdalis salicis* Horn, 1873

**Subfamily MOLYTINAE Schönherr, 1823**

**Tribe Conotrachelini Jekel, 1865**

*Conotrachellus anaglypticus* (Say, 1832)

*Conotrachellus juglandis* LeConte, 1876

*Conotrachellus nenuphar* (Herbst, 1797)

*Conotrachellus posticatus* Boheman, 1837

**Tribe Hylobiini Kirby, 1837**

Subtribe Hylobiina Kirby, 1837

*Hylobius congener* Dalla Torre, Schenkling & Marshall, 1932

*Hylobius pales* (Herbst, 1797)

*Hylobius pinicola* (Couper, 1864)

*Hylobius warreni* Wood, 1957

**Tribe Lepyrini Kirby, 1837**

*Lepyrus palustris* (Scopoli, 1763)*

**Tribe Molytini Schönherr, 1823**

Subtribe Plinthina Lacordaire, 1863

*Sthereus ptinoides* (Germar, 1824)*

**Tribe Pissodini Gistel, 1848**

Subtribe Pissodina Gistel, 1848

*Pissodes affinis* Randall, 1838

*Pissodes fiskei* Hopkins, 1911

*Pissodes nemorensis* Germar, 1824

*Pissodes rotundatus* LeConte, 1876

*Pissodes similis* Hopkins, 1911

*Pissodes striatulus* (Fabricius, 1775)

*Pissodes strobi* (Peck, 1817)

**Subfamily SCOLYTINAE Latreille, 804**

**Tribe Corthylini LeConte, 1876**

Subtribe Corthylina LeConte, 1876

*Gnathotrichus materiarius* (Fitch, 1858)

Subtribe Pityophthorina Eichhoff, 1878

*Conophthorus coniperda* (Schwarz, 1895)

*Conophthorus resinosae* Hopkins, 1915

*Monarthrum mali* (Fitch, 1855)

Pityophthorus (Pityophthorus) angustus Blackman, 1928

Pityophthorus (Pityophthorus) balsameus Blackman, 1922

Pityophthorus (Pityophthorus) biovalis Blackman, 1922

Pityophthorus (Pityophthorus) briscoei Blackman, 1922

Pityophthorus (Pityophthorus) carinatus carinatus Bright, 1978

Pityophthorus (Pityophthorus) cariniceps LeConte, 1876

Pityophthorus (Pityophthorus) concavus Blackman, 1928

Pityophthorus (Pityophthorus) dentifrons Blackman, 1922

Pityophthorus (Pityophthorus) intextus Swaine, 1917

Pityophthorus (Pityophthorus) murrayanae Blackman, 1922

Pityophthorus (Pityophthorus) nitidus Swaine, 1917

Pityophthorus (Pityophthorus) opaculus LeConte, 1878

Pityophthorus (Pityophthorus) puberulus (LeConte, 1868)

Pityophthorus (Pityophthorus) pulchellus Eichhoff, 1869

Pityophthorus (Pityophthorus) pulicarius (C.C.A. Zimmermann, 1868)

*Pseudopityophthorus minutissimus* (C.C.A. Zimmermann, 1868

**Tribe Cryphalini Lindemann, 1877**

*Cryphalus ruficollis ruficollis* Hopkins, 1915

***Procryphalus mucronatus* (LeConte, 1879)**

*Trypophloeus populi* Hopkins, 1915

**Tribe Crypturgini LeConte, 1876**

*Crypturgus borealis* Swaine, 1917

*Crypturgus pusillus* (Gyllenhal, 1813)†

**Tribe Dryocoetini Lindemann, 1877**

*Dryocoetes affaber* (Mannerhiem, 1852)

*Dryocoetes autographus* (Ratzeburg, 1837)*

*Dryocoetes betulae* Hopkins, 1894

*Dryocoetes caryi* Hopkins, 1915

*Dryocoetes krivolutzkajae* Mandelshtam, 2001‡

*Lymantor decipiens* (LeConte, 1878)

**Tribe Hylastini LeConte, 1876**

*Hylastes opacus* Erichson, 1836†

*Hylastes porculus* Erichson, 1836

*Hylurgops rugipennis pinifex* (Fitch, 1858)

*Scierus annectans* LeConte, 1876

**Tribe Hylesinini Erichson, 1836**

*Hylesinus aculeatus* Say, 1824

**Tribe Hylurgini Gistel, 1848**

*Dendroctonus punctatus* LeConte, 1868

*Dendroctonus rufipennis* (Kirby, 1837)

*Dendroctonus simplex* LeConte, 1868

*Dendroctonus valens* LeConte, 1857

*Hylurgopinus rufipes* (Eichhoff, 1868)

*Xylechinus americanus* Blackman, 1922

**Tribe Ipini Bedel, 1888**

*Ips borealis* Swaine, 1911

***Ips grandicollis* (Eichhoff, 1868)**

*Ips perroti* Swaine, 1915

*Ips perturbatus* (Eichhoff, 1869)

*Ips pini* (Say, 1826)

*Orthotomicus caelatus* (Eichhoff, 1868)

*Orthotomicus latidens* (LeConte, 1874)

*Pityogenes hopkinsi* Swaine, 1915

*Pityogenes plagiatus plagiatus* (LeConte, 1868)

*Pityokteines sparsus* (LeConte, 1868)

**Tribe Phloeosinini Nüsslin, 1912**

*Phloeosinus canadensis* Swaine, 1917

**Tribe Phloeotribini Chapuis, 1869**

*Phloeotribus liminaris* (Harris, 1852)

*Phloeotribus piceae* Swaine, 1911

**Tribe Polygraphini Chapuis, 1869**

*Carphoborus carri* Swaine, 1917

*Carphoborus dunni* Swaine, 1924

*Polygraphus rufipennis* (Kirby, 1837)

**Tribe Scolytini Latreille, 1804**

*Scolytus multistriatus* (Marsham, 1802)†

*Scolytus piceae* (Swaine, 1910)

*Scolytus rugulosus* (P.W.J. Müller, 1818)†

**Tribe Xyleborini LeConte, 1876**

*Anisandrus dispar* (Fabricius, 1792)†

*Anisandrus obesus* LeConte, 1868

*Anisandrus sayi* (Hopkins, 1910)

***Xyleborinus attenuatus* (Blandford, 1894)**†

*Xyleborinus saxeseni* (Ratzeburg, 1837)†

**Tribe Xyloterini LeConte, 1876**

*Trypodendron betulae* Swaine, 1911

*Trypodendron lineatum* (Olivier, 1795)*

*Trypodendron retusum* (LeConte, 1868)

*Trypodendron rufitarsis* (Kirby, 1837)

*Xyloterinus politus* (Say, 1826)
